# Discovery of potent antimycobacterial agents targeting lumazine synthase (RibH) of *Mycobacterium tuberculosis*

**DOI:** 10.1038/s41598-024-63051-6

**Published:** 2024-05-28

**Authors:** Monica Singh, Anannya Dhanwal, Arpita Verma, Linus Augustin, Niti Kumari, Soumyananda Chakraborti, Nisheeth Agarwal, Dharmarajan Sriram, Ruchi Jain Dey

**Affiliations:** 1 Department of Biological Sciences, Birla Institute of Technology and Science Pilani, Hyderabad Campus, Hyderabad, Telangana 500078 India; 2 Translational Health Science and Technology Institute, Faridabad, Haryana 121001 India; 3 National Institute of Animal Biotechnology (NIAB), Hyderabad, Telangana 500032 India; 4 National Institute of Malaria Research, Indian Council of Medical Research (ICMR), New Delhi, 110077 India; 5 Department of Pharmacy, Birla Institute of Technology and Science Pilani, Hyderabad Campus, Hyderabad, Telangana 500078 India

**Keywords:** Drug discovery, Microbiology

## Abstract

Tuberculosis (TB) continues to be a global health crisis, necessitating urgent interventions to address drug resistance and improve treatment efficacy. In this study, we validate lumazine synthase (RibH), a vital enzyme in the riboflavin biosynthetic pathway, as a potential drug target against *Mycobacterium tuberculosis (M. tb)* using a CRISPRi-based conditional gene knockdown strategy. We employ a high-throughput molecular docking approach to screen ~ 600,000 compounds targeting RibH. Through in vitro screening of 55 shortlisted compounds, we discover 3 compounds that exhibit potent antimycobacterial activity. These compounds also reduce intracellular burden of *M. tb* during macrophage infection and prevent the resuscitation of the nutrient-starved persister bacteria. Moreover, these three compounds enhance the bactericidal effect of first-line anti-TB drugs, isoniazid and rifampicin. Corroborating with the in silico predicted high docking scores along with favourable ADME and toxicity profiles, all three compounds demonstrate binding affinity towards purified lumazine synthase enzyme in vitro, in addition these compounds exhibit riboflavin displacement in an in vitro assay with purified lumazine synthase indicative of specificity of these compounds to the active site. Further, treatment of *M. tb* with these compounds indicate reduced production of flavin adenine dinucleotide (FAD), the ultimate end product of the riboflavin biosynthetic pathway suggesting the action of these drugs on riboflavin biosynthesis. These compounds also show acceptable safety profile in mammalian cells, with a high selective index. Hence, our study validates RibH as an important drug target against *M. tb* and identifies potent antimycobacterial agents.

## Introduction

Tuberculosis (TB) remains a leading cause of death globally^1,2^. The current standard TB treatment regimen involves a combination of multiple drugs taken over a long duration of 6 to 9 months^1,2^. The lengthy treatment duration escalates the risk of patient non-compliance, leading to treatment failure and the development of drug resistance^1^. The mounting challenges with current anti-TB therapies have hindered the efforts to eliminate TB by 2030 and have created an urgent demand for the development of novel and impactful antibiotics^2^. *Mycobacterium tuberculosis* (*M. tb*) is highly vulnerable to developing resistance mutations against existing drugs^2,3^, as the majority of these drugs have one or few target(s) which allow pathogens to evolve mechanisms for evading or offsetting the inhibitory effects^3,4^. Hence, the development of drugs targeting new pathways is considered one of the important strategies to circumvent the current drug resistance mechanisms^5,6^. Further these new drugs may have the potential to yield synergism with existing first-line anti-TB drugs leading to enhanced treatment outcomes, allowing shorter, more efficacious regimens with lesser side-effects ^7^.

In this study, we focused on riboflavin biosynthetic pathway of *M. tb* that comprises of seven genes; *ribA1* (*Rv1940*), *ribA2* (*Rv1415*), *ribC* (*Rv1412*), *ribG* (*Rv1409*), *ribD* (*Rv2671*), *ribH* (*Rv1416*) and *ribF* (*Rv2786c*)^8–11^, out of which, five have been reported essential for the survival of the pathogen, namely, *ribA2*, *ribC*, *ribG*, *ribH* and *ribF* by transposon site hybridization (TraSH) screen ^8,9,12^. We have earlier shown that over-producing these essential genes reduce mycobacterial virulence by triggering a greater mucosal-associated invariant T cell (MAIT) response^8^. In addition to producing MAIT activating ligands, this pathway mainly produces essential redox cofactors, flavin mononucleotide (FMN) and Flavin adenine dinucleotide (FAD), which are required for activity of > 3% of the *M. tb* proteins ^13^. Recently, using a systems biology approach, Beste et al., predicted that FAD is a crucial cofactor with a significant role in respiratory metabolism, nucleotide biosynthesis, and amino acid metabolism^14,15^. FAD is also essential for the beta-oxidation of fatty acids, which is a crucial cofactor for mycolic acid and cell wall lipid biosynthesis and for metabolizing host-derived lipids^14^. Out of the 250 genes involved in fatty acid metabolism ^16^, in silico studies predict that ~ 70 mycobacterial gene products rely on FAD as a cofactor^14^. To explore the therapeutic potential of the riboflavin biosynthesis pathway, multiple attempts were made earlier ^17–23^, towards the structural analysis of RibH (lumazine synthase) and other enzymes of this pathway^24^ and identification of their ligands that could potentially bind to lumazine synthase active site thereby inhibiting the pathway. However, these studies, did not report the antimycobacterial activity of these compounds against *M. tb*. A recent study reported designing of riboflavin analogs targeting FMN riboswitch, showing inhibitory activity against *M. tb*, however these inhibitors were not designed to target lumazine synthase^17^.

We hypothesize that the riboflavin pathway could be a potential Achilles' heel and proteins involved in this pathway could serve as potential drug targets. Among various enzymes, RibH shows a high level of expression in the non-replicating persistence stage of *M. tb*^25^ and is essential for in vitro growth. These observations suggest a potential role of this enzyme in actively replicating as well as non-replicating dormant mycobacteria*.* In this study, we validate RibH as a potential drug target against *M. tb*. and report three highly potent compounds exhibiting promising standalone activity and conferring substantial en bloc enhancement in the anti-mycobacterial activity of rifampicin and isoniazid. Further, we report that all three inhibitors are non-toxic against human cells and possess a high selective index. Importantly, these molecules are highly effective against intracellular as well as non-replicating dormant bacteria.

## Results

### Conditional silencing by CRISPRi reveals the role of lumazine synthase in mycobacterial growth

**Figure 1:**
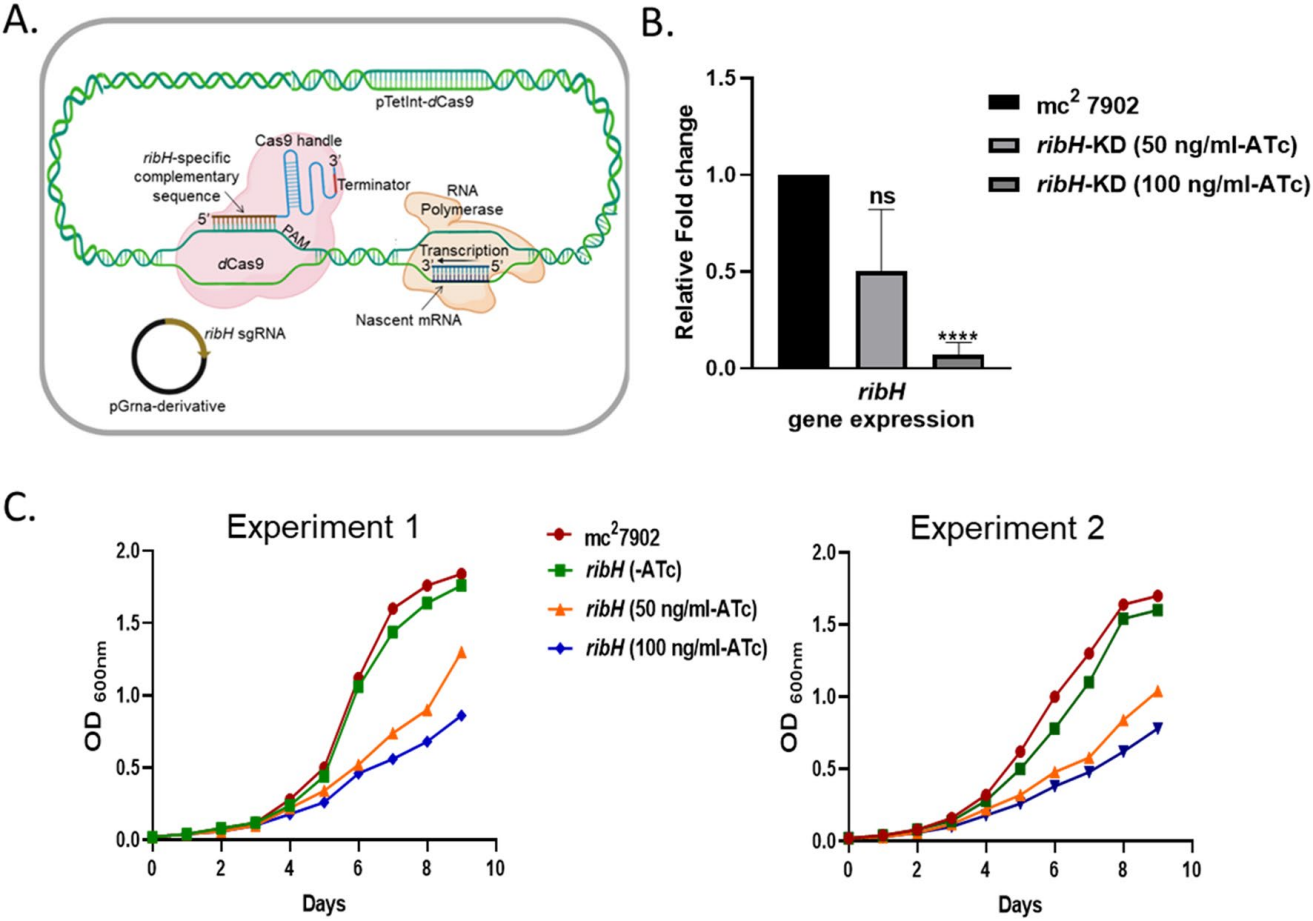
Validation of RibH as a potential drug target using CRISPRi. (**A**) CRISPRi design strategy. *ribH* gene (*Rv1416*) specific complementary oligonucleotides were designed and cloned as described in methods. The resulting strain was treated with anhydrotetracycline (ATc). (**B**) Quantitative RT-PCR to validate CRISPRi-mediated silencing of *ribH.* RT-PCR analysis was carried out showing reduced levels of ribH transcripts in *ribH*-KD in comparison to the parent *M. tb* mc^2^ 7902 at 50 and 100 ng/ml ATc. Fold change data (2^−ddCT^; n = 4) is plotted with respect to the parent strain. Fold change value is represented as mean ± s.d and statistical significance was measured using Student's unpaired t-test, *ns* non-significant, ****p < 0.0001. (**C**) Time-stamped growth curves of parent mc^2^ 7902 and *ribH*-KD at different concentrations of ATc (0, 50 and 100 ng/ml), *ribH*-KD shows delayed and reduced growth in experiments 1 and 2.

To determine the requirement of lumazine synthase encoded by *ribH (Rv1416*) for mycobacterial growth, we utilized the CRISPRi method to create knockdown strains of *M. tb* H37Rv mc^2^ 7902 (a pantothenate-leucine-arginine auxotroph, referred to as the parent strain in this study). The *M. tb* knockdown strain is conditionally depleted for *ribH* (annotated as *ribH*-KD) in an anhydrotetracycline (ATc)-dependent manner, as described earlier and in methods^29,30^. ATc induces production of dCas9 and *ribH* specific guide RNA that results in reduced expression of *ribH* gene (Fig. 1A). Following 9 days of treatment with 50 and 100 ng/ml ATc, we observe a notable decrease in the expression of *ribH* gene in *ribH*-KD (1.2 log_2_ and 7.5 log_2_ fold reduction with respect to the control strain, respectively; *p* < *0.05*) (Fig. 1B, Supplementary Fig. 1A). The impact of ATc induction on the growth of *ribH*-KD strain of *M. tb* is also evident from the growth curves assessed in the presence of ATc for 9 days (Fig. 1C). Growth defects are most evident from day 4 onwards, resulting in a 0.3-fold and 0.4-fold reduction in OD_600nm_ following incubation with 50 and 100 ng/ml of ATc, respectively. By day 9, these defects are accentuated, showing a marked reduction of 1.1-fold and 1.3-fold with 50 and 100 ng/ml of ATc, respectively, compared to the parent strain (Fig. 1C). These differences are found to be statistically significant at 50 ng/ml (*p* < *0.01*) and 100 ng/ml (*p* < 0.0001). However, *ribH*-KD strain of *M. tb* without ATc induction shows a similar growth profile as that of the parent strain. As a more consistent and pronounced reduction in gene expression of *ribH* and growth defects is observed at 100 ng/ml ATc, the same concentration was employed for all the follow-up experiments.

Additionally, we observed the downregulation of gene *Rv1417* following CRISPRi-mediated conditional silencing of *ribH* that likely stems from, the fact that *Rv1416* and *Rv1417* are co-transcribed^26^ however there is no off-target repression of genes other than the *ribH* operon (*Rv1416* and *Rv1417*) as evident from expression levels of genes such as, *ribA2* (*Rv1415*), *ribG* (*Rv1409*), and *ribF* (*Rv2786c*) (Supplementary Fig. 1).

## In silico molecular docking to identify compounds that could potentially bind RibH active site

**Figure 2:**
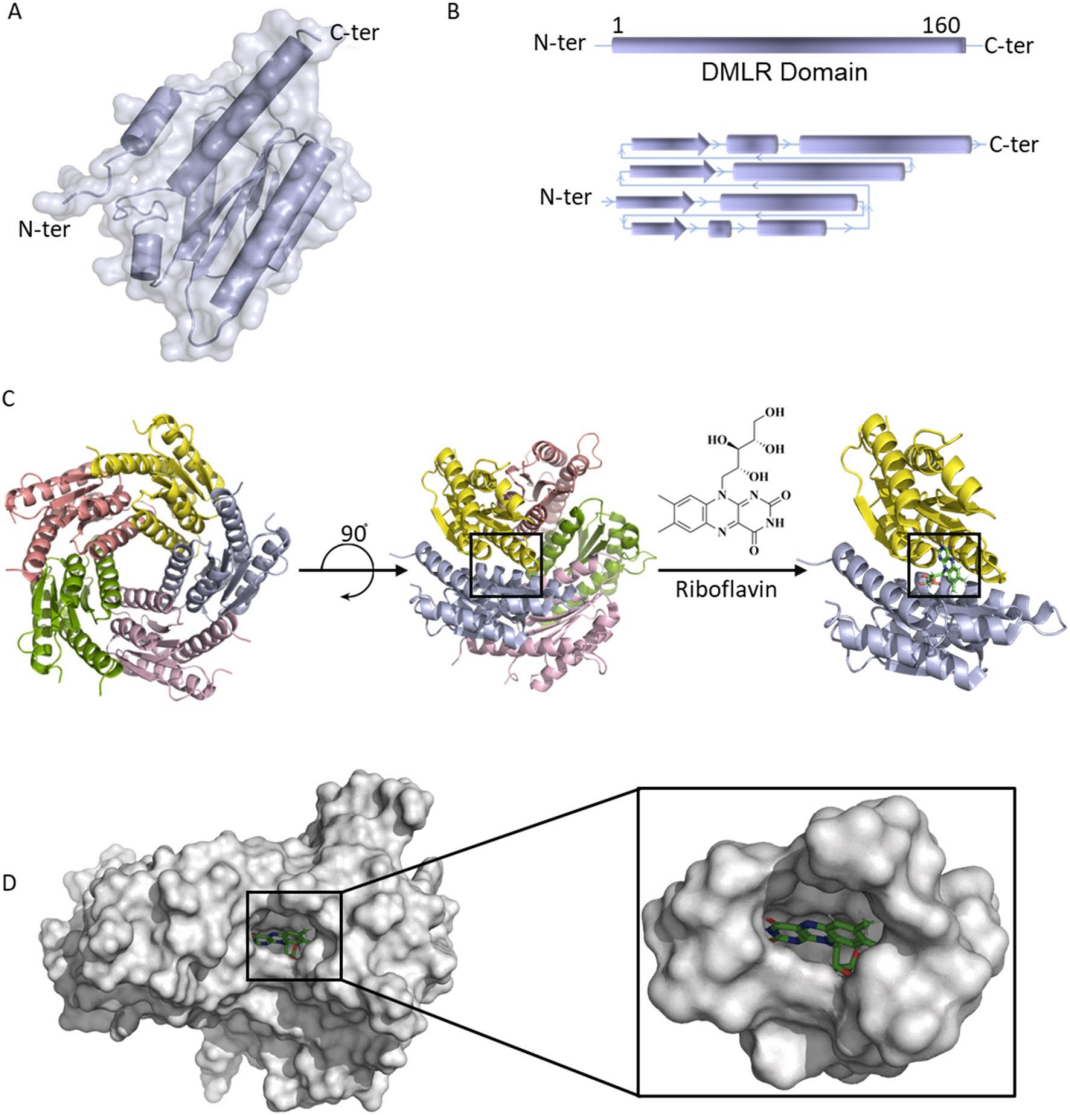
Structural characterization of *M. tb* RibH. (**A**) Surface and cartoon representation of one subunit of *M. tb* RibH depicting the secondary structure elements from the N-terminus to the C-terminus, (**B**) schematic representation of the DMRL (6,7-dimethyl-8-ribityllumazine synthase) domain (upper panel) and secondary structure organization of *M. tb* RibH that belongs to α/β/α sandwich topology, where alpha helices are represented as pipes, beta strands are represented as arrows (bottom panel) and (**C**) the homopentameric *M. tb* RibH (PDB ID 2C92) is shown as a cartoon with its active site present at the interface of two chains. Overall view of the *M. tb* RibH- riboflavin complex where the active site is occupied by riboflavin is shown, (**D**) surface representation of the *M. tb* RibH bound to riboflavin. Figure illustrates the substrate binding groove (cavity volume 889 Å^3^). The zoomed view shows the surface of the binding pocket with riboflavin buried inside.

Taking cues from the growth defects observed on conditional knockdown of the *ribH* gene and the inability of *M. tb* to secure this nutrient from outside at physiological concentrations, we next assessed the prospects of designing drugs targeting RibH, lumazine synthase. For this, we utilized the crystal structure of *M. tb* lumazine synthase complexed with TP6 (3-(1,3,7-trihydro-9-d-ribityl-2,6,8-purinetrione-7-yl) pentane 1-phosphate) (PDB ID 2C92 of 1.6 Å resolution)^19^. Structurally, *M. tb* lumazine synthase exists as a homopentamer belonging to the α/β family of proteins. Each subunit of *M. tb* RibH consists of 160 amino acid residues forming a three-layer α/β/α architecture (Fig. 2A) containing 6,7-dimethyl-8-ribityllumazine synthase (DMRL) domain (Fig. 2B). As reported earlier, the crystal structure of *M. tb* lumazine synthase has active sites present at the interface of the two adjacent subunits, making a total of 5 substrate binding sites^27^ (Fig. 2C). Visualization of the binding pocket using CB-Dock2 (https://cadd.labshare.cn/cb-dock2/php/show_cavity.php?user=guest&id=48021d02fdd749c1d59fdeadc97e610b&token=1708755782402) reveals a deep cavity of volume 889 Å^3^ consisting of twenty-nine amino acid residues viz., Ser 25, Trp 27, His 28, Leu 57, Gly 58, Ala 59, Ile 60, Glu 61, Val 81, Val 82, Ile 83, Arg 84, Gly 85, Gln 86, Thr 87, Pro 88, His 89, Phe 90, Val 93 of chain A and Ile 112, Ala 113, Asn 114, Gly 115, Arg 128, Glu 136, Lys 138, Gln 141, Ala 142 and Ala 145 of chain B. The dimension of the cavity along the x, y, and z axes is found to be 10, 14, and 16 Å, respectively (Fig. 2D). According to the literature, lumazine synthase can bind riboflavin in its active sites^28,29^. We re-docked the co-crystal ligands TP6 and riboflavin into the binding site and observed that these molecules are well fitted at the active site. Riboflavin penetrates deep inside into the *M. tb* RibH binding groove (Fig. 2D).

We next docked our *in-house* library consisting of ~ 3000 compounds and ~ 0.57 million compounds from the ASINEX screening library (Asinex Corp.), to identify the leads with the highest docking scores and desirable absorption, distribution, metabolism, and excretion (ADME) properties as described in methods. On molecular docking, we identify fifty-five compounds (40 from our *in-house* library and 15 from the Asinex library) (as listed in Supplementary Table 1) with the best binding orientations (see methods for details), docking scores, and ADME values. In silico analysis of the lead compounds targeting RibH predicts these compounds to possess good ADME properties (Table 1), bioavailability, gastrointestinal absorption and are also predicted to have a better cellular retention as they do not induce P-glycoprotein efflux pumps, unlike rifampicin^30–32^. Hence, RibH targeting drugs are predicted to have good pharmacokinetic properties with minimum drug-drug interactions and toxicity (Table 1).

**Table 1:** ADME properties of A10, A39 and A52.

Compound Code	ILOGP	XLOGP3	WLOGP	MLOGP	SILICOS-IT	Consensus LOG P_O/W_	Water solubility LOG S (ESOL)	Water solubility LOG S (Ali)	Water solubility LOG S (SILICOS-IT)	GI absorption	BBB permeant	Bioavailability score	P-glycoprotein substrate	CYP inhibition
A10	1.89	2.47	2.59	1.43	1.16	1.91	−3.52 (soluble)	−4.75 (moderately soluble)	−4.43 (moderately soluble)	High	No	0.55	No	CYP1A2 (Y), CYP2C19 (Y), CYP2C9 (Y), CYP2D6 (N), CYP3A4 (N)
A39	2.16	3.10	3.24	1.95	1.80	2.45	−4.12 (moderately soluble)	−5.41 (moderately soluble)	−5.03 (moderately soluble)	High	No	0.55	No	CYP1A2 (Y), CYP2C19 (Y), CYP2C9 (Y), CYP2D6 (N), CYP3A4 (N)
A52	2.30	2.83	2.90	1.69	1.66	2.28	−3.82 (soluble)	−5.13 (moderately soluble)	−4.81 (moderately soluble)	High	No	0.55	No	CYP1A2 (Y), CYP2C19 (Y), CYP2C9 (Y), CYP2D6 (N), CYP3A4 (N)

Interpretation note: as calculated by SwissADME^36^, all the compounds are predicted to have moderate to good solubility with high gastrointestinal (GI) absorption. The Lipophilicity is > 1 and < 5, indicating good oral absorption. Bioavailability of 0.55 indicates these drugs to be good candidates for further preclinical evaluation. None of the drug molecules are substrate for P-glycoprotein induction hence, are going to be bioavailable and have good bio distribution. As only 3 out of 5 CYP450 family of enzymes are inhibited suggesting that drugs may have high intracellular retention, but can be metabolized by the residual 2 CYP450 enzymes and can possibly be cleared by the renal system. They are anticipated to have low drug-drug interaction and low toxicity. None of the compounds are predicted to cross BBB—blood brain barrier.

The lead compounds selected by us in this study have not been reported earlier and hence, were selected for further assessment of antimycobacterial activity.

### Antimycobacterial activity of the short-listed compounds

**Figure 3:**
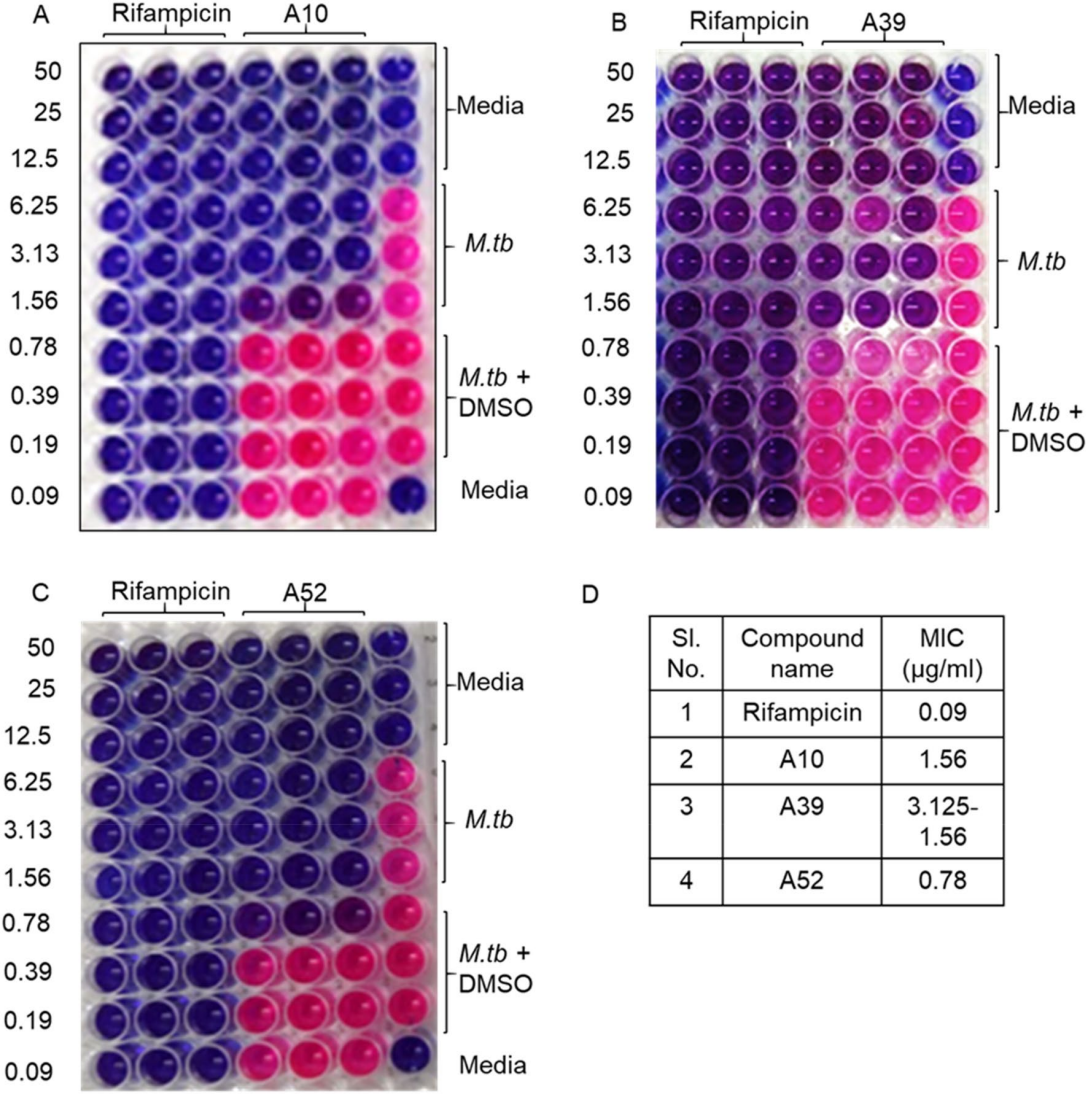
Anti-mycobacterial activity of shortlisted RibH inhibitors. The figure depicts the representative pictograms obtained on screening of anti-mycobacterial activity of three of the shortlisted compounds namely, A10, A39 and A52 against *M. tb* H37Rv by microplate Alamar blue assay (MABA). The assay was performed at least three times with three technical replicates in each case. Rifampicin and shortlisted compounds were tested at various concentrations between 50 and 0.09 µg/ml (two-fold serial dilution). In our assay rifampicin exhibited an MIC of 0.09–0.048 µg/ml. (**A**) Compound A10 exhibited an MIC of 1.56 µg/ml, (**B**) compound A39 exhibited an MIC of 3.125–1.56 µg/ml, (**C**) compound A52 exhibited an MIC of 0.78 µg/ml. (**D**) Tabulation of MIC values obtained for various compounds with respect to rifampicin. The experiments were performed at three times with three technical replicates.

After the preliminary high-throughput virtual screening, fifty-five short-listed compounds were screened against *M. tb* H37Rv strain to evaluate their minimum inhibitory concentration (MIC) using microplate Alamar Blue assay (MABA) (Fig. 3A–C). Among the 9 compounds showing MIC in the range of 0.78–12.5 µg/ml in the primary screen, A52 is found to be the most potent with an MIC of 0.78 µg/ml (Fig. 3 C, D, Supplementary Table 1), whereas the MIC of both A10 and A39 is 1.56 µg/ml. Out of the 55, 12 compounds show moderate anti-tubercular activity in the range of 25–50 µg/ml and thirty-four show poor activity with MIC > 50 µg/ml, despite the high docking score (Supplementary Table 1).

### Characterization of the top hits exhibiting anti-mycobacterial activity

As shown in Supplementary Table 1, we identified three compounds, namely A10 (5-((5-nitrofuran-2-yl)methylene)-2-(phenylimino)thiazolidin-4-one), A39 (2-((4-chlorophenyl)imino)-5-((5-nitrofuran-2-yl)methylene)thiazolidin-4-one) and A52 (5-((5-nitrofuran-2-yl)methylene)-2-(*p*-tolylimino)thiazolidin-4-one) which exhibit the best minimum inhibitory concentration (MIC) among all the 55 compounds tested against *M. tb* strain H37Rv in the range of 0.78–1.56 µg/ml (Supplementary Table 1). Strikingly, these compounds appear to be similar in their basic structure (see supplementary methods), with minor modifications in the R group resulting in different binding attributes (for synthesis scheme, see supplementary methods). The compounds namely, A10, A39, and A52, show a docking score of −7.276, −6.825, and −8.431 kcal/mol, respectively, signifying that these molecules can tightly bind to the RibH active site (Supplementary Table 1).

**Figure 4:**
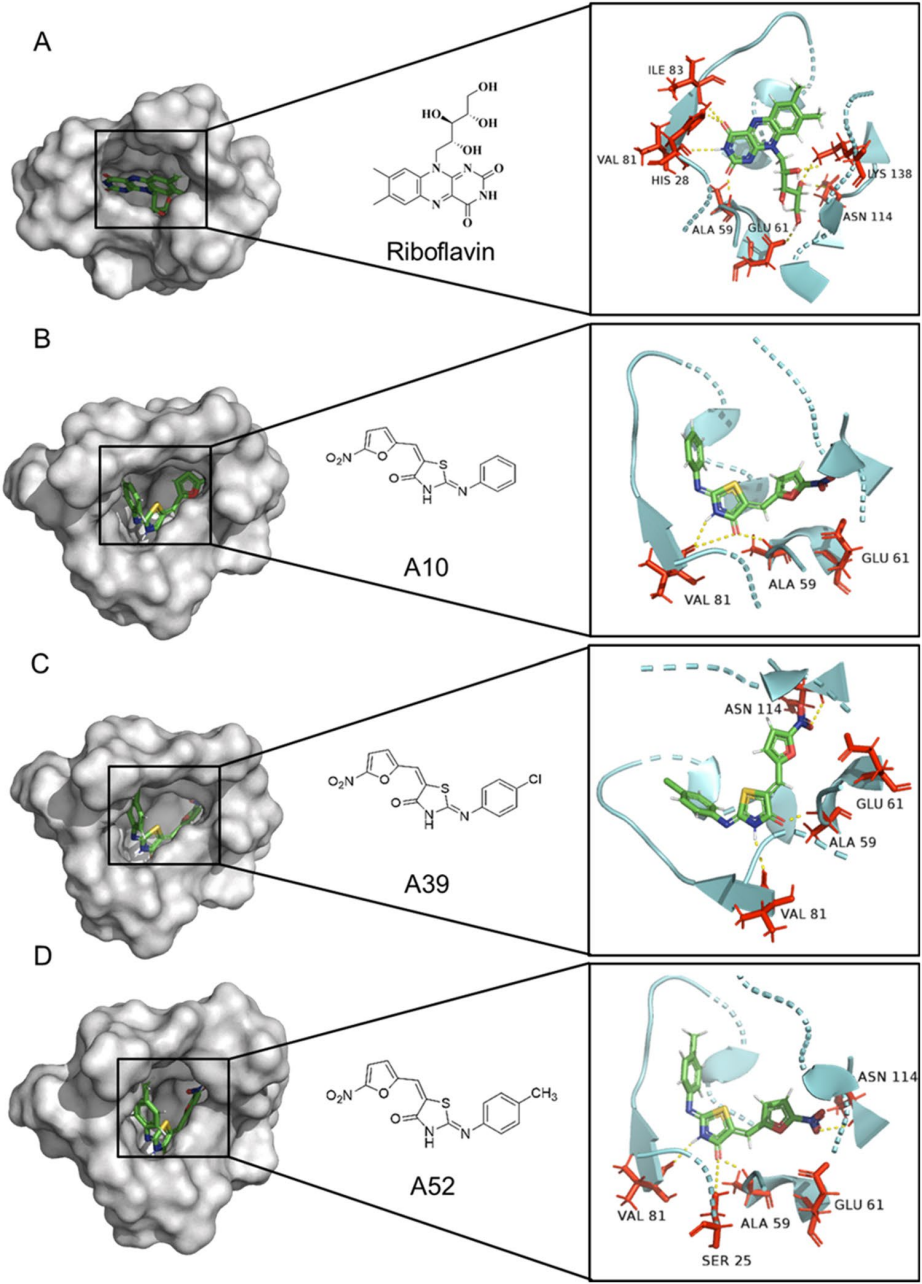
Active site surface representation and amino acid interactions of *M. tb* RibH with riboflavin and shortlisted drug molecules. Interaction of amino acids in the RibH active site are shown for (**A**) Riboflavin, (**B**) A10, (**C**) A39 and (**D**) A52. The zoomed view shows the bound fragment in the active pocket, wherein, (**A**) Riboflavin interacts with His 28, Ala 59, Glu 61, Val 81, Ile 83, Asn 114 and Lys 138, (**B**) A10 interacts with Ala 59, Glu 61 and Val 81, (**C**) A39 interacts with Ala 59, Glu 61, Val 81 and Asn 114, (**D**) A52 interacts with Ser 25, Ala 59, Glu 61, Val 81 and Asn 114. Hydrogen bonds are shown as yellow dashed lines.

As shown in Fig. 4 riboflavin binding pocket analysis reveals that, riboflavin forms several hydrogen bonds with amino acid residues His 28, Ala 59, Glu 61, Val 81, Ile 83 of chain A, and Asn 114 and Lys 138 of chain B (Fig. 4A). Binding pocket analysis for the short-listed compounds, namely, A10, A39 and A52 reveals multiple interactions of these compounds with the active site amino acids (Supplementary Table 1). Interaction of A10 with the binding groove shows formation of two hydrogen (H) bonds (Fig. 4B). The amide-carbonyl of A10 interacts with Ala 59 and the amide-NH interacts with Val 81 forming H-bonds of bond length 2.28 Å and 2.33 Å, respectively. In addition, the furan nitro (-NO2) group of A10 forms a salt bridge of bond length 3.79 Å with Glu 61 **(**Fig. 4B**)**. The furan nitro (-NO2) group of A39 interacts with Asn 114 of chain B forming an H-bond of length 2.46 Å, and the same group also interacts with Glu 61 of chain A forming a salt bridge of bond length 3.75 Å. Similar to A10, the amide-carbonyl and the amide-NH form H-bonds with Ala 59 and Val 81 of lengths 2.07 Å and 2.55 Å, respectively. Additionally, the chloride (-Cl) group attached to the benzene ring interacts with Thr 87 to form a halogen bond of length 2.64 Å (Fig. 4C). A52 interacts with the binding pocket to form four H-bonds. The furan nitro (-NO_2_) group forms an H-bond with Asn 114 of length 2.72 Å and a salt bridge of 3.56 Å with Glu 61. The amide-carbonyl forms H-bonds with Ser 25 and Ala 59 of lengths 3.5 Å and 2.05 Å, respectively. The amide-NH group interacts with Val 81 forming a H-bond of length 2.13 Å (Fig. 4D).

## Microscale thermophoresis (MST) reveal a high binding affinity of the shortlisted compounds with purified lumazine synthase.

We next assessed the binding affinity of the three shortlisted compounds with the purified lumazine synthase by determining the dissociation constant (K_d_) with the help of MST assay in vitro. Details of cloning, purification, and characterization of RibH protein utilized in this assay are described in the Supplementary methods.

**Figure 5:**
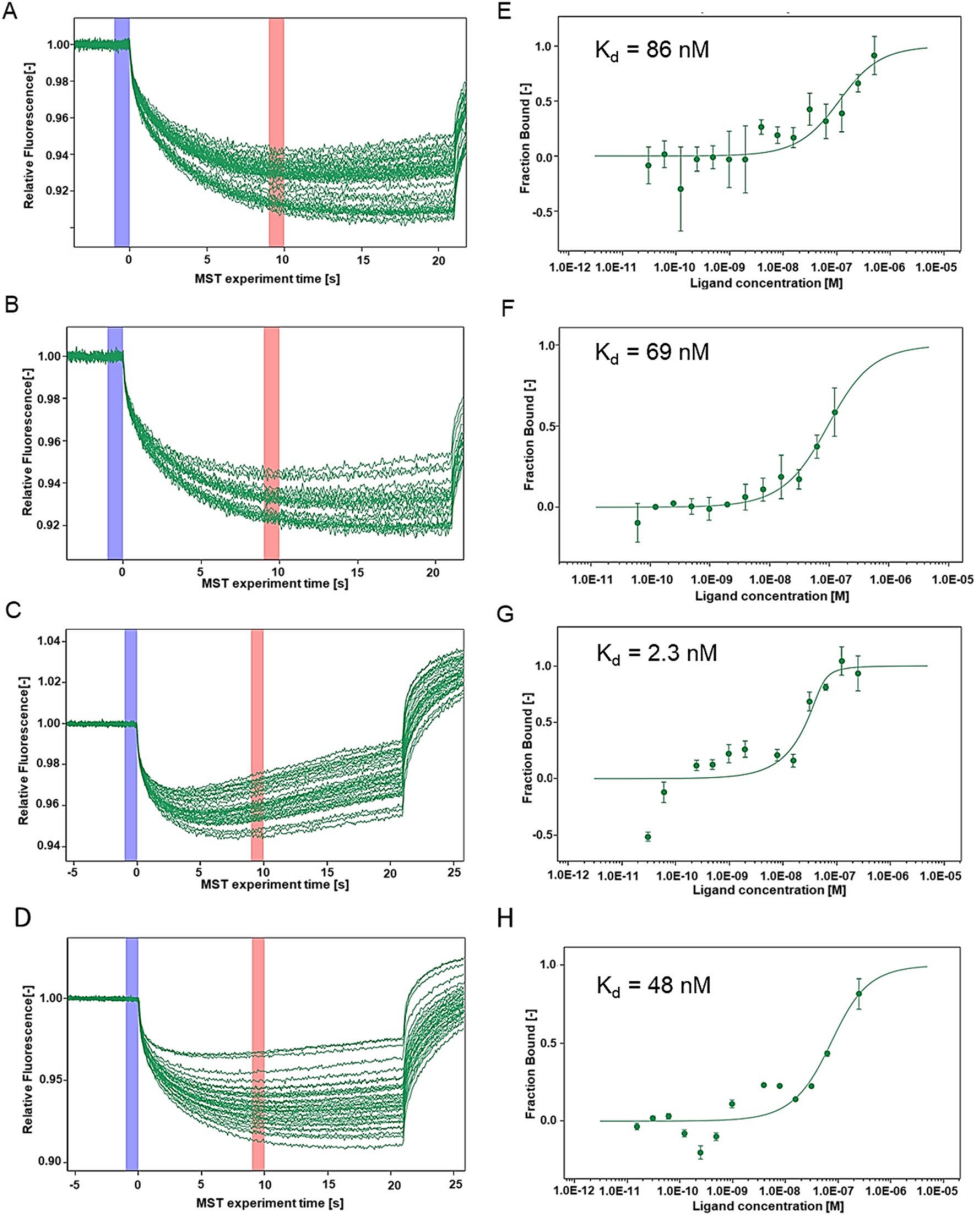
Microscale thermophoresis (MST) analysis of RibH protein with riboflavin and shortlisted drug molecules. Binding affinity of riboflavin and shortlisted drug molecules to RibH purified protein was determined by studying protein–ligand interaction using 16 concentrations (500–0.015 nM) of riboflavin or shortlisted drug molecules namely, A10, A39 and A52 with a fixed concentration of fluorescently labelled *M. tb* RibH protein (50 nM). MST traces corresponding to various concentrations are illustrated for, (**A**) Riboflavin, (**B**) A10, (**C**) A39 and (**D**) A52. Regression curves were generated to determine the bound fraction of the ligands, thereby calculating the binding affinity as K_d_ values. Binding affinity were determined to be (**E**) 86 nM for riboflavin, (**F**) 69 nM for A10, (**G**) 2.3 nM for A39 and (**H**) 48 nM for A52. The data for three independent experiments (n = 3) is presented as mean ± s.d.

In the assay conditions described, MST analysis reveal that riboflavin, A10, A39, and A52 bind to lumazine synthase with a binding affinity (K_d_) of 86 nM, 69 nM, 2.3 nM, and 48 nM, respectively (Fig. 5A–H).

## In vitro riboflavin displacement assay reveals specific binding of the short-listed compounds to RibH active site

We next verified if the short-listed compounds bind specifically to the active site of RibH as predicted by our in-silico analysis. For this, a simple and sensitive assay developed by Chen et al. ^29^ is utilized. The assay is based on the displacement of the bound riboflavin (non-fluorescent) from the active site by a compound competing for the same binding site resulting in release of the free riboflavin into the solution that fluoresces at Ex_440nm/_Em_530nm_. First, 27.04 µM of riboflavin is pre-complexed with 28 µM of RibH protein as described in Supplementary methods (~ 0.966 times molar ratio of riboflavin w.r.t the protein concentration). The complex is then diluted to a final concentration of 4 µM and the short-listed compounds are added at a concentration of 12.5 µg/ml (Supplementary methods) followed by fluorescence measurement (n = 8). Under the given test conditions, the three compounds, namely, A10, A39 and A52 exhibit ~ 17.5%, ~ 25.5% and ~ 19.98% displacement of riboflavin from the RibH protein, respectively (Supplementary Fig. 2) indicating the ability of these compounds to specifically compete and displace riboflavin bound to *M. tb* RibH protein from the active site. Moreover, displacement caused by these compounds corroborates the MST observations.

### Reduced FAD^+^ levels in *M. tb* treated with RibH binding ligands

To further establish the specific action of RibH binding compounds as inhibitors of the *M. tb* riboflavin biosynthesis, intracellular FAD^+^ levels are measured for the untreated and the treated H37Rv cultures by high-performance liquid chromatography (HPLC). Following treatment of *M. tb* H37Rv with the short-listed compounds, extracted nucleotides are subjected to HPLC analysis, wherein, FAD^+^ peaks are monitored on photodiode array detector and identified by their retention times (~ 1.9 min) at a wavelength of 450 ± 2 nm with reference to a solution of FAD^+^ as standard (Supplementary Fig. 3A–E). Treatment of *M. tb* with shortlisted compounds resulted in reduced intracellular levels of FAD^+^ compared to the untreated *M. tb* control group (Supplementary Fig. 3F, G). Particularly, treatment with A10, A39 and A52 resulted in markedly reduced absorbance (mAU) values and area under curve (AUC) represented as area ratio (with respect to internal control cXMP) compared to the untreated *M. tb* (Supplementary Fig. 3E). Among the three compounds, A52 exhibit the maximal reduction in FAD^+^ levels compared to the other two compounds, which corroborates well with its highest in vitro anti-mycobacterial activity (Fig. 3C). Hence, reduced levels of FAD^+^ on treatment with RibH binding compounds, MST derived binding affinity and riboflavin displacement assay support our hypothesis that compounds discovered in this study, act through specific binding and inhibition of lumazine synthase (RibH) and riboflavin biosynthesis of *M. tb* leading to reduced production of the final product FAD^+^, which leads to the anti-mycobacterial activity of these compounds.

### Drugging lumazine synthase results in synergistic action with the first-line anti-TB drugs

**Figure 6:**
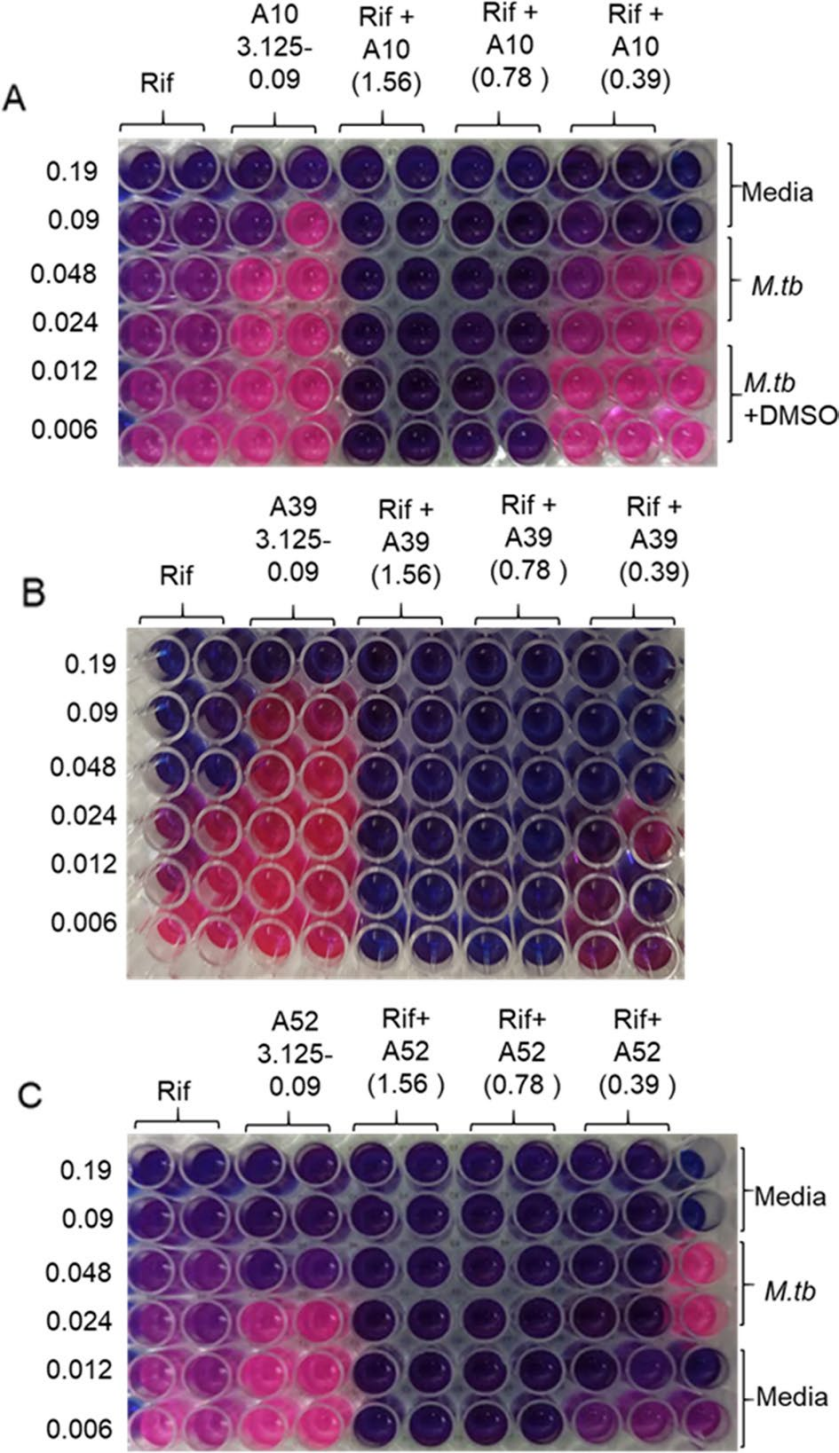
Anti-mycobacterial activity of short-listed compounds in combination with first-line anti-TB drug rifampicin. The figure depicts the representative pictograms obtained on screening of anti-mycobacterial activity of three of the shortlisted compounds against *M. tb* H37Rv in combination with rifampicin by MABA*.* The assay was performed at least twice with two technical replicates in each case. In each case (**A–C**) rifampicin was tested at various concentrations between 0.19 and 0.006 µg/ml (two-fold serial dilution) either alone or in combination with various concentrations of (**A**) A10, (**B**) A39 and (**C**) A52. These shortlisted compounds were either tested alone at various concentrations between 3.125 and 0.09 µg/ml (two-fold serial dilution) or at their respective MICs and sub-MIC concentrations in combination with various concentrations of rifampicin (MIC to sub-MIC range). A synergistic enhancement in antimycobacterial activity of rifampicin and shortlisted compounds was observed as described in Table 2 (for combined anti-TB activity with Isoniazid see supplementary Fig. 4).

We sought to determine if: (i) the anti-mycobacterial action of A10, A39, and A52 can be enhanced by combining with the first-line anti-TB drugs, and (ii) the addition of these compounds at the sub-MIC level increases *M. tb* susceptibility to the first-line anti-TB drugs. For this, a checkerboard MABA^33^ was employed, wherein A10, A39, and A52 were tested at various concentrations above and below MIC in combination with different concentrations at or below MIC of rifampicin (0.19–0.006 µg/ml) (Fig. 6A–C) and isoniazid (0.39–0.012 µg/ml) (Supplementary Fig. 4A–C) against *M. tb* H37Rv. Strikingly, the addition of all three RibH-targeting drugs show synergistic action with rifampicin dramatically improving its MIC to ≤ 0.006 µg/ml, with 15-, 8-, and 7.5-fold reduction by A10, A39, and A52, respectively. The addition of rifampicin also enhances the antimycobacterial activity of A10, A39, and A52 resulting in a reduction in MIC by 4-, 4-, and 2-fold, respectively. Similarly, A10, A39, and A52 improve the efficacy of isoniazid with 15.8-, 32.5- and 15.8-fold reduction in MIC, respectively. Table 2 depicts the fractional inhibitory concentration index (FICI), wherein combining these compounds with rifampicin and isoniazid results in synergistic action with FICI ≤ 0.5.

**Table 2:** Synergistic interactions between RibH targeted drugs and first line anti-TB drugs rifampicin and isoniazid.

Name of the short-listed compounds	Stand-alone and combination MIC (µg/ml) of the shortlisted compounds with rifampicin or isoniazid	FIC_A_ (FIC of short-listed compounds with rifampicin or isoniazid)	Stand-alone and combination MIC (µg/ml) of rifampicin or isoniazid with short-listed compounds	FIC_B_ (FIC of rifampicin or isoniazid with short-listed compounds)	FICI for rifampicin or isoniazid = FIC_A_ + FIC_B_	Fold reduction in MIC of rifampicin or isoniazid
Rifampicin						
A10	#1.56, *0.39	0.25	#0.09, *0.006	0.066	0.31	15
A39	#1.56, *0.7	0.249	#0.048, *0.006	0.125	0.37	8
A52	#0.78, *0.39	0.5	#0.09, *0.012	0.13	0.6	7.5
Isoniazid						
A10	#3.125, *0.39	0.124	#0.19, *0.012	0.06	0.18	15.8
A39	#3.125, *0.39	0.124	#0.39, *0.012	0.03	0.154	32.5
A52	#0.78, *0.19	0.24	#0.19, *0.012	0.06	0.3	15.8

Table describes degree of interaction of pairwise combinations of rifampicin and isoniazid in combination with A10, A39 and A52 against *M. tb *H37Rv. Fractional inhibitory concentration index (FICI) was calculated using formula = FIC_A_ + FIC_B._

FIC_A_ = MIC of compound A in combination with B/MIC of compound A alone.

FIC_B_ = MIC of compound B in combination with A/MIC of compound B alone.

Synergy is defined as FICI of ≤ 0.5, antagonism as ≥ 4. #, Stand-alone MIC of the compound; *, MIC of the compound in combination; Fractional inhibitory concentration (FIC) for each compound is calculated considering the observed MICs of rifampicin/isoniazid and the short-listed compounds in each specific experiment.

### Cytotoxicity and selectivity index of A10, A39, and A52

Based on the anti-mycobacterial activity, the three compounds were further selected for assessment of cytotoxicity and selectivity index using 3-(4,5-dimethylthiazol-2-yl)-2,5-diphenyltetrazolium bromide (MTT) assay, as described in methods. The cytotoxicity was assessed in human embryonic kidney 293 (HEK293t) cells. Among the three potent compounds, A39 shows the least cytotoxicity at MIC, followed by A10 and A52. However, A52 displays the highest IC_50_ of 100 µg/ml corresponding to a selectivity index (SI) of 128 (Table 3). In contrast, A10 and A39 exhibit IC_50_ of 50 and 56 µg/ml, and a corresponding SI of ~ 32 and ~ 36, respectively. Most, importantly, at the MIC level all three compounds show minimal toxicity, (13.5%, 0%, and 19.82% by A10, A39, and A52, respectively) (Table 3).

**Table 3:** In vitro cytotoxicity and selectivity index of compounds A10, A39 and A52.

Compound	MIC against H37Rv (µg/ml)	% survival at MIC in HEK293t	IC_50_ in HEK293t cells (µg/ml)	Selectivity index (HEK293t)
A10	1.56	86.5	50	32
A39	1.56	100	50	32
A52	0.78	80.18	> 50	> 64
Rifampicin	0.09	100	> 50	> 500

Cytotoxicity of the RibH targeting drugs was measured by MTT assay against human embryonic kidney (HEK293t) cells and data (n = 3) was analysed and expressed as IC_50_ (half-maximal inhibitory concentration). Selectivity index (SI) = IC_50_ (mammalian cells)/MIC (*M. tb *H37Rv). For THP1 related IC_50_, see supplementary Table 5.

### Intracellular growth reduction of *M. tb* by RibH inhibitors

**Figure 7:**
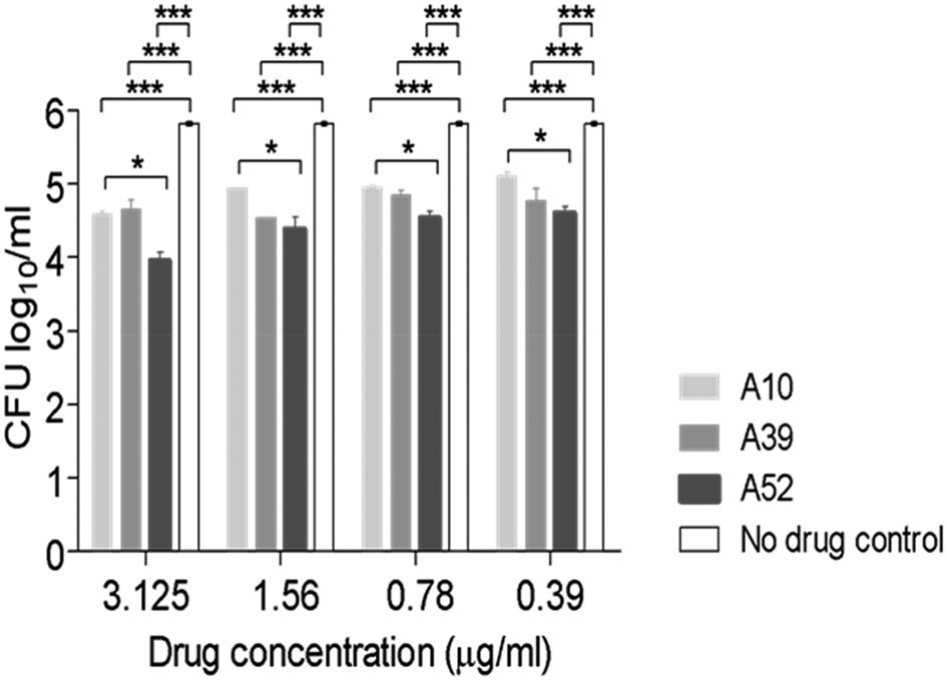
RibH targeting drugs reduce the intracellular growth of *M. tb.* in macrophages. Intracellular survival of *M. tb *H37Rv in THP-1 derived macrophages post 5-days treatment with three different concentrations of A10, A39 and A52. THP-1 cells were infected with *M. tb. *H37Rv﻿ at an MOI of 1:5. Infected macrophages were treated with A10 or A39 at a concentration above MIC (3.125 µg/ml), MIC (1.56 µg/ml) and sub-MIC (0.78 µg/ml); or A52 at two concentrations above MIC (3.125 µg/ml-1.56 µg/ml) and MIC (0.78 µg/ml). CFU data (n = 3) is represented as mean±s.d log_10 _CFU/ml. All the three compounds gave significant growth inhibition as compared to untreated control (***p < 0.0001, *p < 0.05; two-tailed Student’s t-test using GraphPad Prism software) (for detailed CFU see supplementary Table 6).

To understand if these potent molecules can traverse the barrier of the host cell membrane to effectively reduce the intracellular growth of mycobacteria inside the macrophages, the antimycobacterial activity of A10, A39, and A52 is examined at various concentrations above and below the MICs (3.125, 1.56, 0.78 and 0.39 µg/ml), as described in methods. Infection of human macrophage line THP-1 by *M. tb* H37Rv and drug treatment is performed for 5 days, and on the 6^th^ day infected macrophages are lysed and colony forming units (CFU) are determined. Our results show that all the compounds are highly effective in reducing the growth of *M. tb,* as evident from significant decrease in colony forming units (CFU) in the case of A10, A39, and A52, respectively, compared to the untreated infected cells (*p* < 0.0001) (Fig. 7, Supplementary Table 6) Importantly, A52 is found to be the most potent among all the compounds that we tested across various concentrations causing 1.2–1.9 log_10_ reduction in CFU as compared to A39 (1.09–1.2 log_10_ reduction in CFU) and A10 (0.73–1.2 log_10_ reduction in CFU).

### Lumazine synthase targeting drugs interfere with resuscitation in an in vitro dormancy model

**Figure 8:**
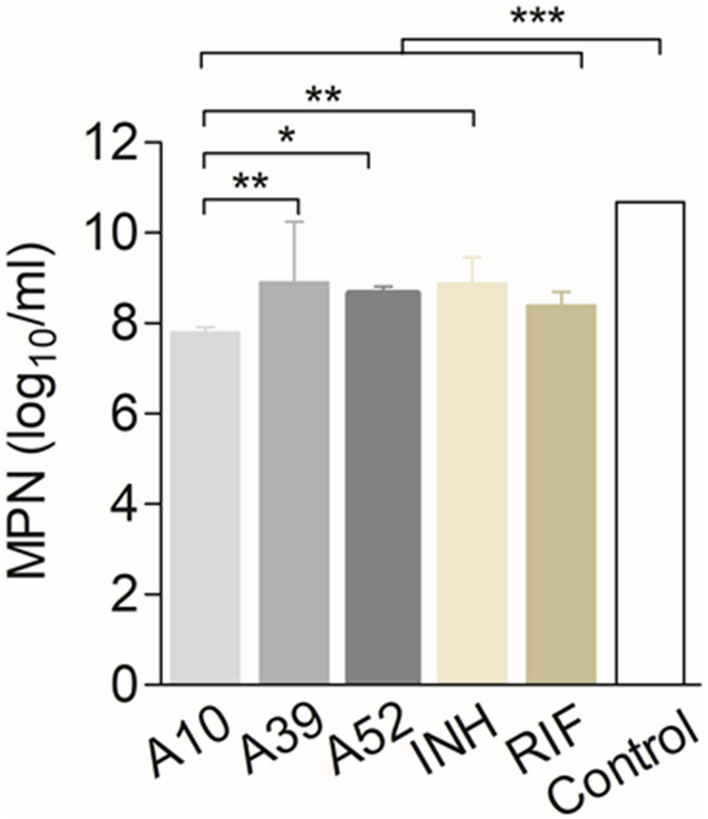
RibH targeting drugs interfere with growth (resuscitation) of nutritionally starved *M. tb* in an in vitro dormancy model. *M. tb*. H37Rv cultures were starved for 6 weeks in phosphate buffered saline, followed by treatment with the compounds namely, A10, A39 and A52 or first-line anti-TB drugs isoniazid or rifampicin at an equimolar concentration of 10 µM. Bacterial count estimation was carried out using the MPN (most probable number) assay. CFU data (n = 3) is represented as geometric mean ± SEM log_10 _CFU/ml. Data represents geometric mean with 95% CI for (n = 3). All the three compounds gave significant growth inhibition as compared to untreated control (*p* < 0.0001, two-tailed t-test using GraphPad Prism software). Compound A10 shows significant reduction in *M. tb* growth as compared to the first line anti-TB drug Isoniazid (*p *< 0.05, one-way ANOVA using GraphPad Prism software).

We next tested, if RibH targeting drugs function against nutritionally starved *M. tb* in an in vitro dormancy/non-replicating persistence model (Supplementary Fig. 5), and whether these drugs interfere with the ability of the bacteria to resuscitate when exposed to the nutrient-rich conditions. We mimicked dormancy by starving *M. tb* H37Rv in phosphate-buffered saline for a period of six week, and then exposed the bacteria to A10, A39, and A52 with rifampicin and isoniazid as control drugs. We followed *M. tb* growth by most probable number (MPN) assay, as described in the methods. Remarkably, our findings reveal that these drugs not only act upon the nutritionally starved bacteria but also interfere with their ability to resuscitate despite the presence of nutrient-rich conditions, which otherwise favours bacterial growth in the absence of drugs. All the compounds show potent activity against nutritionally starved *M. tb* H37Rv (Fig. 8), however, A10 causes maximum growth inhibition resulting in ~ 2.9 log_10_ reduction in CFU compared to that of untreated control (*p* < 0.0001). Noteworthy to mention, A10 performs better than the first-line anti-TB drugs, resulting in 0.6 log_10_ and 1.081 log_10_ fewer bacillary counts compared to rifampicin and isoniazid (*p* < 0.01), respectively (Fig. 8). A10 also shows better activity compared to A39 (*p* < 0.01) and A52 (*p* < 0.05). Treatment with A52 and A39 results in 2.02 log_10_ and ~ 1.8 log_10_ reduction, respectively, compared to the untreated control (*p* < 0.0001). However, the potency of A52 and A39 is comparable to that of rifampicin (2.29 log_10_ reduction) and isoniazid (1.8 log_10_ reduction).

### RibH-a highly conserved drug target across various mycobacterial species

Having established the essentiality, druggability, synergism with existing drugs and safety profile of drugs targeting RibH of *M. tb* we next analysed if RibH is conserved during evolution among various mycobacterial species belonging to *Mycobacterium tuberculosis* complex (MTC) and Non-tuberculous mycobacteria (NTM).

We first conducted multiple sequence alignment of RibH protein for various mycobacterial species and determined the phylogenetic relationship between them (Supplementary Figs. 6 and 7). RibH protein was found to be highly conserved in nature across the MTC, NTM, and *Mycobacterium leprae* (*M. leprae*) with minimum divergence. We then specifically compared the crucial amino acids present in the drug binding and co-crystal ligand binding pocket across various mycobacterial species. Remarkably, the binding site for A10, A39, A52, and TP6 was found to be highly conserved (95–100% identity) across the mycobacterial species (Supplementary Table 2). Especially, major mycobacterial pathogens were found to have 100% identity in the drug-binding pocket, thereby increasing the likelihood of broad-spectrum activity of these drugs for treating various other mycobacterial infections. Interestingly, drug binding pocket was also found to be conserved in *M. leprae* (causative agent of leprosy in humans and belonging to *Mycobacterium leprae *complex MLC). The conserved nature of the RibH protein and drug binding pocket increases the translational potential of the drugs developed by us, making it suitable for future testing against MTB, NTMs, and MLC with broad spectrum applications for treating human and animal diseases caused by mycobacteria.

## Discussion

This study describes a comprehensive investigation into the role of lumazine synthase (encoded by *ribH*) in mycobacterial growth and the potential for therapeutic targeting of *M. tb* RibH towards developing new anti-TB agents. In this study we also utilize CRISPRi to validate the drug target by conditionally silencing the *ribH* gene in *M. tb* leading to significantly reduced *ribH* expression and growth defects. The growth defects observed in the *ribH* knockdown strain were rescued by addition of an excess amount of exogenous riboflavin (although, much above the physiological concentration). These observations highlight the key role of riboflavin biosynthesis for mycobacterial survival and confirm specific nature of CRISPRi based interference of this pathway via lumazine synthase.

We then identified drugs targeting the riboflavin biosynthesis pathway, a crucial source of important cofactors- FAD and FMN in *M. tb*. Among the various enzymes involved in riboflavin biosynthesis, we identified RibH to be the most suitable candidate for several reasons. First, it was found to be highly conserved across the evolutionary scale of mycobacteria (Supplementary Figs. 6 and 7 and Supplementary Table 2). With only a single copy of this gene in the *M. tb* genome and no evidence for redundancy in its enzymatic function, interference at the penultimate step of riboflavin synthesis catalysed by lumazine synthase is anticipated to substantially curtail the pathway. The reduced levels of FAD^+^ observed in our study on treating *M. tb* with the short-listed RibH inhibitors, antimycobacterial action of these drugs and reduced growth of *ribH*-KD further substantiate our hypothesis. The latter, also corroborates well with the *ribH* gene essentiality reported in the TraSH screens^9,12^. Considering the crucial role of cofactors derived from this pathway in diverse metabolic functions, interference of this enzyme employing specific drug molecules possibly makes *M. tb* metabolically unfit leading to cell death.

In this study, our high-throughput molecular docking approach allowed us to screen ~ 0.6 million compounds capable of binding to RibH. Through this screening process, we discovered three highly potent compounds that exhibit promising antimycobacterial activity against the *M. tb* H37Rv as standalone drug. In absence of commercially available substrates of lumazine synthase, while direct enzymatic inhibition assays could not be performed, we employ alternative step-by-step approach, to provide conclusive evidence for (i) binding of these short-listed compounds to RibH with high affinity using MST, (ii) specific binding of these compounds to the active site by competitively displacing riboflavin, its natural ligand by riboflavin displacement assay and (iii) metabolic inhibition of riboflavin biosynthetic pathway causing diminished production of FAD^+^, the end product of the pathway employing HPLC based relative estimation.

The most noteworthy finding of our research was the potential synergism of these compounds with the first-line anti-TB drugs, rifampicin, and isoniazid in vitro. Although, the detailed mechanism of action of drugs are currently not known to us, future studies to understand the changes in mycobacterial cell wall, and other transcriptional and metabolic profiles will provide mechanistic insights into the increased vulnerability of *M. tb* to the first-line anti-TB drugs on exposure to RibH targeting drugs.

The first-line anti-TB drugs are known for their high toxicity and side effects in 30–40% of the patients, resulting in non-compliance to TB therapy^34,35^. In silico analysis of lead compounds targeting RibH predicts these compounds to possess good ADME properties (Table 1), bioavailability, and gastrointestinal absorption and are also predicted to have better cellular retention as they do not induce P-glycoprotein efflux pumps, unlike rifampicin^30–32^. Hence, RibH-targeting drugs are predicted to have good pharmacokinetic properties with minimum drug-drug interactions and toxicity (Table 1 and Table 3). Possibly a better cellular retention due to non-induction of P-glycoprotein^36^ would have contributed to an effective intracellular mycobacterial clearance as observed in this study (Fig. 7 and Table 3). Our study also reveals that when drugs were tested in the presence of riboflavin at physiological concentrations, there was no significant change in the respective MICs (Supplementary Fig. 8), indicating the limited ability of *M. tb* to restore growth post-drug treatment. Additionally, exogenous supplementation with 10 µM riboflavin or the cocktail of FMN and FAD (2.5 µM each) failed to rescue the growth defects observed in the knockdown strain (Supplementary Fig. 9A). However, it is noteworthy that only supplementation with an extraordinarily excessive amount of riboflavin (20 µM, well beyond physiological concentrations) successfully rescued the growth of the *ribH*-KD strain (Supplementary Fig. 9B). These findings highlight the intricate relationship between riboflavin supplementation and *M. tb* growth and underscore the importance of targeting riboflavin biosynthesis for anti-TB drug development.

Most of the anti-TB drugs such as rifampicin and isoniazid used to treat TB caused by *M. tb*, are primarily effective against actively replicating bacteria. However, their activity declines against non-replicating persister cells when tested in an in vitro dormancy model^37^. Since, earlier studies reported increased expression of RibH in oxidative stress and non-replicating persistent *M. tb*^38^, we exposed nutrient-starved non-replicating *M. tb* to RibH targeting drugs and provide evidence for interference with *M. tb* resuscitation.

Having established, *M. tb* RibH being a potential drug target, our study is anticipated to promote future research toward designing compounds with better fit, activity, and ADME properties. Given the limitations of rifampicin and isoniazid against non-replicating persister cells, and improved MIC of rifampicin by combination therapy observed in our checkerboard MABAs, combining multiple drugs with different mechanisms of action, could achieve effective clearance of both actively replicating bacteria as well as non-replicating persister cells, thereby improving the treatment outcomes. Future studies comparing and combining these newly discovered drugs with other anti-TB drugs, such as pyrazinamide, fluoroquinolones^39^ (e.g., moxifloxacin), or bedaquiline^40^ which have demonstrated better activity against NRP would reveal their synergy and interactions with drugs of the anti-TB regimen, other than rifampicin and isoniazid. As RibH protein and the drug binding sites are found to be highly conserved, the leads identified in this study have the potential to tackle a variety of geographically widespread *M. tb* lineages causing active, persistent, and reactivation TB^3^. The combination of these compounds with existing first-line anti-TB drugs also presents a potential strategy for overcoming drug resistance and enhancing treatment outcomes. Future preclinical studies are warranted to evaluate the full potential of these compounds and study their impact on TB control.

## Material and methods

### Ethics statement

All the in vitro studies involving risk groups 3 and 2 *M. tb* are done as per the guidelines and approval of the institutional biosafety committee.

### Bacterial strains and growth conditions

*M. tb* strain H37Rv was obtained from ATCC (ATCC 27294 strain). Frozen stocks were revived and cultured in Middlebrooks 7H9 broth (Himedia) supplemented with 10% oleic acid-albumin-dextrose-catalase (OADC) (Himedia), 0.4% glycerol (Himedia) and 0.05% Tween-80 (Sigma) in a BSL3 facility. For estimation of colony forming units, Middlebrooks 7H11 agar (Himedia) supplemented with 10% oleic acid-albumin-dextrose-catalase (OADC) (Himedia) and 0.5% glycerol (Himedia) was used.

The knockdown strain of *ribH* gene of *M.tb* used in this study was derived from mc^2^ 7902 (H37Rv Δ*panCD* Δ*leuCD* Δ*argB*)^41^ and was cultured in Middlebrook 7H9 broth or 7H11 agar supplemented with 10% oleic acid-albumin-dextrose-catalase (OADC) (Himedia) along with 0.5% glycerol, 0.05% tyloxapol and pantothenate, leucine and arginine (PLA) supplement containing l-pantothenate (24 mg/l); l-leucine (50 mg/l); and l-arginine (200 mg/l) to circumvent synthetic lethality^41^. The knockdown strains were selected on kanamycin (Kan) and hygromycin (Hyg) at concentrations of 25 μg/ml and 50 μg/ml, respectively as described^42^.

DH5-α and BL21 (DE3) strains of *E. coli* (NEB) were used for cloning and protein expression studies. *E. coli* strains were cultured in Luria Bertani (LB) broth (Himedia) or LB agar containing Kanamycin (Himedia) (Supplementary Table 3).

### Construction of knockdown strains

To achieve the repression of *ribH*, a pair of complementary oligonucleotides (Cr_UP: GATCTTTCCGTGCCAGCTG and Cr_DN: CGCAGCTGGCACGGAAAGATCCATG) specific to *Rv1416* ORF between 73 and 93 bp downstream to the 5' end, were synthesized, annealed and cloned in a hygromycin-resistant (listed in Supplementary Table 4), *E. coli-mycobacteria* shuttle plasmid pGrna2 at *Sph*I-*Acl*I sites, as previously described^42,43^, and illustrated in Fig. 1. The recombinant pGrna2 plasmid containing *Rv1416*-specific guide sequence was transformed into a kanamycin-resistant *M. tb* mc^2^ 7902::pTetint-dcas9^43^ to generate knockdown strain, namely, *ribH*-KD. Suppression was achieved by treatment of bacterial cultures with various concentrations of Anhydrotetracycline (ATc) in the range of 50–100 ng/ml for 4–9 days (unless indicated otherwise).

### In vitro growth analysis

Growth of *ribH*-KD strain of *M. tb* was monitored in 7H9 broth in the absence or the presence of 50 ng/ml and 100 ng/ml anhydrotetracycline (ATc) at 37 °C, 200 rpm. The growth was monitored by measuring optical density spectrophotometrically at a wavelength of 600 nm (OD_600nm_) for 9 days. The optical density was used to create time-stamped growth curves.

### RNA extraction and quantitative RT-PCR

The total RNA was extracted from the *M. tb* using Trizol reagent (Sigma) and purified using RNeasy Plus mini kit (Qiagen) as per the instructions given by the manufacturer. cDNA synthesis was done using the iScript cDNA synthesis kit (Bio-Rad). Then the qRT-PCR was performed with iTAq Universal SYBR Green Supermix (Bio-Rad), employing *ribH* gene-specific primers, and 15 ng cDNA. Gene expression was quantified using the qRT-PCR system (Roche LightCycler 480). The expression of *ribH* in the knockdown strain was compared to the parent strain (mc^2^ 7902) and *sigA* was used as an internal control for normalization. The primers used are listed in Supplementary Table 4.

### Computational details

In silico analyses were performed in Dell Precision T7610 workstation (8 processors; 8 GB RAM; ZOTAC 3 GB graphics; Maestro 9.8, Schrodinger, New York, U.S.A) workstation running on Redhat 6.1 Linux environment. Molecular docking calculations were conducted using two different strategies i.e., pharmacophore- based virtual screening (PBVS) and Structure-based flexible docking using Glide application in the Maestro 9.8 software package (Schrodinger, LLC, New York, 2015). The BITS *in-house* and Asinex compound libraries were first docked against *M. tb* RibH using PBVS approach by e-pharmacophore generation and validation leading to the identification of 15 molecules from Asinex library and 14 from BITS *in-house* library. We also performed blind docking on the BITS *in-house* library and short-listed an additional 26 molecules.

### Protein preparation

Crystal structure of *M. tb* lumazine synthase (encoded by *ribH*, *Rv1416*) with Protein Databank (PDB) ID 2C92 bound to 3-(1,3,7- trihydro-9-d-ribityl-2,6,8-purinetrione-7-yl) pentane 1 phosphate (TP6) having a resolution of 1.60 Å was considered for this study. There are other crystal structures published for this protein (with PDB IDs—1W19, 1W29, 2C94, 2C97, 2C9B, 2C9D, and 2VI5), however we have used 2C92 due to the best crystal structure resolution among all the published structures^19^. The protein exists in homopentameric form with substrate binding sites at the interface of two protein chains/subunits, hence for protein preparation and all the calculations, the complete dimer was used. Protein was prepared using the protein preparation wizard of Maestro 9.8, Schrodinger, New York, U.S.A. The co-crystal structure was pre-processed and water molecules within 5 Å distance from the ligand were removed, missing hydrogens and loops were added using Schrödinger protein preparation wizard. In addition, protein preparation also involved the addition of the bond orders and formal charges along with hydrogens to the hetero groups of the protein. Followed by an energy minimization to a convergence of RMSD 0.30 Å using OPLS_2003 as a force field.

### Ligand database screening

#### Compound libraries

For identifying lead compounds that could potentially inhibit the enzymatic activity of lumazine synthase and hence serve as an anti-mycobacterial agent, we utilized two libraries, the first library consists of ~ 3000 compounds from our BITS (Birla Institute of Technology and Science) *in-house* database containing compounds that have ADME properties commonly observed in drug and drug-like compounds^36^ and ASINEX compound library comprising of ~ 0.57 million compounds (https://www.asinex.com/screening-libraries-(all-libraries)).

#### Hypothesis generation for energy-optimized structure-based e-pharmacophore

 Glide energy grids were generated for the prepared protein–ligand complex. The binding site of RibH was defined by a rectangular box surrounding the ligand in the X-ray structure. The ligand was refined using Glide and default settings were used for the refinement and scoring. Starting with the refined ligand, pharmacophore sites were automatically generated with Phase with the default set of six chemical features: hydrogen bond acceptor (A), hydrogen bond donor (D), hydrophobic (H), negative ionizable (N), positive ionizable (P), and aromatic ring (R). Hydrogen bond acceptor sites were represented as vectors along the hydrogen bond axis in accordance with the hybridization of the acceptor atom. Hydrogen bond donors were represented as projected points, located at the corresponding hydrogen bond acceptor positions in the binding site. Projected points allow the possibility for structurally dissimilar active compounds to form hydrogen bonds in the same location, regardless of their point of origin and directionality. Each pharmacophore feature site is first assigned an energetic value equal to the sum of the Glide XP contributions of the atoms comprising the site. This allows sites to be quantified and ranked based on these energetic terms.

#### Database preparation

The commercial chemical ASINEX virtual library with over 600,000 compounds & *in-house* 3000 compounds library were processed to avoid redundancy and Lipinski filters were applied to select compounds that have drug-like properties. The purpose of the redundancy check is to avoid structures with the same SMILES notation (Singleline entry system for structures) and also to remove conformers of very similar conformation using an RMSD cutoff of 1.0 Å. Database molecules were prepared using LigPrep and Epik to expand protonation and tautomeric states at pH 7.0 (± 2.0 pH units). For each ligand, LigPrep generates a maximum of eight tautomeric forms. If for a compound, chirality is already specified, it is retained. However, if the compound chirality is undefined, at most 32 stereoisomers were generated for each ligand used for screening. Conformational sampling was performed for all database molecules using the ConfGen search algorithm. We employed ConfGen with the OPLS_2005 force field and a duplicate pose elimination criterion of 1.0 Å RMSD to remove redundant conformers. A distance-dependent dielectric solvation treatment was used to screen electrostatic interactions. A maximum relative energy difference of 10.0 kcal/mol was chosen to exclude high-energy structures. Using Phase, the database was indexed with the automatic creation of pharmacophore sites for each conformer to allow rapid database alignments and screening.

#### E-pharmacophore database screening

To dock the compound libraries into the generated grid, the libraries were first pre-processed to create an e-pharmacophore. E-pharmacophore is defined as a hypothetical ideal 3D orientation or spatial and electronic characteristics of the functional groups of a molecule necessary to ensure optimal interactions with a specific target protein to achieve a biological response. For the e-pharmacophore approach, explicit matching was required for the most energetically favorable site, with a scoring cut-off better than −1.0 kcal/mol. Screening molecules were required to match a minimum of four sites. Distance matching tolerance was set to 2.0 Å as a balance between stringent and loose-fitting matching alignment. Database hits were ranked in the order of their Fitness score as implemented in the default database screening in Phase. We validated the pharmacophore by docking the co-crystal ligand TP6. E-pharmacophore generation involves two steps, in the first step based on the co-crystal structure of the protein with a ligand (TP6), e-pharmacophore features such as hydrogen bond acceptor (A), hydrogen bond donor (D), hydrophobic (H), negative ionizable (N), positive ionizable (P) and aromatic ring (R) were assigned to the bound ligand using the Glide tool.

#### Molecular docking

Database ligands were docked into the binding sites of RibH with Glide utilizing the high-throughput virtual screening (HTVS) scoring function to estimate protein–ligand binding affinities. The centre of the Glide grid was defined by the position of the co-crystallized ligand. Default settings were used for both grid generation and docking. Compounds with best docking and GLIDE scores were then subjected to GLIDE XP screening. Docked protein and ligand datasets were visualized and molecular surface complex pictures were generated using Maestro. Using this tool, we identified 17 compounds from ASINEX.

### Structure-based design by virtual screening:

#### Grid generation

The receptor site or the active site at the interface of two subunits, where the inhibitors are to be docked was generated using the ‘generate grid’ sub- application of the Glide tool in Maestro 9.8. For the generation of the receptor grid, active site residues were selected to locate the coordinates of the receptor center. The grid has default parameters of van der Waals scaling factor of 1.0 and charge cut off of 0.25 subject to an OPLS_2003 force field. The generated grid was then utilized as an active site for docking [*in-house* database of 3000 compounds] using the ‘extra precision’ (XP) flexible docking method located in the Glide tool. Using this tool, we further identified 26 compounds.

For evaluating and ranking the shortlisted compounds, we utilized two-step method. First, a docking score cut-off of −6 was applied and any molecule from the libraries with a docking score > −6 (Between 0 to −6) were excluded. Next, the shortlisted compounds were further evaluated on the basis of their molecular interactions with the important amino acids (29 amino acids) in the binding site of RibH. These amino acids were selected based on the co-crystal ligand interactions. Hence, a docking score < −6 plus and interaction with at least 3 amino acids were further shortlisted assuming a higher probability of binding to the substrate binding site.

### Determination of minimal inhibitory concentration (MIC) of shortlisted compounds against *M. tb* by microplate Alamar Blue assay (MABA)

MIC for the new compounds identified through in silico analysis was carried out using methods as described^44^. Briefly, *M. tb* strain H37Rv (ATCC27294 strain) was cultured in Middlebrooks 7H9 broth (Himedia) supplemented with 10% OADC (Himedia) and 0.05% Tween-80 (Sigma) for 4–5 days. The culture was grown to the mid-log phase until an OD_600nm_ of 0.4–0.8. The culture was then diluted to obtain an OD_600nm_ of 0.01 corresponding to ~ 10^6^ CFU/ml.

Based on the docking score and chemical structure 40 compounds were selected from BITS *in-house* library and 15 compounds from the ASINEX compound library (Supplementary Table 1). These compounds along with standard anti-TB drugs—rifampicin (Himedia) and isoniazid (Sigma) were dissolved in absolute DMSO at a concentration of 2 mg/ml to prepare the drug stock. The drug stock was then serially diluted in a 96-well plate to achieve a final concentration in the range of 50 µg/ml to 0.006 µg/ml. To each well containing the desired concentration of compounds or standard anti-TB drugs, 0.1 ml of the bacterial culture (~ 10^5^ CFU/well) was added in a final volume of 200 µl. The plate was then sealed and incubated at 37 °C for 6 days. For the Alamar Blue assay, a mixture of resazurin sodium salt (Sigma) and Tween-80 was freshly prepared in autoclaved single distilled water to obtain a final concentration of 1.27 mM and 8%, respectively and the solution was then filter sterilized using 0.2 µ syringe filter. On day 6, 30 µl of this freshly prepared sterile solution is added to each well of the plate and incubated for 16–18 h at 37 °C in the dark. Color change from purple to pink is noted and photographed, wherein, purple color indicates a lack of growth and pink indicates live bacteria. MIC is determined from the minimal concentration of the antibiotics or compounds exhibiting purple color. MIC shown in Supplementary Table 1 solely reflects the data w.r.t. primary screening of the compounds in a single run. Based on this primary round, compounds with MIC ranging between 6.25 and 0.78 µg/ml were shortlisted for secondary and tertiary screening. Further shortlisting was done from these rounds, to select the top 3 compounds with MIC ranging between 1.56 and 0.78 µg/ml. Since only three shortlisted hits consistently showed potent antimycobacterial activity, they were then tested more rigorously using a variety of methods discussed in the main body of the manuscript.

The three most potent compounds identified from the above MABA screen were then tested in combination with the first-line anti-TB drugs. For this experiment, MABA was performed with minor modifications. Briefly, for each of the three potent compounds, 3–4 concentrations were typically tested, (a) at MIC (b) at two concentrations below, and (c) at one concentration above MIC in the range of 3.125 µg/ml to 0.19 µg/ml (two-fold serial dilution). The standard anti-TB drugs were tested in the range of 0.19 µg/ml to 0.006 µg/ml (two-fold serial dilution) for rifampicin and 0.39 µg/ml to 0.012 µg/ml (two-fold serial dilution) for isoniazid. To achieve this, 100 µl of first-line anti-TB drug at 2× of desired concentrations were added to the wells, followed by the addition of 50 µl of 4× concentration of the short-listed compounds. Following the addition of compounds, bacterial culture was diluted to obtain an OD_600nm_ of 0.02, and 50 µl was added to each well to obtain a final volume of 200 µl/well containing ~ 10^5^ CFU/well. The plate was then sealed and incubated at 37 °C for 6 days, followed by Alamar Blue addition and analysis of results as described above. The fractional inhibitory concentration index (FICI) was calculated to determine the synergistic effect of these compounds with rifampicin and isoniazid as described^45^. The fractional inhibitory concentration for each compound was calculated as:

$${\rm Fractional\, inhibitory\, concentration\, of \,compound\, A: FIC_{A}}=({\rm MIC \,of\, compound\, A \,in\, the\, presence\, of \,compound\, B)}/ ({\rm MIC\, of\, compound\, A \,alone);}$$

$${\rm Fractional\, inhibitory\, concentration\, of \,compound\, B:\,\, FIC_{B}}=({\rm MIC \,of\, compound\, B\,in\, the\, presence\, of \,compound\, A}/ ({\rm MIC\, of\, compound\, B \,alone);}$$

FICI was calculated as FIC_A_ + FIC_B._ Synergism is defined as FICI ≤ 0.5, antagonism as FICI ≥ 4 and no interaction if the FICI values range from 0.5–4.

### Determination of cytotoxicity of shortlisted compounds by MTT assay

To determine the cytotoxicity of the shortlisted compounds, 3-(4,5-dimethylthiazol-2-yl)-2,5-diphenyltetrazolium bromide (MTT) cytotoxicity assay was performed as per manufacturer’s instructions. This assay allows the measurement of cellular metabolic activity as an indicator of cell viability, proliferation, and cytotoxicity. Briefly, HEK293t cell line (human embryonic kidney cell line) was maintained in high glucose Dulbecco’s Modified Eagle’s Medium (DMEM) with 4.5 g/l glucose, 2 mM l-glutamine, 25 mM HEPES buffer, and 3.7 g/l sodium bicarbonate (Himedia) supplemented with 10% fetal bovine serum and 1× Pen Strep (penicillin and streptomycin solution) at 37 °C and 5% CO_2._ In a 96-well plate, 2 × 10^4^ cells were seeded and allowed to get adhered to the dish for 12 h. Following, adherence, all the media was replaced with 100 µl of fresh media (without antibiotic). In a separate 96-well plate, the three shortlisted compounds were twofold serially diluted in complete DMEM (without antibiotics). Diluted compounds were then added to the cells at a final concentration of 50 µg/ml to 0.78 µg/ml. The plate was then incubated for 48 h at 37 °C and 5% CO_2_. On Day 4, a stock solution of MTT (Sigma) was freshly prepared in sterile 1× PBS (Himedia) at a concentration of 20 mg/ml. MTT was further diluted to 5 mg/ml in complete DMEM (without antibiotics), followed by the addition of 20 µl of this diluted MTT into each well and incubated for 4 h. After 4 h, the media was completely removed and 100 µl of absolute DMSO was added to dissolve the insoluble formazan crystals formed by the action of mitochondrial dehydrogenase of live cells on soluble MTT (yellow). After two hours of the dissolution of formazan crystals, purple coloured solution was analysed by taking absorbance using a microplate (ELISA) reader at 570 nm and 620 nm using M4 SpectraMax^®^ (Molecular Devices LLC). Deeper the purple color, the greater is the cell viability. Percentage cytotoxicity was calculated as described previously, using the following formula:

$$\% Cytotoxicity =100-\left[\frac{\{drug\, treated -A_{570\, nm}\, media \, control\}}{\{A_{570\, nm}\, untreated \, cells-A_{570\, nm}\, media\, control\} }\right]$$

$$\% Cytotoxicity =100-\left[\frac{\{drug\, treated -A_{570\, nm}\, media\, control\}}{\{A_{570\, nm}\, untreated \, cells-A_{570\, nm}\, media\, control\} }\right] \times 100.$$

### Determination of intracellular anti-mycobacterial activity of the shortlisted compounds in the THP-1-derived macrophages

Shortlisted compounds were tested for their intracellular anti-mycobacterial activity by methods as described previously^46^ with minor modification. Briefly, the THP-1 cell line (human monocytic leukaemia cell line) was maintained in RPMI 1640 with 2 mM l-glutamine, 25 mM HEPES buffer, and 2 g/l sodium bicarbonate (Himedia) supplemented with 10% fetal bovine serum and 1× Pen Strep (Penicillin and streptomycin solution) at 37 °C and 5% CO_2._ In a 48-well plate (Thermo Scientific™ Nunc), 3 × 10^4^ THP-1 cells were seeded and differentiated by the addition of 5 ng/ml PMA (Sigma) in complete RPMI media (Himedia) (with 1× Pen Strep) as described above. Differentiated macrophages were then infected with *M. tb.* (H37Rv) at a multiplicity of infection (MOI) of 1:5 (1 macrophage: 5 bacteria) for 4 h^47^.

Extracellular non-phagocytosed bacteria were removed by washing the infected cells three times with warm DPBS. For day 0 count, we lysed the infected macrophages (n = 3) to determine the CFU by plating various dilutions of the lysates on 7H11 agar and incubated at 37 °C for 3–4 weeks followed by bacterial enumeration and calculations. At this point we observed ~ 10,000 CFU/ml (per well). This reveals an actual MOI of 1: 0.33 equating to ~ 3 M*. tb* per 10 macrophage cells. For calculating the impact of drugs on growth inhibition of *M. tb* in macrophages, we first infected the macrophages in similar fashion as described above. Following removal of extracellular bacteria post 4 h infection, the shortlisted compounds or first-line anti-TB drugs were prepared as stock containing 5× of the desired concentration in incomplete RPMI media (without FBS and without Pen Strep). For treatment, DPBS was removed and 400 µl of incomplete media (without FBS and without Pen strep) was added to each well, followed by the addition of 100 µl of first-line anti-TB drug or short-listed compounds at 5× of the desired concentrations, to obtain a final volume of 500 µl/well. Plates were then incubated at 37 °C and 5% CO_2_ for 5 days. On day 6, we collected supernatant and mixed with the macrophages lysed with lysis buffer (sterile 0.025% SDS), followed by centrifugation to pellet down all the bacilli. Following this step, bacteria is then plated on to 7H11 agar plates. The plates were incubated at 37 °C for 3–4 weeks and colonies were counted for CFU determination.

### Determination of anti-mycobacterial activity of drugs using nutrient starvation model of dormancy by most probable number (MPN) method

Shortlisted compounds were tested for their anti-mycobacterial activity employing nutrient starvation dormancy model using MPN assay as described previously^48^ and in Supplementary Fig. 5. To mimic the dormant conditions, *M. tb* H37Rv was first grown in nutrient rich (7H9 supplemented with OADC and 0.05% tween 80) media for 7 days until an OD_600nm_ of 0.4–0.8. On day 8, the bacterial culture was centrifuged, washed twice with sterile PBS (Himedia) and then re-suspended in 10 mL PBS in sealed tubes. The sealed tubes were incubated at 37 °C with 5% CO_2_ in humid and stationary condition (without shaking) for 6 weeks. Following 6 weeks of starvation, 200 µl of nutrient-starved cultures were taken in a microfuge-tubes and treated with 10 µM of the first-line TB drugs, rifampicin and isoniazid and three of our shortlisted compounds for 7 days at 37 °C with 5% CO_2_ in humid and stationary condition (without shaking). Untreated starved cells were similarly incubated as a control group. Post 7 weeks of starvation (including 1 week of drug treatment) cells were ten-fold serially diluted (10 to10^6^) in complete 7H9 media in microfuge tubes. From each serial dilution, 50 µl of diluted cultures were plated into a 48-well plate in triplicates in a 450 µl of 7H9 (complete) making a total volume of 500 µl/well. The plates were then incubated at 37 °C with 5% CO_2_ in humid and stationary condition for 2–3 weeks (until bacterial growth is observed in most diluted untreated culture). Following this, MPN of bacterial cells was calculated. Briefly, as described in Supplementary Fig. 5, bacterial growth in three consecutive dilutions (10^–5^, 10^–6^ and 10^–7^) was noted. *M. tb* viability was calculated as mean MPN/ml. The data is represented as the mean of n = 3 biological replicates with 95% confidence interval as per the three-tube MPN table^48^. The MPN methods have recently been applied for preclinical evaluation of anti-TB drugs using mouse tissues as well^49^.

### Statistical analysis

Data was analysed using Microsoft excel, GraphPad Prism Software Version 5.0 using Student’s t-test and one-way ANOVA, wherever applicable.

### Supplementary Information

Supplementary Information.

## Data Availability

All data that supports the findings of this study are available from corresponding author upon reasonable request.
