# Supplementary material for: Discovery of potent antimycobacterial agents targeting lumazine synthase (RibH) of Mycobacterium tuberculosis
Source: Sci Rep. 2024 May 28;14:12170. doi: 10.1038/s41598-024-63051-6 (PMC11133327; doi:10.1038/s41598-024-63051-6)
Supplement: Supplementary file 1 — Supplementary Information. [file 41598_2024_63051_MOESM1_ESM.docx]

**Supplementary Material**

**Discovery of potent antimycobacterial agents targeting lumazine synthase (RibH) of Mycobacterium tuberculosis**

Monica Singh^a^, Anannya Dhanwal^a^, Arpita Verma^a^, Linus Augustin^b^, Niti Kumari^c^, Soumyananda Chakraborti^a,d^, Nisheeth Agarwal^b^, Dharmarajan Sriram^e^, Ruchi Jain Dey^a^*

^a^Department of Biological Sciences, Birla Institute of Technology and Science Pilani, Hyderabad Campus, Shameerpet, Hyderabad-500078, Telangana State, India

^b^ Translational Health Science and Technology Institute, Faridabad-121001, Haryana, India

^c^National Institute of Animal Biotechnology (NIAB), Hyderabad-500032, Telangana State, India

^d^National Institute of Malaria Research, Indian Council of Medical Research (ICMR), New Delhi- 110077, India

^e^Department of Pharmacy, Birla Institute of Technology and Science Pilani, Hyderabad Campus, Hyderabad-500078, Telangana State, India

*, corresponding author

Email for correspondence: ruchij80@hyderabad.bits-pilani.ac.in

**Supplementary Figure 1- 9**

**Supplementary Tables 1- 8**

**Supplementary Methods**


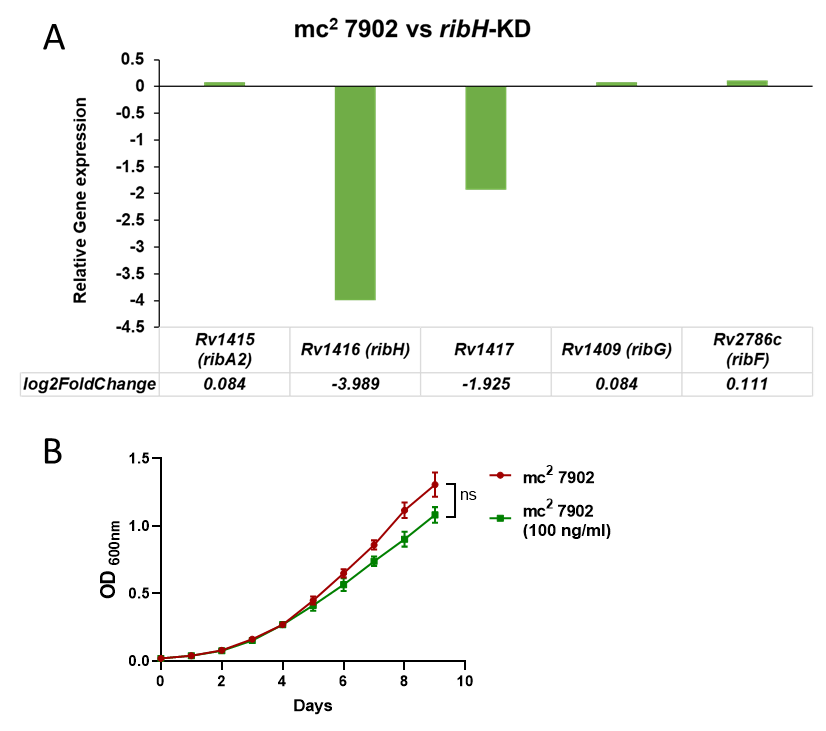


**Supplementary Figure 1. Target specific CRISPRi mediated downregulation of RibH.** A. Gene expression analysis of *Rv1416* (*ribH)* and other genes of the pathway namely, *Rv1415* (*ribA2*), *Rv1417* (co-transcribed with *ribH*), *Rv1409* (*ribG*), and *Rv2786c* (*ribF*) were carried out to determine the target specificity of *ribH* knock down using CRISPRi. It is evident that downregulation of *ribH* by the gRNA designed in the study is specific to *ribH* gene and there is no off-target effect of guide on other gene loci. B. Growth characteristics of *M. tb* mc^2^ 7902 parent strain in presence and absence of 100 ng/ml ATc. Further to test if the diminished growth observed in case of ribH-KD (Figure 1) is specific effect of knockdown induced by ATc on riboflavin biosynthesis in a gene specific manner, we compared the growth of parent strain in absence and presence of ATc. Unlike the knockdown strain, the parent strain did not show any significant change in growth. Figure shows growth curve of the parent culture after 9 days of ATc treatment. Statistical analysis was performed using One-way ANOVA, ns- non-significant on GraphPad Prism 8.

**
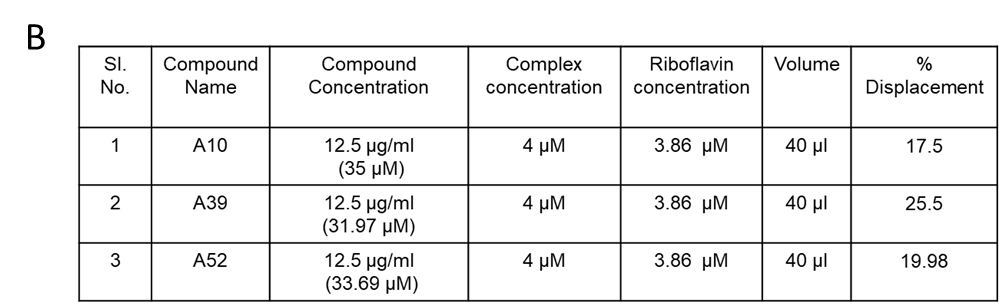
**

**Supplementary Figure 2.** **Riboflavin displacement assay**: The table depicts the percentage of riboflavin displaced from the active site of RibH (complexed with equimolar riboflavin) on addition of 12.5 µg/ml each of A10, A39 and A52 compounds. This assay is based on the fluorescence intensity of free riboflavin. Compounds competing for the same binding site as that of riboflavin in RibH protein causes displacement of the riboflavin, leading to increase in fluorescence. % displacement is calculated and data is represented in the table form, where n = 8. Greater the % displacement, greater the extent of binding of the compounds at the active site. For details see supplementary methods.

**
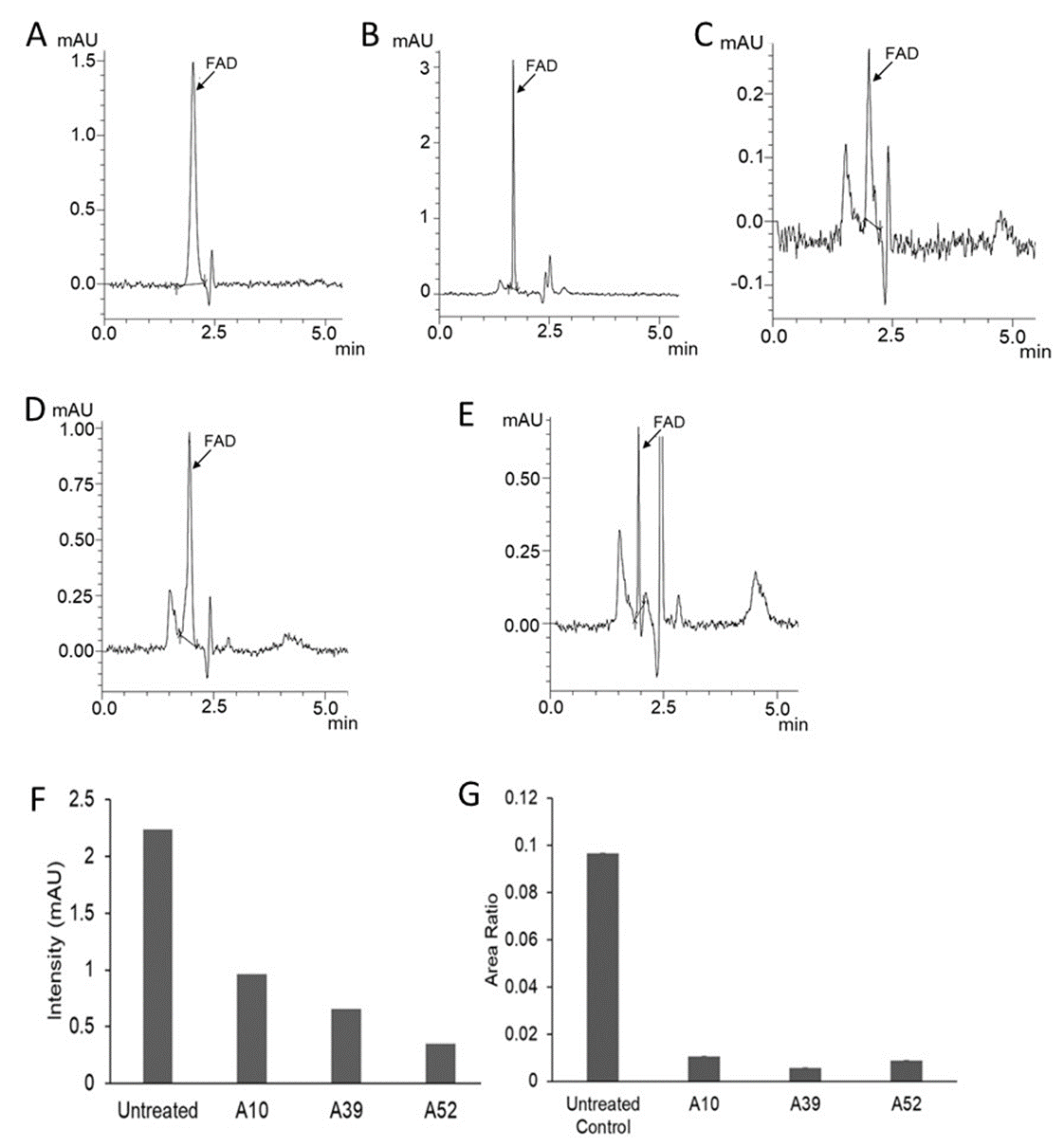
**

**Supplementary Figure 3. HPLC Chromatograms for FAD analysis. (A-E)** Figure depicts FAD^+^ peak intensity in milli absorbance units (mAU) observed on HPLC analysis of A. standard FAD^+^ (1 μM), B. nucleotides extracted from untreated *M. tb* H37Rv, C. nucleotides extracted from *M. tb* H37Rv treated with A10, D. nucleotides extracted from *M. tb* H37Rv treated with A39 E. nucleotides extracted from *M. tb* H37Rv treated with A52. FAD peak is detected at retention time of ~1.9 minutes; X-axis represent time scale in min and Y axis represent mAU. F. Comparative analysis of FAD^+^ peak intensity in mAU (Y axis) among various groups (X-axis) derived from data obtained from chromatograms shown in A-E. G**.** Relative peak area ratio (mean±s.d) of FAD^+^ comparing area under curve (AUC) normalized with respect to internal standard cyclic xanthine monophosphate (cXMP), a synthetic unnatural nucleotide added to each sample during nucleotide extraction procedure as described in supplementary methods. AUC is first normalized with respect to cXMP and then the area ratios are compared among various untreated and drug-treated groups.


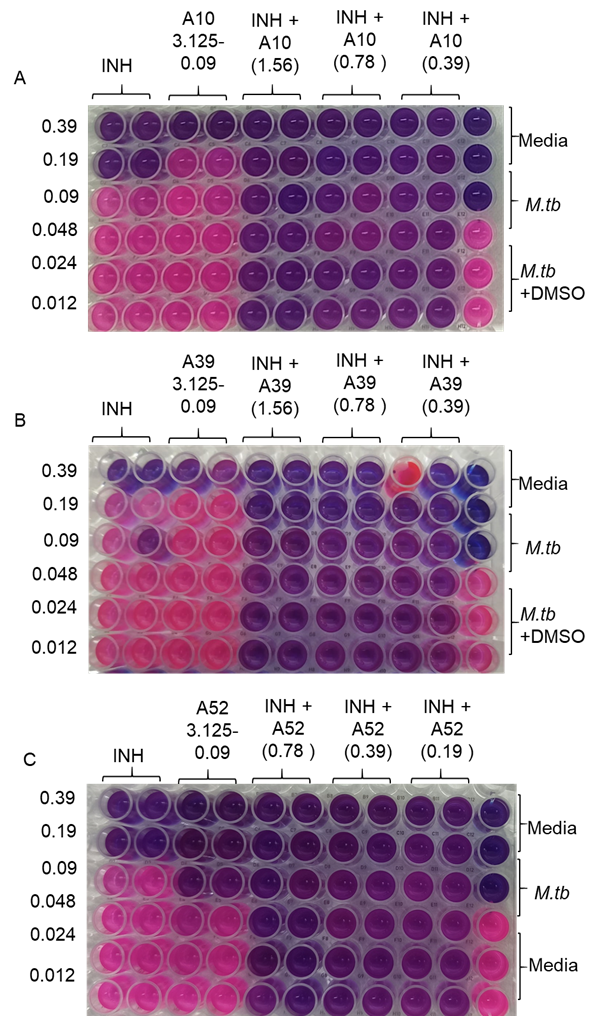


**Supplementary Figure 4. Anti-mycobacterial activity of short-listed compounds in combination with first line anti-TB drug isoniazid.** The figure depicts the representative pictograms obtained on screening of anti-mycobacterial activity of three of the shortlisted compounds against *M. tb* H37Rv in combination with isoniazid by microplate Alamar blue assay (MABA)*.* The assay was performed twice with two technical replicates in each case. In each case (A, B and C) isoniazid was tested at various concentrations between 0.39 µg/ml to 0.012 µg/ml (two-fold serial dilution) either alone or in combination with various concentrations of A. A10, B. A39 and C. A52. These shortlisted compounds were either tested alone at various concentrations between 3.125-0.09 µg/ml (two-fold serial dilution) or at their respective MICs and sub-MIC concentrations in combination with various concentrations of isoniazid (MIC to sub-MIC range). A synergistic enhancement in antimycobacterial activity of isoniazid and shortlisted compounds was observed, as described in Table 2.

**Supplementary Figure 5. Nutrient starvation (an *in vitro* dormancy/non-replicating persistence) model and MPN assay to test various anti-TB drugs.** To mimic the dormant conditions *in vitro*, *M. tb* H37Rv is first grown in nutrient-rich (7H9 supplemented with OADC and 0.05% tween® 80) media for 7 days until an OD_600nm_ of 0.4-0.8. On day 8, the bacterial culture is centrifuged, washed with sterile phosphate-buffered saline (PBS), and then re-suspended in PBS in sealed tubes. The sealed tubes are incubated at 37°C with 5% CO_2_ in humid and stationary conditions for six weeks. Following 6 weeks of starvation, 200 µl of nutrient-starved cultures are taken in a microfuge tube and treated with drugs for 7 days. Untreated starved cells are similarly incubated as the control group. Post 7 weeks of starvation (including 1 week of drug treatment) cells are then ten-fold serially diluted (10 to 10^-6^) in complete 7H9 media in microfuge tubes. From each serial dilution, 50µl of diluted cultures are plated into a 48-well microtiter plate (flat-bottomed) in triplicates in 450 µl of 7H9 media (complete) making a total volume of 500 µl/well. The plates are then incubated at 37°C with 5% CO_2_ in humid and stationary condition for 2-3 weeks (until bacterial growth is observed in the most diluted untreated culture). Following this, MPN of bacterial cells is calculated. Bacterial growth in three consecutive dilutions (10^-5^, 10^-6^, and 10^-7^) is noted*. M. tb* viability is calculated as mean MPN/ml. The CFU data (n = 3) is represented as the geometric mean (with 95% CI) as per the three-tube MPN table (See Figure 8 for data).


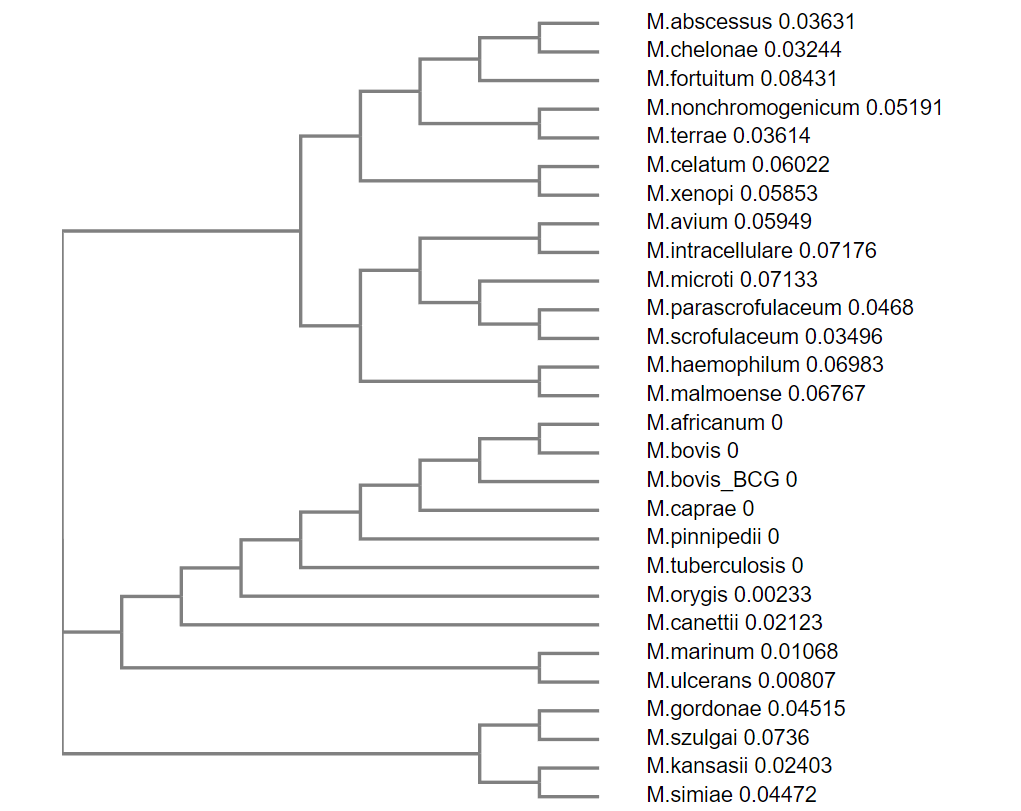


**Supplementary Figure 6. Phylogenetic analysis of RibH protein sequence across various mycobacterial species belonging to *M. tb* complex and non-tuberculous mycobacteria.** The phylogenetic tree was constructed for the RibH protein sequences of various mycobacterial species using T-Coffee multiple sequence alignment server (https://tcoffee.crg.eu/). The scores mentioned in the tree are calculated by first calculating pairwise identity between two sequences. The identity between two sequences is then converted to a measure of evolutionary distance.


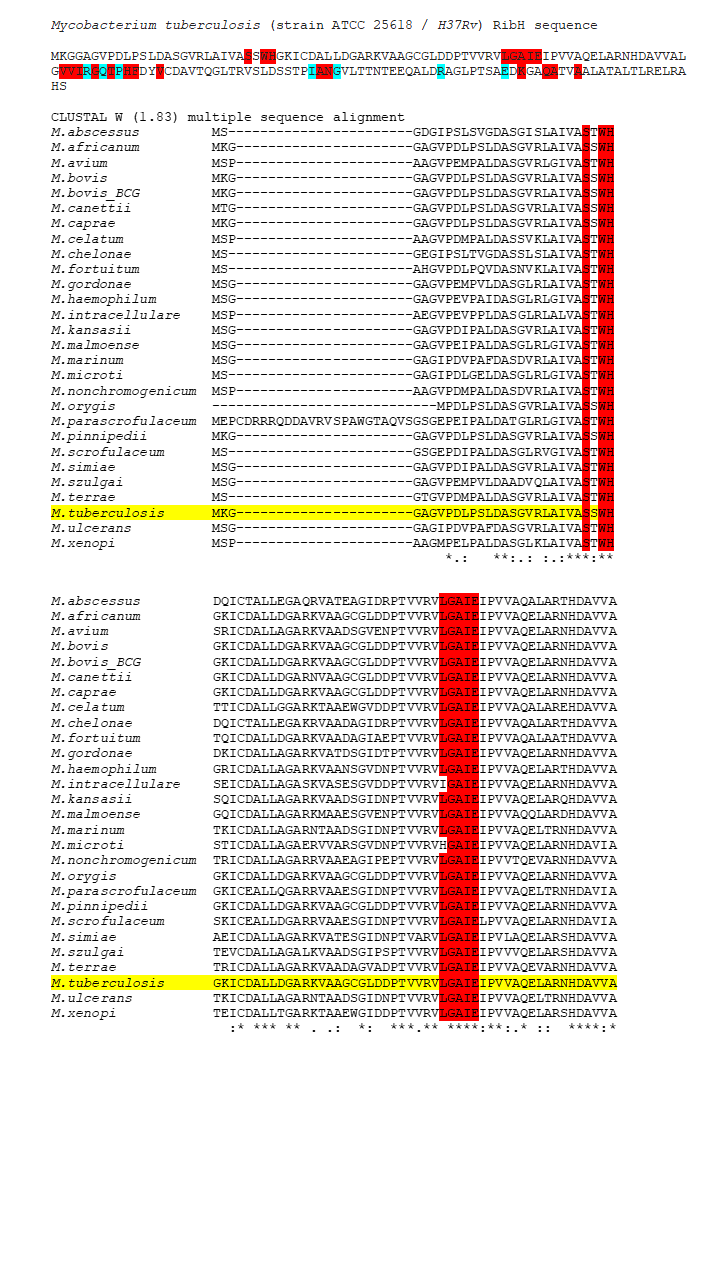


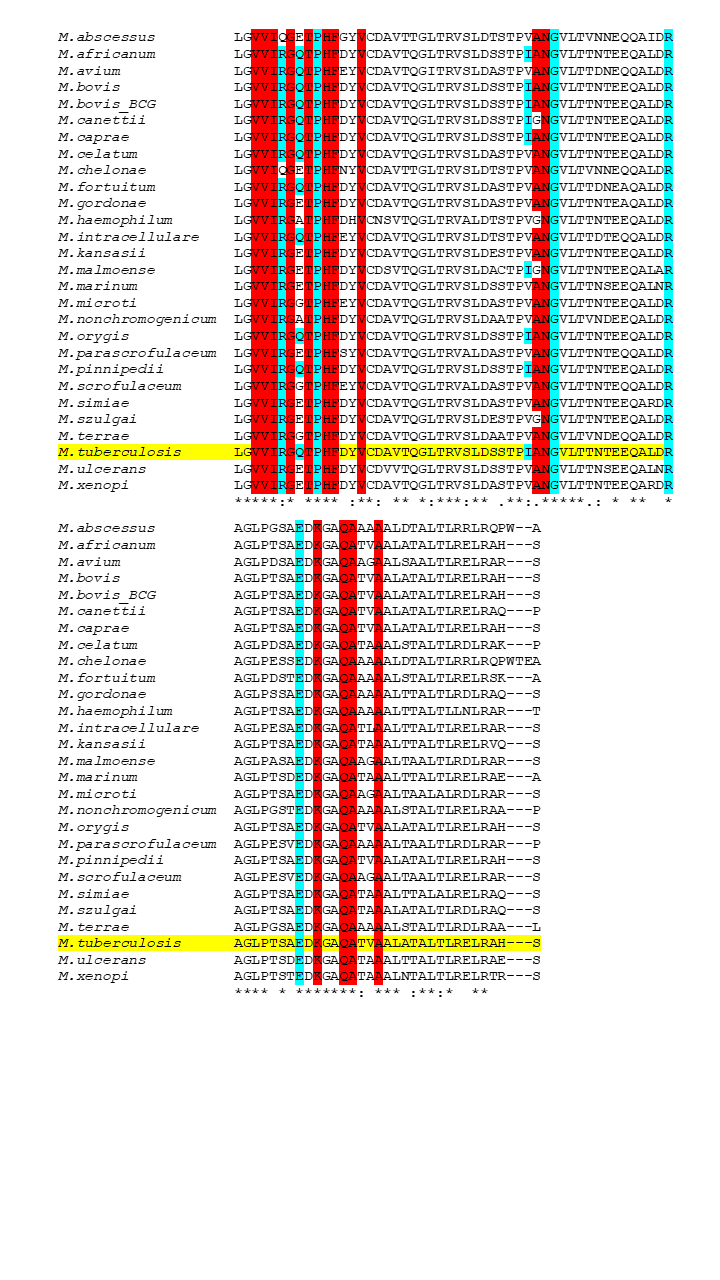


**Supplementary Figure 7**. Sequence alignment of lumazine synthases from different mycobacterial species against the sequence of *M. tb* (strain ATCC 25618 / H37Rv). The sequence highlighted in yellow is that of RibH from *Mycobacterium tuberculosis* (strain ATCC 25618 /H37Rv). The residues highlighted in red indicated the ones present in the drug binding pocket. The residues highlighted in blue indicate the additional residues (along with the residues highlighted in red) present in the co-crystal ligand binding pocket. Multiple sequence alignment was performed using T-Coffee multiple sequence alignment server.

**
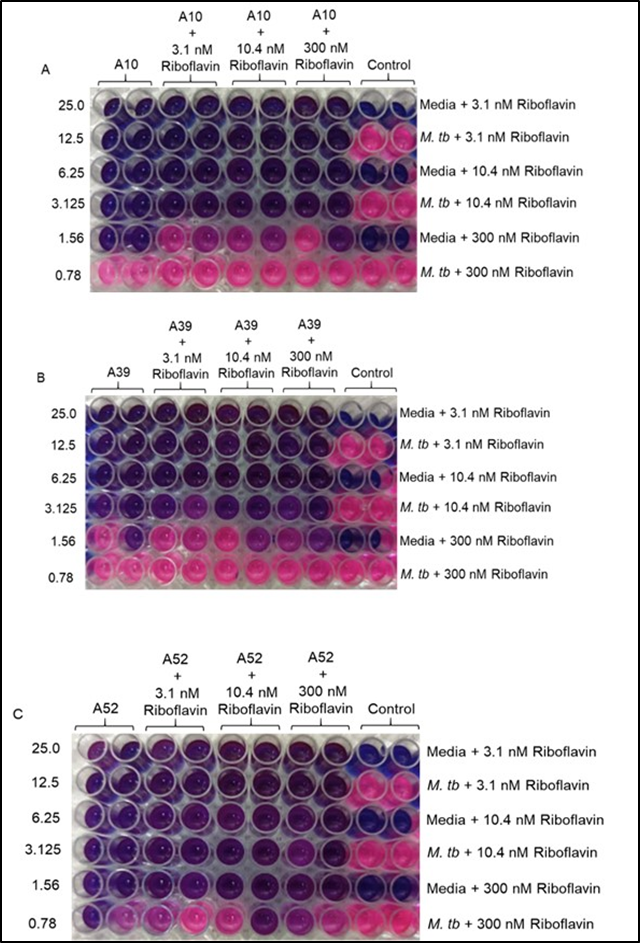
**

**Supplementary Figure 8.** Anti-mycobacterial activity of short-listed compounds in combination with various concentrations of riboflavin. The figure depicts the representative pictograms obtained on screening of anti-mycobacterial activity of three of the shortlisted compounds against *M. tb* H37Rv in combination with riboflavin. Riboflavin was tested at three different concentrations (3.1 nM, 10.4 nM and 300 nM) based on the range of plasma riboflavin concentrations in humans. In our assay conditions, riboflavin was tested in combination with various concentrations of A10, A39 and A52. A. A10 was tested alone at various concentrations between 25-0.78 µg/ml (two-fold serial dilution) and in combination with various concentrations of riboflavin (3.1 nM, 10.4 nM and 300 nM). In combination with riboflavin A10 shows an MIC of 3.125-1.56 µg/ml at all the three riboflavin concentrations tested in the assay. B. A39 was tested alone at various concentrations between 25-0.78 µg/ml (two-fold serial dilution) and in combination with various concentrations of riboflavin (3.1 nM, 10.4 nM and 300 nM). In combination with riboflavin A39 shows an MIC of 3.125 µg/ml at all riboflavin concentrations C. A52 was tested alone at various concentrations between 25-0.78 µg/ml (two-fold serial dilution) and in combination with various concentrations of riboflavin (3.1 nM, 10.4 nM and 300 nM). In combination with riboflavin A52 shows an MIC of 1.56-0.78 µg/ml at all the riboflavin concentrations.


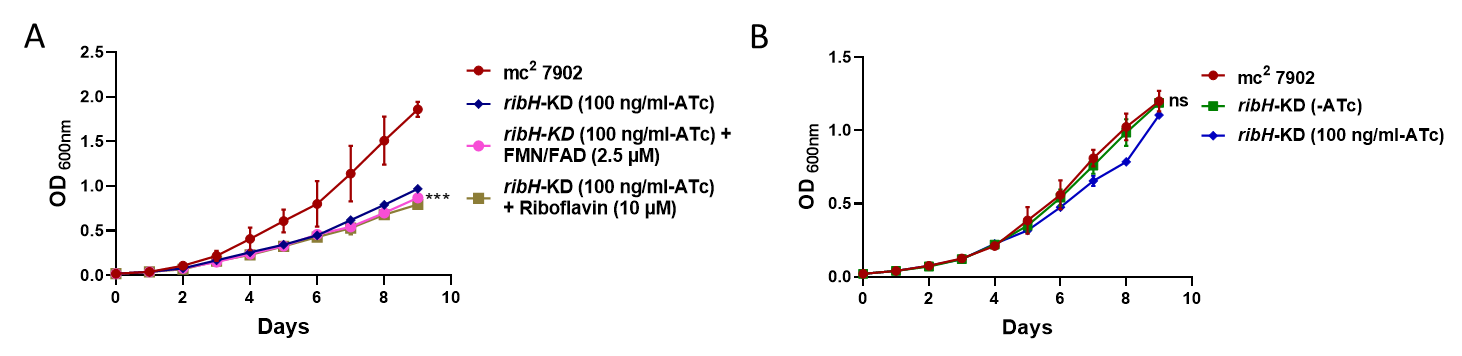


**Supplementary Figure 9.** Growth of parent mc^2^ 7902 and *ribH*-KD in chemically defined synthetic media with (A) riboflavin [10 μM or cocktail of FMN and FAD (2.5 μM each)] B. riboflavin (20 μM), in absence and presence of 100 ng/ml ATc. OD_600nm_ was recorded at regular intervals up to 9 days. Each graph is plotted with the data obtained from two independent experiments.

**Supplementary Tables**

**Supplementary Table 1. *In silico* molecular docking of various compounds against RibH.** The table depicts the docking scores, structure of the compounds, minimum inhibitory concentration (MIC) against *M. tb* H37Rv, and important amino acids involved in interaction with the concerned drug molecules.

| **Sl.**  **No.** | **Compound**  **Code** | **Structure** | **Docking**  **Score** | **MIC**  **(µg/mL)** | **Amino acid residues**  **interacting with ligand** |
| --- | --- | --- | --- | --- | --- |
| 1 | TP6 |  |  | Not applicable | SER 25, TRP 27, HIE 28, ILE 60, GLU 61, VAL 81, ILE 83, GLN 86, THR 87 |
| 2 | Riboflavin^#^ | 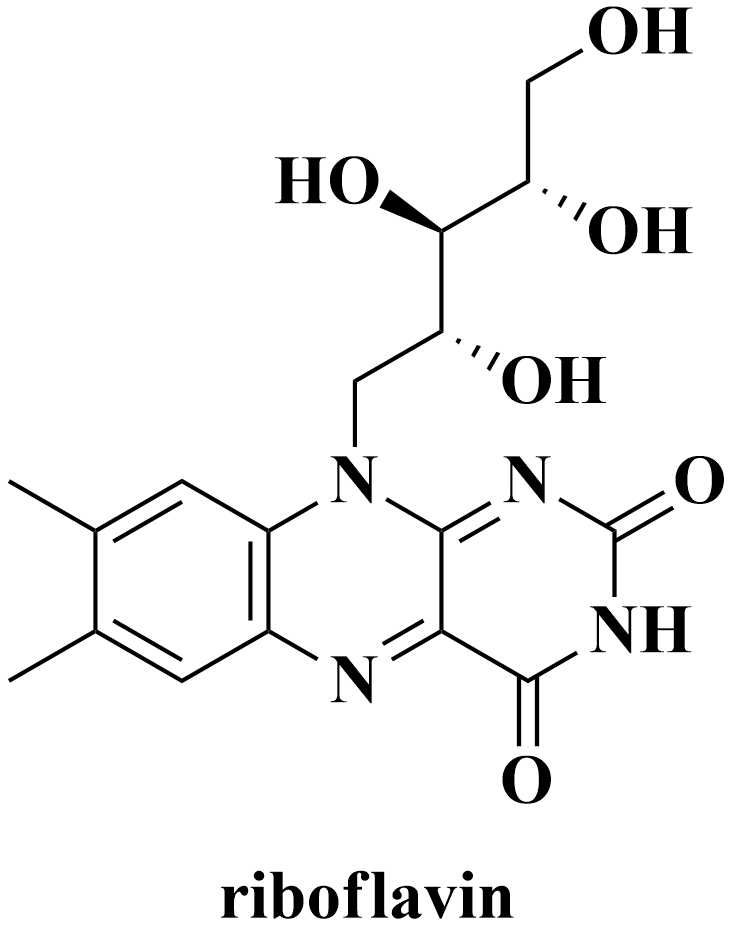 | -12.528 | Not applicable | HIS 28, ALA 59, GLU 61, VAL 81, ILE 83, ASN 114, LYS 138 |
| 3 | NR 353 (A52) ^#^ | 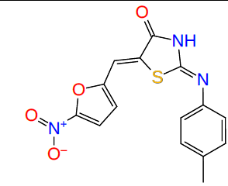 | -8.431 | 0.78 | SER 25, ALA 59, GLU 61, VAL 81, ASN 114 |
| 4 | NR-310 (A10) ^#^ | 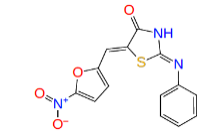 | -7.276 | 1.56 | ALA 59, GLU 61, VAL 81 |
| 5 | NR-340 (A39) ^#^ | 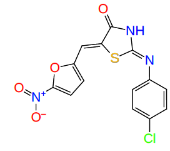 | -6.825 | 1.56 | ALA 59, GLU 61, VAL 81, THR 87, ASN 114 |
| 6 | 9F-Srihari*^,#^ | 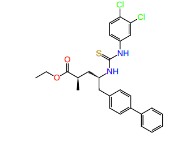 | -7.725*  (-6.652^#^) | 3.125 | HIS 28, THR 87, HIE 89, ARG 128, LYS 138 |
| 7 | QNH-08*^,#^ | 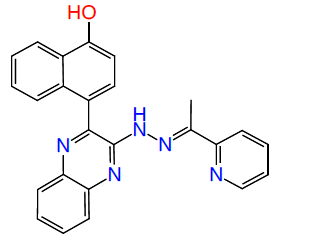 | -7.722*  (-4.568^#^) | 3.125 | TRP 27, ILE 83, HIE 89, ARG 128, LYS 138 |
| 8 | T9*^,#^ | 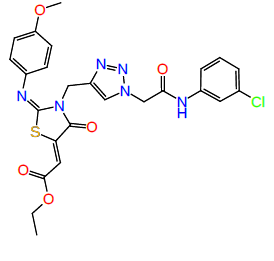 | -7.950*  (-7.969^#^) | 6.25 | TRP 27, ALA 59, ILE 60, THR 87, GLU 136, LYS 138 |
| 9 | Brahm 2^nd^ 15^#^ | 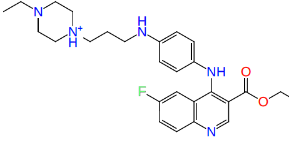 | -8.251 | 12.5 | TRP 27, ALA 59, HIE 89, GLU 136, LYS 138 |
| 10 | NR-341^#^ | 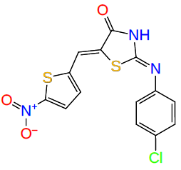 | -7.546 | 12.5 | TRP27, ALA 59, VAL 81, THR 87, LYS 138 |
| 11 | Boga-R-1000-299*^,#^ | 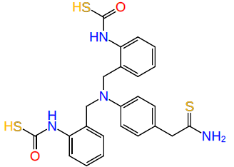 | -0.425*  (-5.022^#^) | 12.5 | TRP 27, HIS 28, GLU 61, THR 87, LYS 138 |
| 12 | NR 302^#^ | 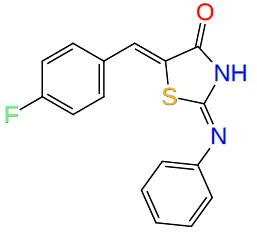 | -8.724 | 25 | TRP 27, ALA 59, VAL 81, |
| 13 | NR 317^#^ | 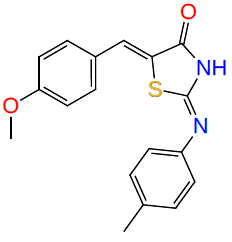 | -8.217 | 25 | ALA 59, VAL 81, ILE 83 |
| 14 | Brahm 2^nd^ 22^#^ | 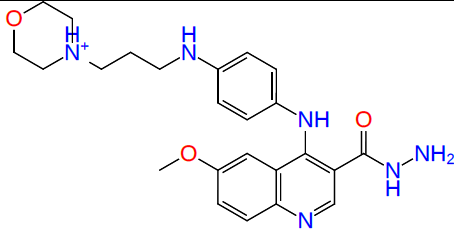 | -8.155 | 25 | TRP 27, ALA 59, ILE 83, HIE 89, ASN 114, GLU 136, LYS 138 |
| 15 | Srihari_28C*^,#^ | 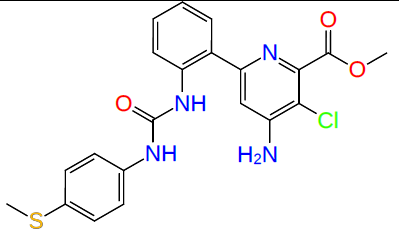 | -8.77*  (-7.614^#^) | 25 | TRP 27, ILE 83, THR 87, ARG 128, GLU 136 |
| 16 | NIPER-TA-5*^,#^ | 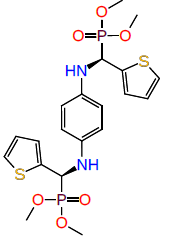 | -9.861*  (-9.651^#^) | 50 | TRP 27, ILE60,THR 87, HIE 89, ARG 128, GLU 136 |
| 17 | Brahm_2^nd^ 23^#^ | 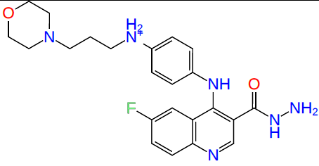 | -9.028 | 50 | TRP 27, ALA 59, HIE 89, ASN 114, GLU 136, LYS 138 |
| 18 | NR 336^#^ | 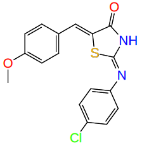 | -8.761 | 50 | ALA 59, VAL 81, ILE 83, THR 87 |
| 19 | GS 407^#^ | 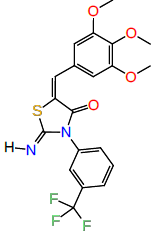 | -8.655 | 50 | ALA 59, THR 87, ARG 128 |
| 20 | Brahm 2^nd^ 16^#^ | 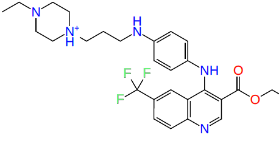 | -8.5 | 50 | TRP 27, ALA 59, HIE 89, GLU 136, LYS 138 |
| 21 | Brahm 2^nd^ 7^#^ | 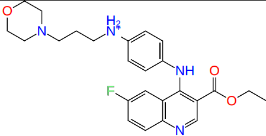 | -8.256 | 50 | TRP 27, ALA 59, HIE 89, GLU 136, LYS 138 |
| 22 | CLRI-ET3A*^,#^ | 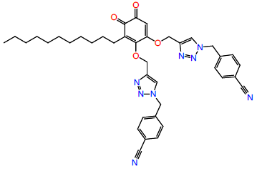 | -8.207*  (-7.72^#^) | 50 | TRP 27, THR 87, GLN 86, ARG 128 |
| 23 | ASN 11105690* | 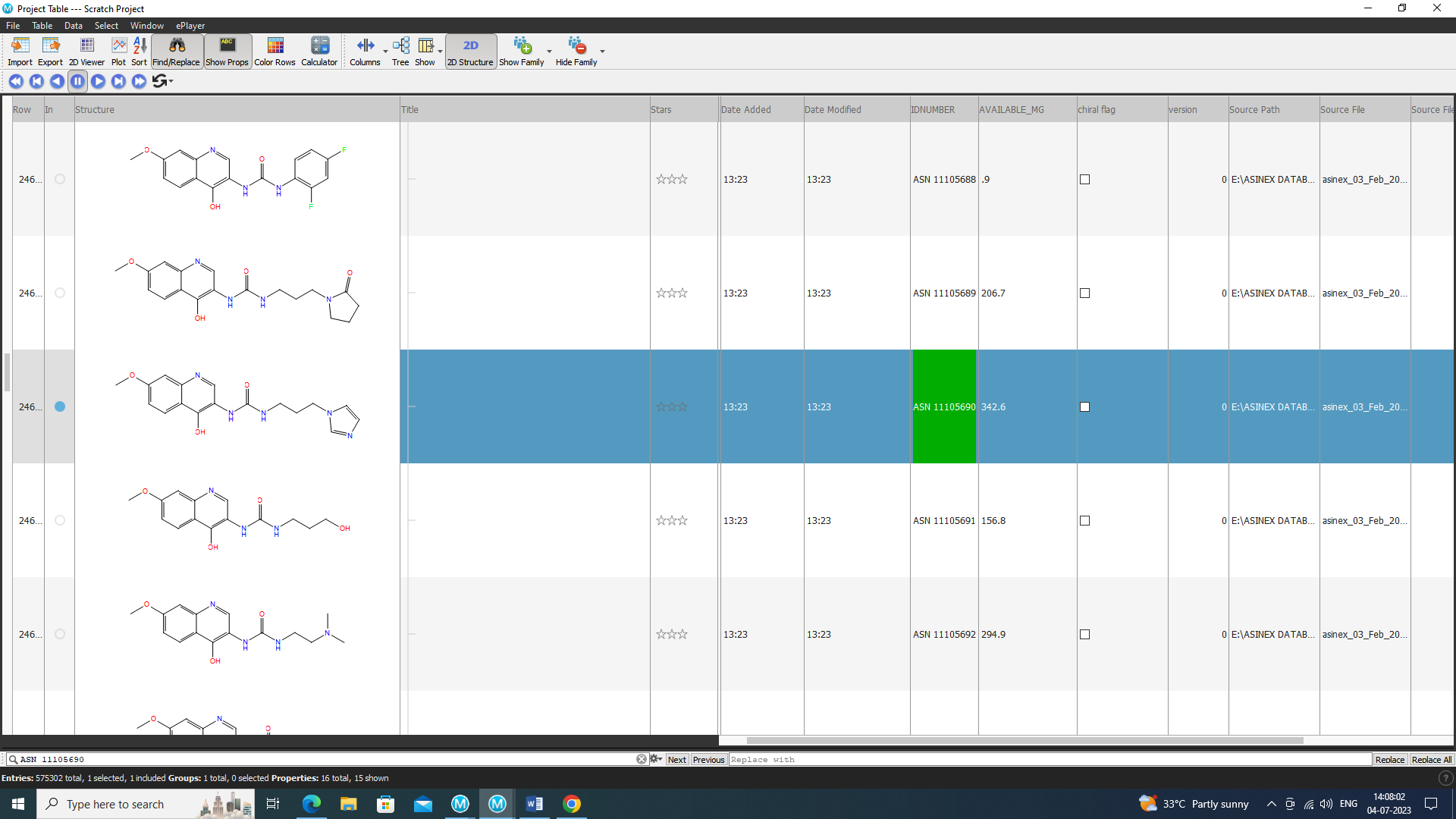 | -11.615 | >50 | TRP 27, ILE 60, VAL 81, ILE 83, GLU 122 |
| 24 | BAS 07023776* | 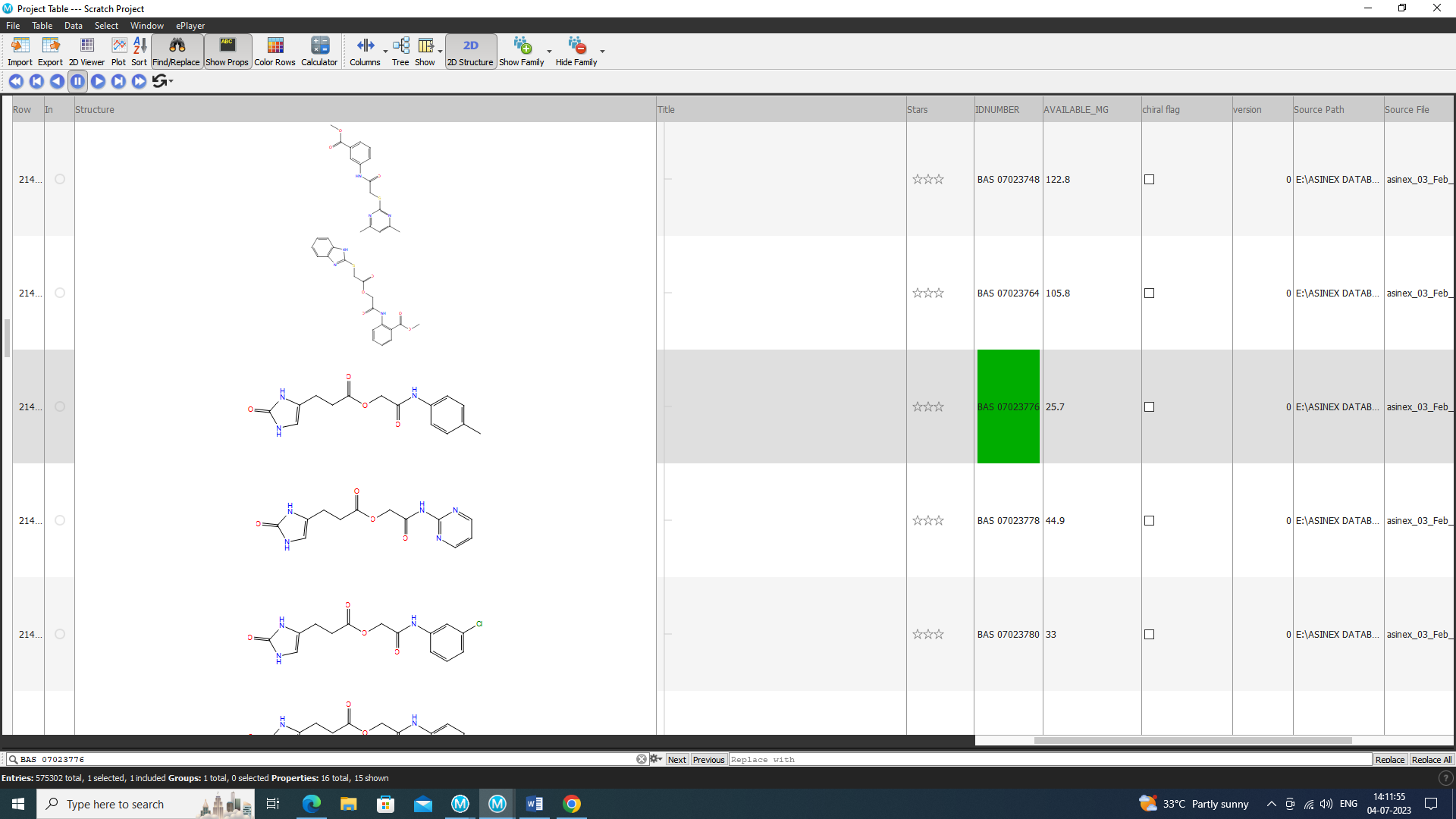 | -11.485 | >50 | ALA 59, ILE 83, GLN 86, THR 87 |
| 25 | BAS 14050851* | 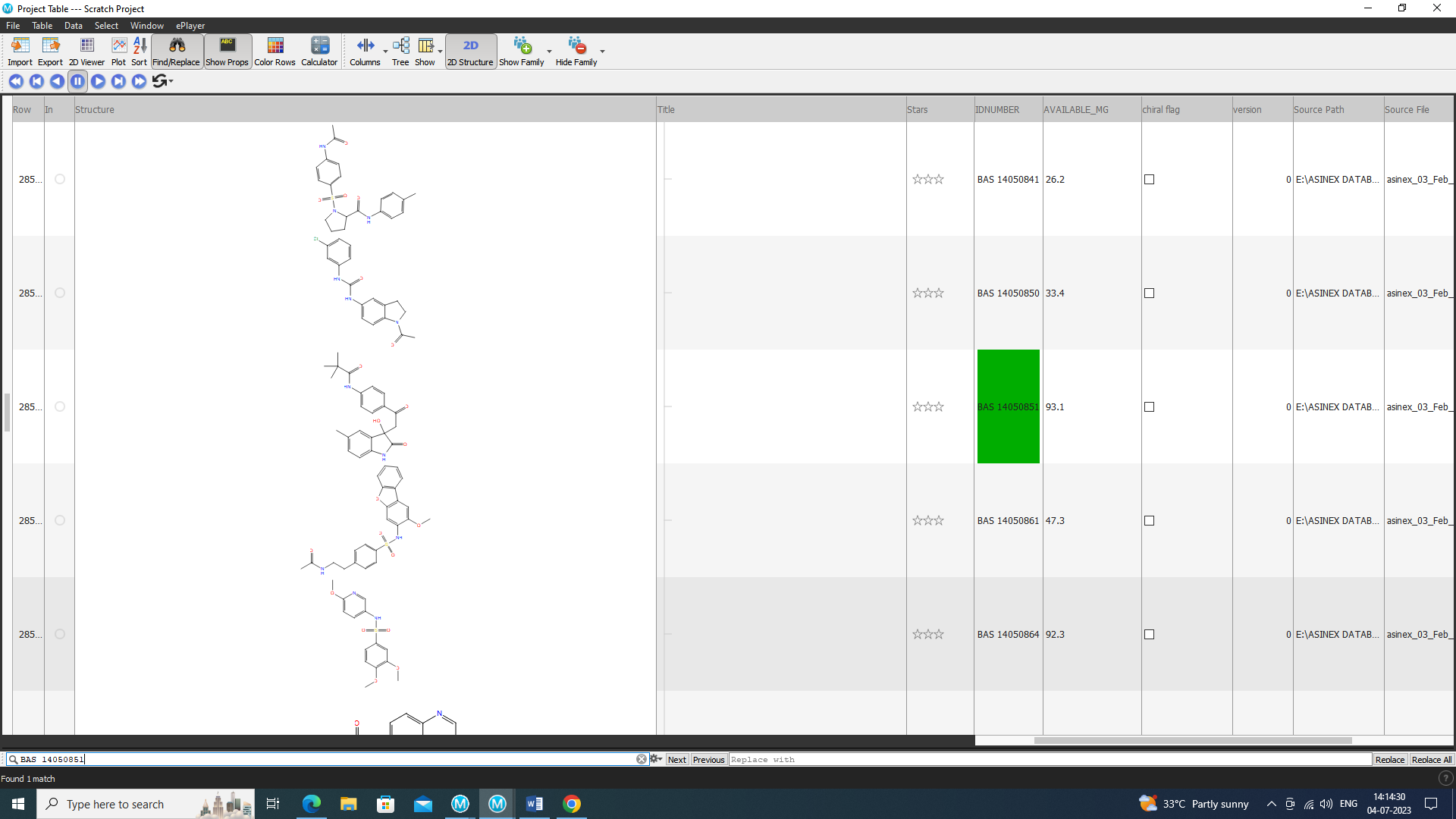 | -11.188 | >50 | TRP 27, HIS 28, ALA 59, ILE 60, ILE 83, LYS 138 |
| 26 | BAS 07805256* |  | -11.086 | >50 | TRP 27, ALA 59, GLU 61, VAL 81, ILE 83, HIE 89, ASN 114 |
| 27 | BAS 00728180* |  | -10.998 | >50 | TRP 27, ALA 59, ILE 83, THR 87, ARG 128, GLU 136 |
| 28 | BAS 00829582* |  | -10.95 | >50 | ALA 59, ILE 60, GLU 61, ASN 114, LYS 138 |
| 29 | BAS 04239203* | 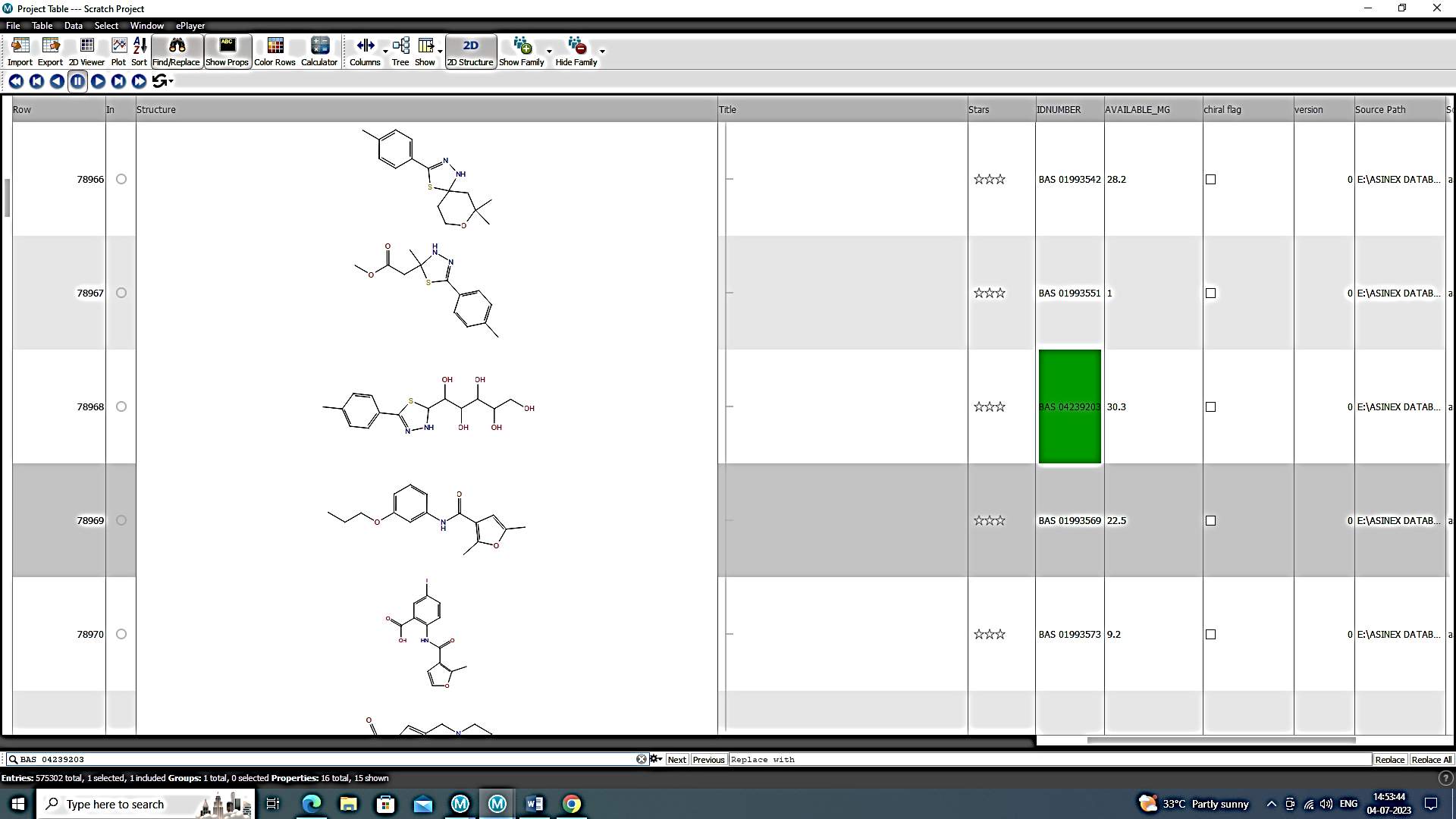 | -10.897 | >50 | TRP 27, ILE 83, GLY 85, THR 87 |
| 30 | BAS 00674022* | 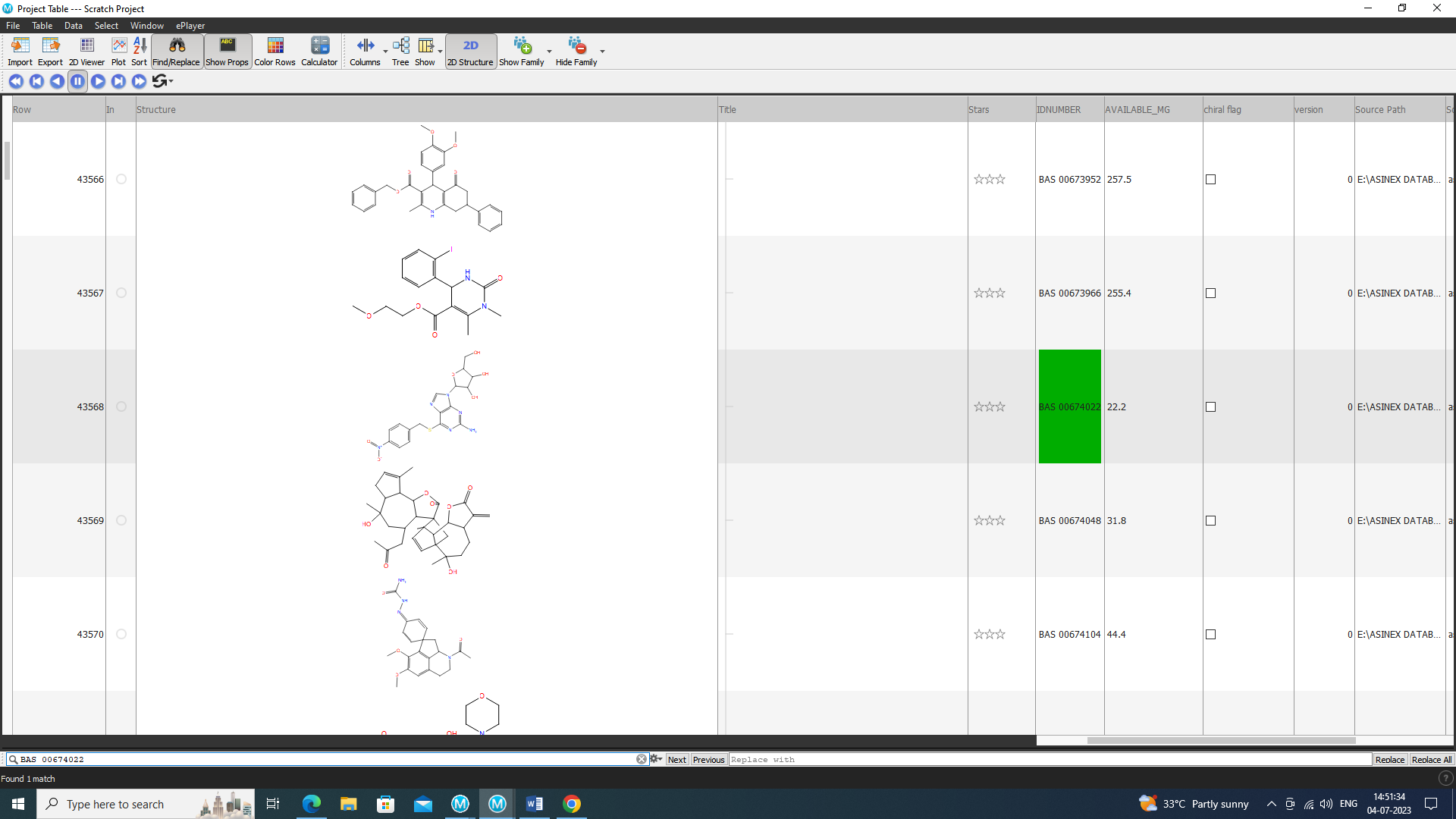 | -10.866 | >50 | SER 25, TRP 27, ALA 59, GLU 61, VAL 81, GLN 86, THR 87, ARG 128, LYS 138 |
| 31 | BAS 03571839* | 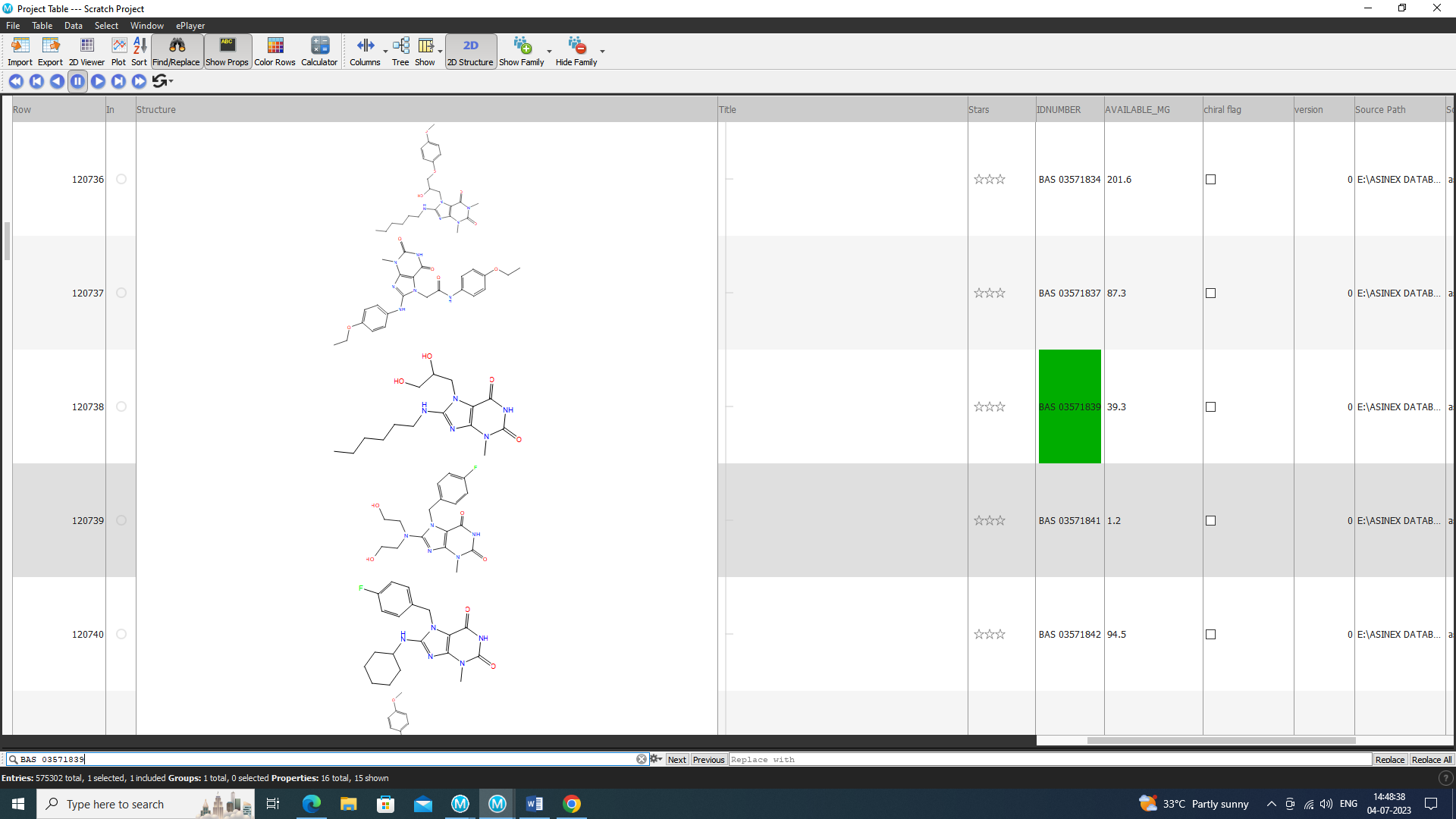 | -10.813 | >50 | TRP 27, HIS 28, ALA 59, VAL 81, ILE 83 |
| 32 | BAS 07805261* | 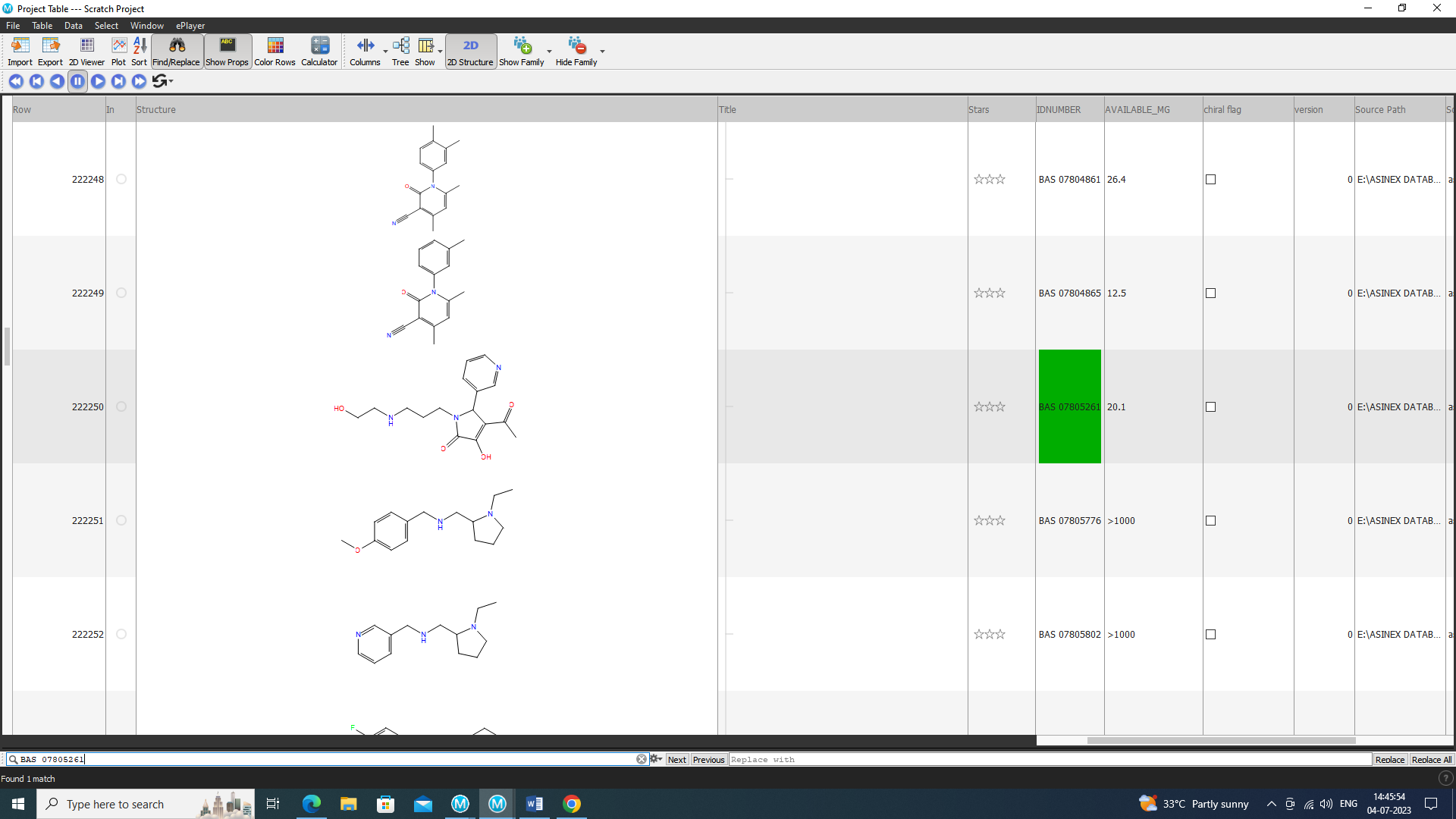 | -10.738 | >50 | GLU 61, VAL 81, ILE 83, HIE 89, ASN 114, LYS 138 |
| 33 | BAS 02327074* | 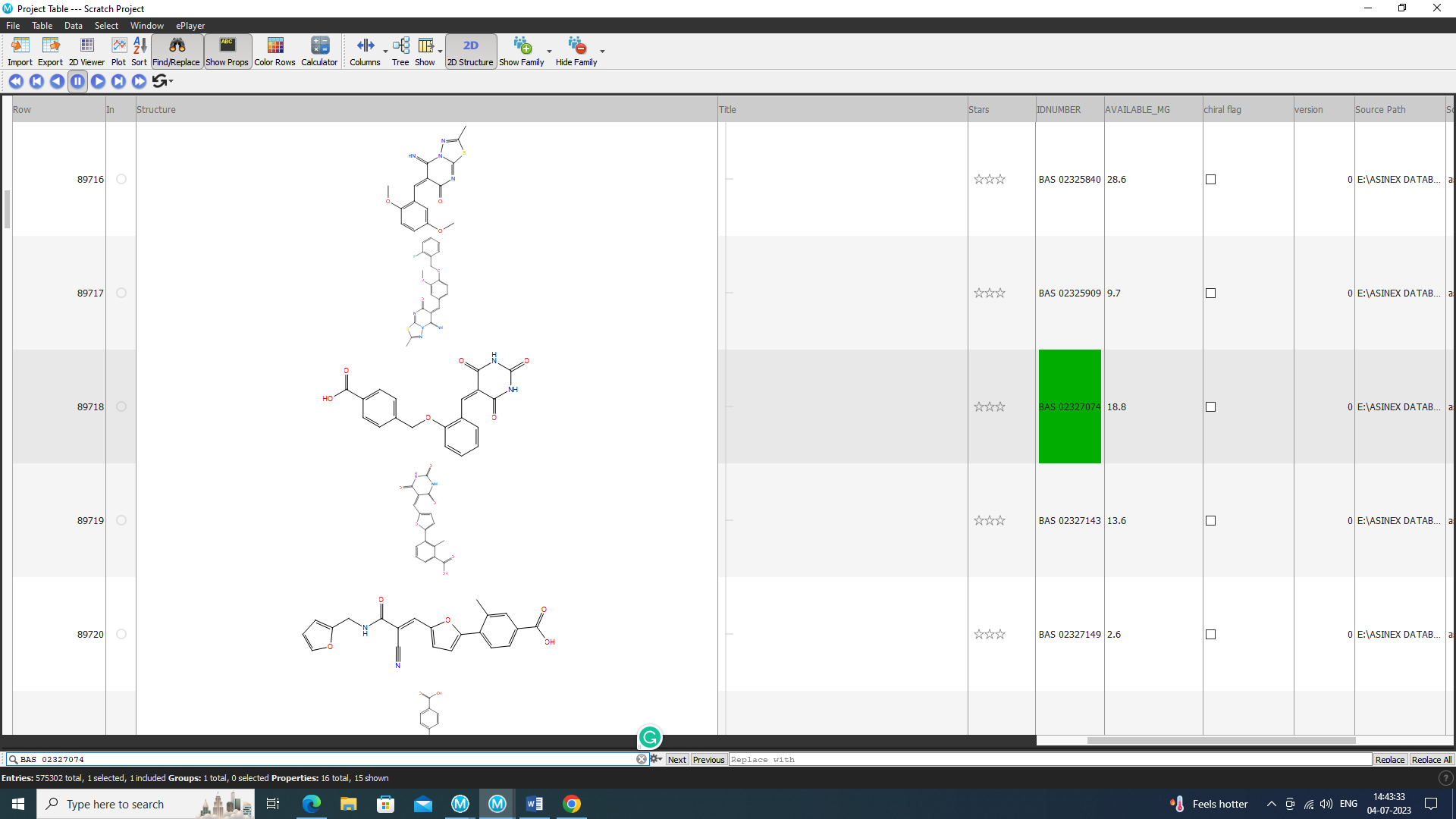 | -10.728 | >50 | TRP 27, THR 87, HIE 89, ASN 114, ARG 128, LYS 138 |
| 34 | ASN 11105540* | 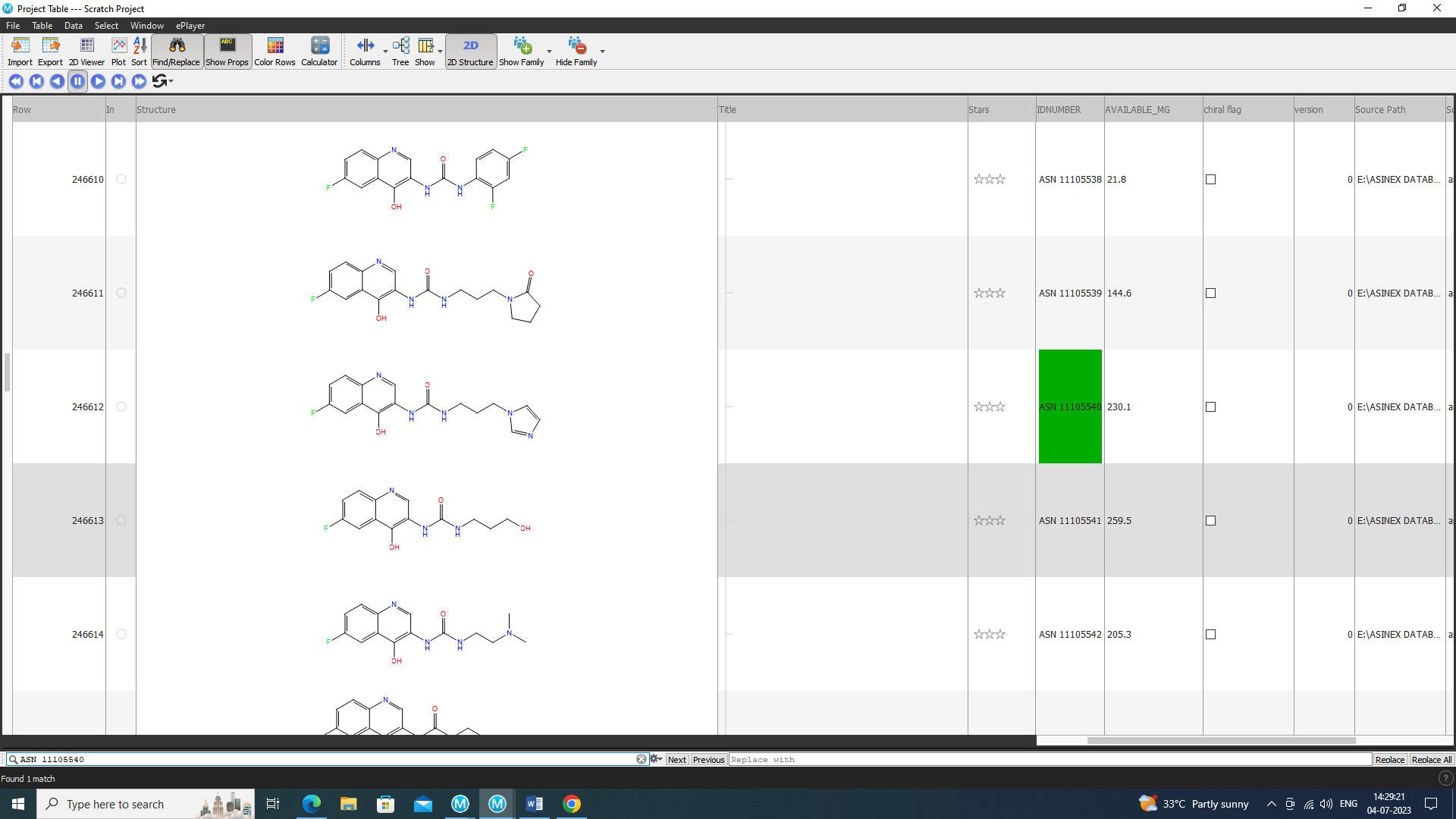 | -10.621 | >50 | TRP 27, VAL 81, ILE 83, GLU 124 |
| 35 | ASN02254483* | 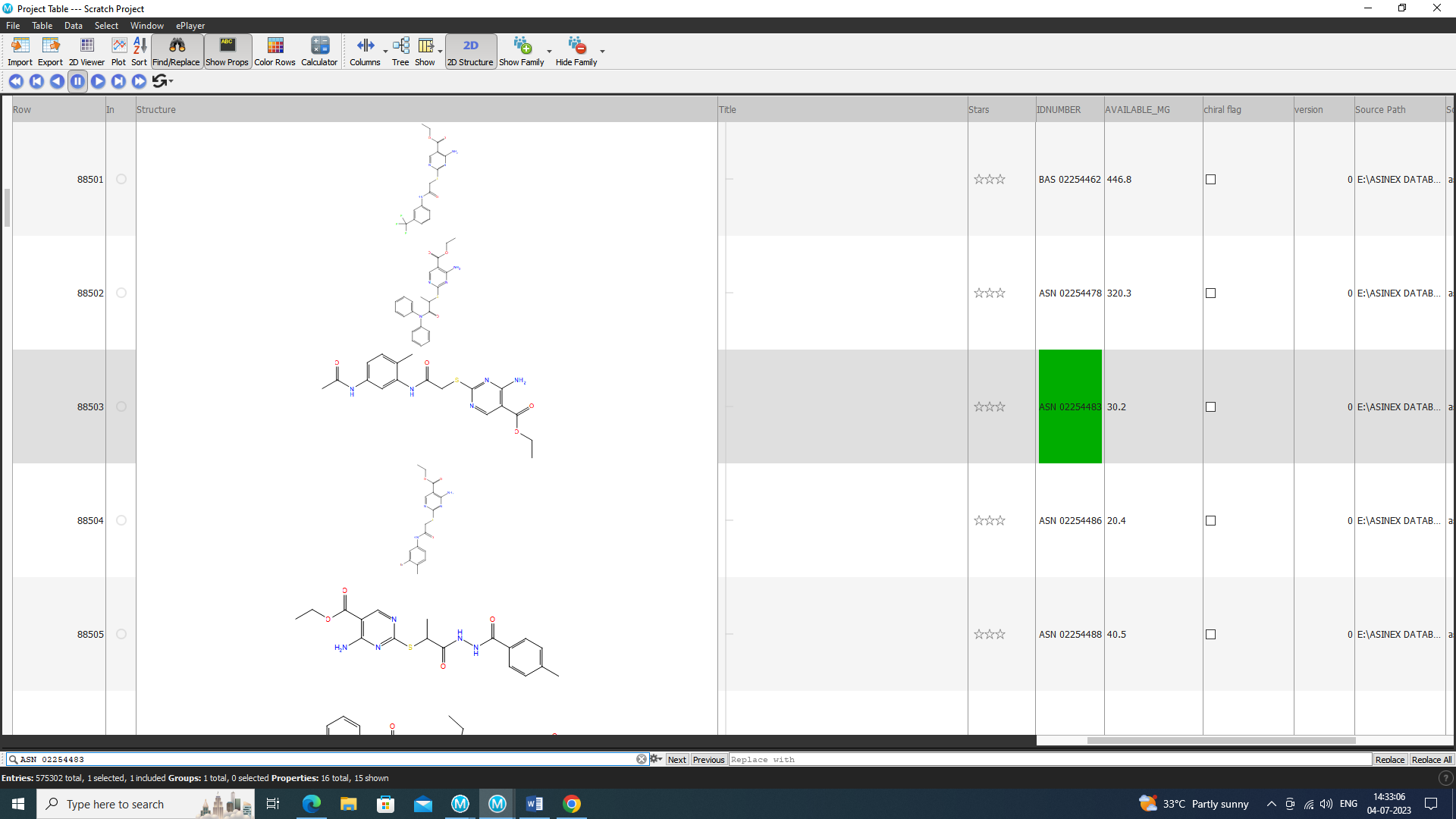 | -9.821 | >50 | TRP 27, ALA 59, VAL 81, ILE 83, ARG 128, GLU 136, LYS 138 |
| 36 | SF Srihari^#^ | 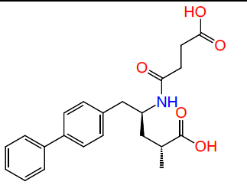 | -9.577 | >50 | TRP 27, ILE 83, THR 87, PHE 90 |
| 37 | NR 346^#^ | 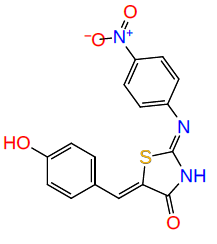 | -9.459 | >50 | ALA 59, VAL 81, THR 87, ASN 114 |
| 38 | ASN14403850* | 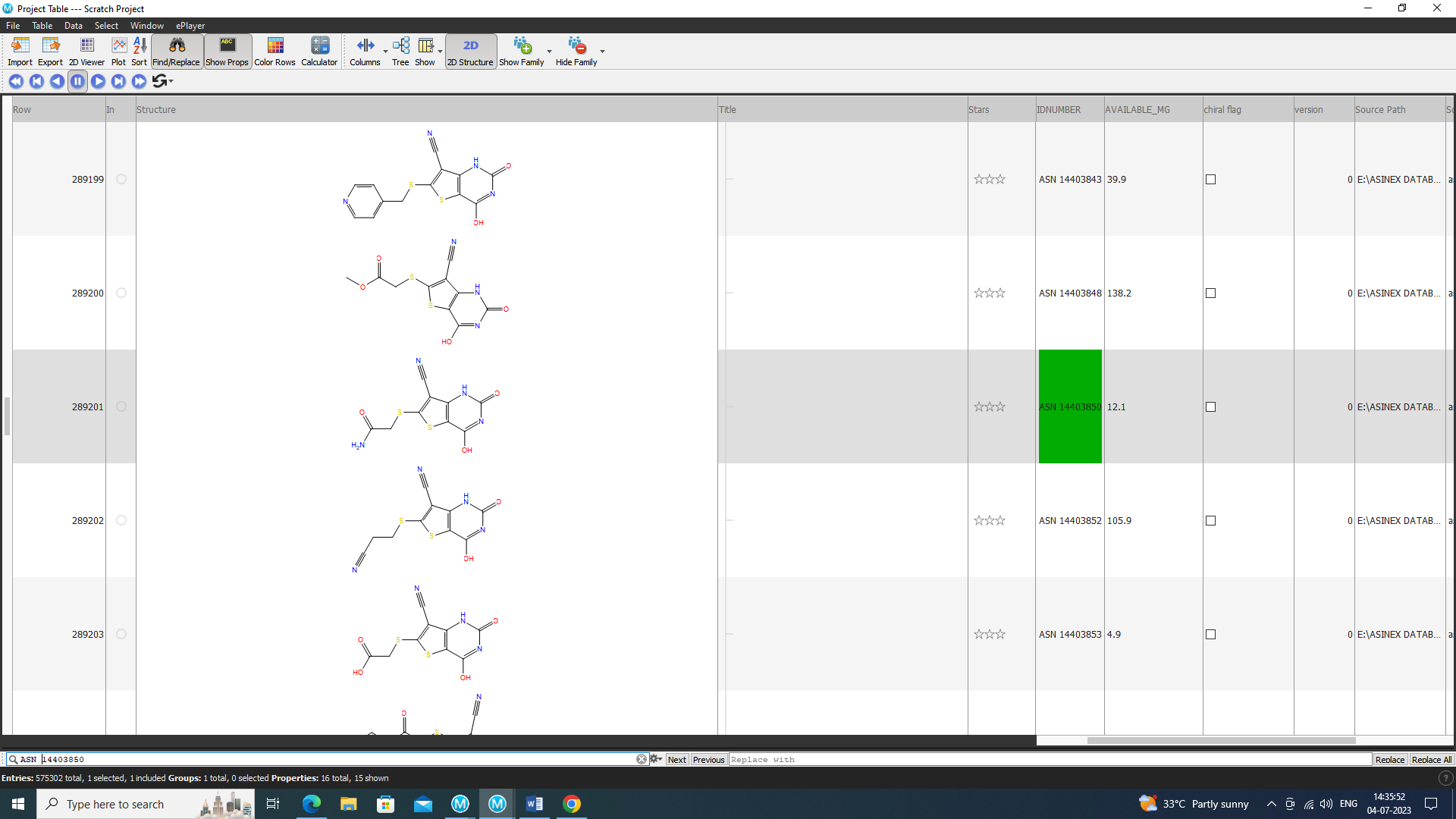 | -9.428 | >50 | VAL 81, ILE 83, GLN 86, THR 87, LYS 138 |
| 39 | SY-Srihari*^,#^ | 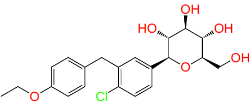 | -9.391*  (-9.203^#^) | >50 | TRP 27, ALA 59, ILE 83, GLU 136, LYS 138 |
| 40 | ASN 03798609* | 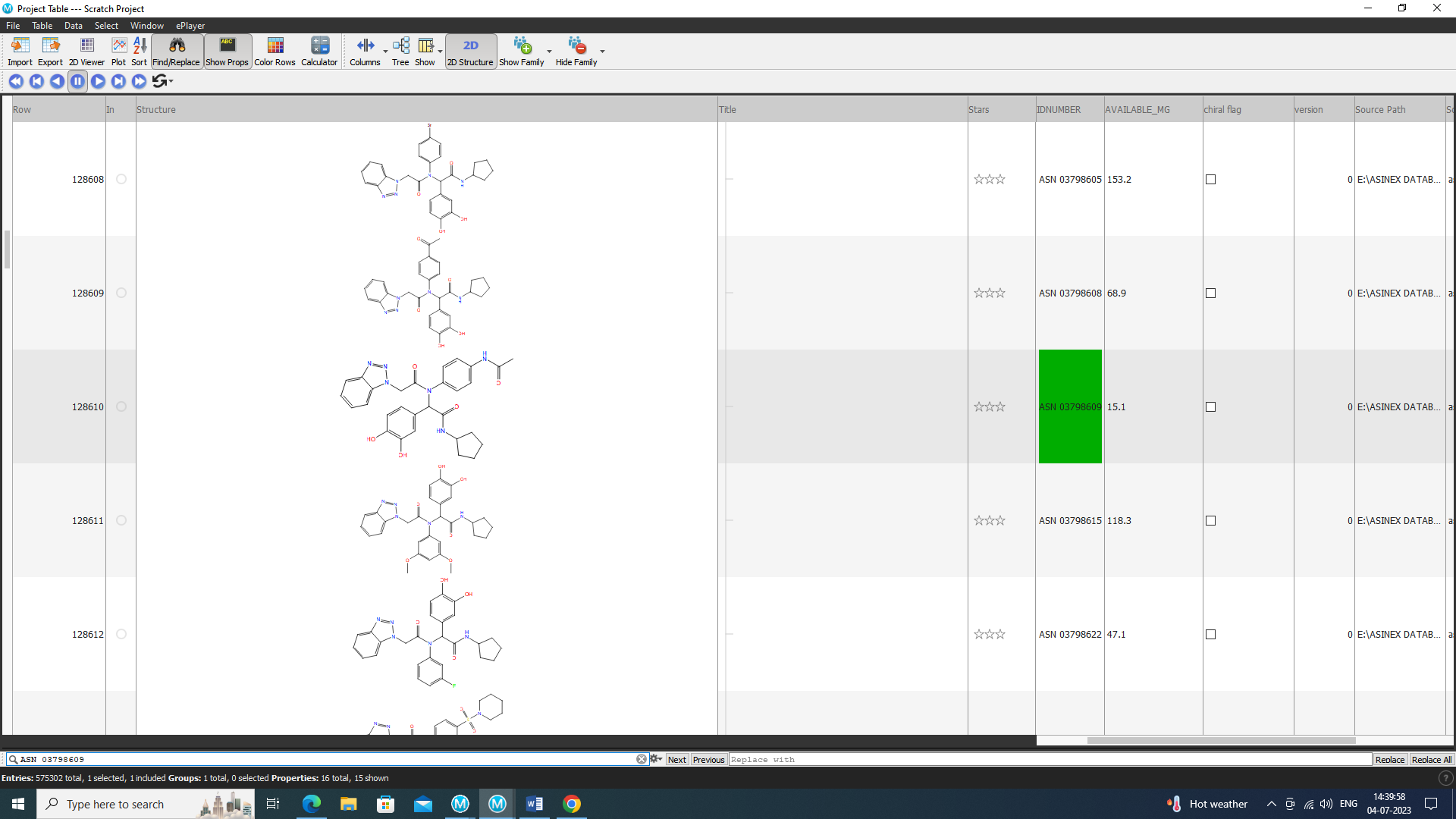 | -8.988 | >50 | TRP 27, HIS 28, ALA 59, VAL 81, HIE 89, ARG 128, GLU 136, LYS 138 |
| 41 | NR 322^#^ | 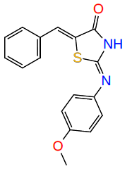 | -8.875 | >50 | TRP 27, ALA 59, VAL 81, ILE 83 |
| 42 | NIPER_TA_VAN* | 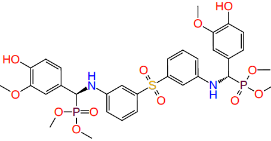 | -7.904*  -8.766^#^ | >50 | TRP 27, ALA 59, ILE 60, VAL 81, ARG 128, GLU 136 |
| 43 | Srihari 26C^#^ | 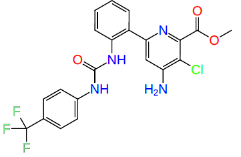 | -8.698 | >50 | TRP 27, ILE 83, THR 87, ARG 128, GLU 136 |
| 44 | NR 325^#^ | 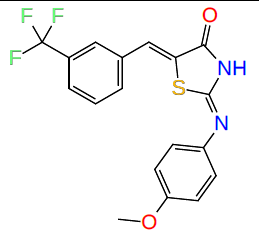 | -8.675 | >50 | ALA 59, VAL 81, ILE 83 |
| 45 | NR 347^#^ | 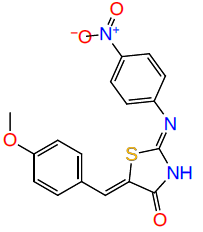 | -8.668 | >50 | ALA 59, VAL 81, ILE 83, GLY 85, ARG 128 |
| 46 | P10^#^ | 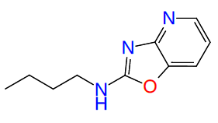 | -8.602 | >50 | ALA 59, ILE 60,GLU 61 |
| 47 | Srihari 24C^#^ | 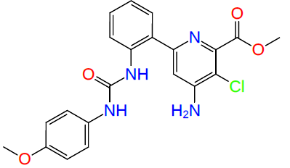 | -8.501 | >50 | TRP 27, ALA 59, ILE 83, GlY 85, THR 87, ARG 128 |
| 48 | NR 324^#^ | 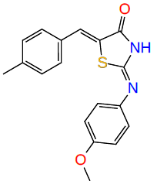 | -8.417 | >50 | ALA 59, VAL 81 |
| 49 | NR 304^#^ | 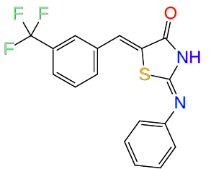 | -8.325 | >50 | ALA 59, VAL 81, ILE 83 |
| 50 | NR 314^#^ | 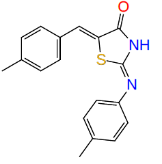 | -8.253 | >50 | TRP 27, ALA 59, VAL 81, ILE 83 |
| 51 | NR 303^#^ | 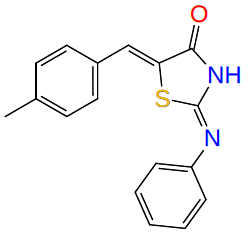 | -8.237 | >50 | ALA 59, VAL 81, ILE 83 |
| 52 | GP-226*^,#^ | 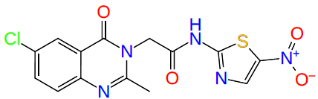 | -7.939*  (-8.179^#^) | >50 | TRP 27, ALA 59, GLY 85, THR 87, HIE 89, PHE 89, ARG 128 |
| 53 | ST Srihari^#^ | 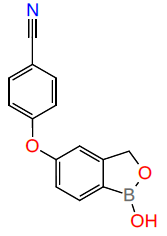 | -8.128 | >50 | TRP 27, ILE 60, GLY 85, THR 87, |
| 54 | Srihari-2M*^,#^ | 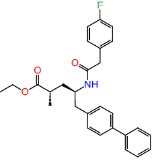 | -7.756*  (-7.37^#^) | >50 | TRP 27, THR 87, HIE 89, ARG 128, LYS 138 |
| 55 | T8*^,#^ | 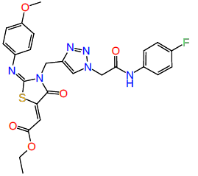 | -8.821*  (-6.143^#^) | >50 | TRP 27, ALA 59, ILE 83, ARG 128 |
| 56 | B2-39-3*^,#^ | 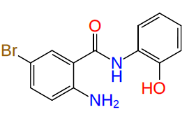 | -0.200*  (-4.899^#^) | >50 | TRP 27, ALA 59, VAL 81, HIE 89, ASN 114 |
| 57 | RN-LR-17*^,#^ | 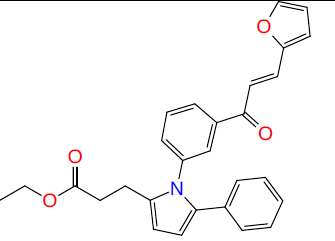 | -7.986*  (-3.851^#^) | >50 | HIE 89, ASN 114, ARG 128, LYS 138 |
| * Compounds screened by pharmacophore based virtual screening (PBVS)  ^#^ Compounds screened by Structure-based flexible docking  ^*,#^ Compounds screened by both pharmacophore based virtual screening (PBVS) and Structure-based flexible docking  -BITS *in-house* compound library was screened by 2 molecular docking methods and docking score outside bracket is obtained by e-pharmacophore based virtual screening and docking score inside the brackets denote those obtained by flexible docking.  -Compounds with code name ASN or BAS belong to ASINEX library (15 compounds in total) and rest of the compounds belong to BITS-*in-house* library.  - MIC table reflects solely the data w.r.t. primary screening of the compounds in a single run. Based on this primary round, compounds with MIC ranging between 6.25-0.78 µg/ml were shortlisted for secondary and tertiary screening (data not shown in the paper). Further shortlisting was done from these rounds, to select the top 3 compounds with MIC ranging between 1.56-0.78 µg/ml. Since only 3 shortlisted hits consistently showed potent antimycobacterial activity, they were then tested more rigorously using a variety of methods discussed in the main body of the manuscript. | | | | | |

**Supplementary Table 2: Input Protein Sequences used for various bioinformatic analysis**

| **Sl. No.** | **Mycobacterial species** | **Lumazine synthase (RibH) protein sequence** |
| --- | --- | --- |
| 1 | *Mycobacterium tuberculosis (strain ATCC 25618 / H37Rv)* | >sp\|P9WHE9\|RISB_MYCTU OS=Mycobacterium tuberculosis (strain ATCC 25618 / H37Rv) OX=83332 GN=ribH PE=1 SV=1  MKGGAGVPDL PSLDASGVRL AIVASSWHGK ICDALLDGAR KVAAGCGLDD PTVVRVLGAI  EIPVVAQELA RNHDAVVALG VVIRGQTPHF DYVCDAVTQG LTRVSLDSST PIANGVLTTN  TEEQALDRAG LPTSAEDKGA QATVAALATA LTLRELRAHS |
| 2 | *Mycobacterium* abscessus | >sp\|B1MCA3\|RISB_MYCA9 OS=Mycobacteroides abscessus (strain ATCC 19977 / DSM 44196 / CCUG 20993 / CIP 104536 / JCM 13569 / NCTC 13031 / TMC 1543 / L948) OX=561007 GN=ribH PE=3 SV=1  MSGDGIPSLS VGDASGISLA IVASTWHDQI CTALLEGAQR VATEAGIDRP TVVRVLGAIE  IPVVAQALAR THDAVVALGV VIQGETPHFG YVCDAVTTGL TRVSLDTSTP VANGVLTVNN  EQQAIDRAGL PGSAEDKGAQ AAAAALDTAL TLRRLRQPWA |
| 3 | *Mycobacterium tuberculosis variant africanum* | >tr\|A0A120J0T0\|A0A120J0T0_MYCTX OS=Mycobacterium tuberculosis variant africanum OX=33894 GN=ribH PE=3 SV=1  MKGGAGVPDL PSLDASGVRL AIVASSWHGK ICDALLDGAR KVAAGCGLDD PTVVRVLGAI  EIPVVAQELA RNHDAVVALG VVIRGQTPHF DYVCDAVTQG LTRVSLDSST PIANGVLTTN  TEEQALDRAG LPTSAEDKGA QATVAALATA LTLRELRAHS |
| 4 | *Mycobacterium bovis* | >sp\|P66035\|RISB_MYCBO OS=Mycobacterium bovis (strain ATCC BAA-935 / AF2122/97) OX=233413 GN=ribH PE=3 SV=1  MKGGAGVPDL PSLDASGVRL AIVASSWHGK ICDALLDGAR KVAAGCGLDD PTVVRVLGAI  EIPVVAQELA RNHDAVVALG VVIRGQTPHF DYVCDAVTQG LTRVSLDSST PIANGVLTTN  TEEQALDRAG LPTSAEDKGA QATVAALATA LTLRELRAHS |
| 5 | *Mycobacterium tuberculosis variant bovis BCG* | >tr\|A0A0K2HV99\|A0A0K2HV99_MYCBI OS=Mycobacterium tuberculosis variant bovis BCG OX=33892 GN=ribH PE=3 SV=1  MKGGAGVPDL PSLDASGVRL AIVASSWHGK ICDALLDGAR KVAAGCGLDD PTVVRVLGAI  EIPVVAQELA RNHDAVVALG VVIRGQTPHF DYVCDAVTQG LTRVSLDSST PIANGVLTTN  TEEQALDRAG LPTSAEDKGA QATVAALATA LTLRELRAHS |
| 6 | *Mycobacterium canettii* | >tr\|A0A8I0EMM5\|A0A8I0EMM5_9MYCO OS=Mycobacterium canettii OX=78331 GN=ribH PE=3 SV=1  MTGGAGVPDL PSLDASGVRL AIVASSWHGK ICDALLDGAR NVAAGCGLDD PTVVRVLGAI  EIPVVAQELA RNHDAVVALG VVIRGQTPHF DYVCDAVTQG LTRVSLDSST PIGNGVLTTN  TEEQALDRAG LPTSAEDKGA QATVAALATA LTLRELRAQP |
| 7 | *Mycobacterium orygis* | >tr\|A0A829C689\|A0A829C689_9MYCO OS=Mycobacterium orygis 112400015 OX=1305739 GN=ribH PE=3 SV=1  MPDLPSLDAS GVRLAIVASS WHGKICDALL DGARKVAAGC GLDDPTVVRV LGAIEIPVVA  QELARNHDAV VALGVVIRGQ TPHFDYVCDA VTQGLTRVSL DSSTPIANGV LTTNTEEQAL  DRAGLPTSAE DKGAQATVAA LATALTLREL RAHS |
| 8 | *Mycobacterium tuberculosis variant pinnipedii* | >tr\|A0A328GNP2\|A0A328GNP2_MYCTX OS=Mycobacterium tuberculosis variant pinnipedii OX=194542 GN=ribH PE=3 SV=1  MKGGAGVPDL PSLDASGVRL AIVASSWHGK ICDALLDGAR KVAAGCGLDD PTVVRVLGAI  EIPVVAQELA RNHDAVVALG VVIRGQTPHF DYVCDAVTQG LTRVSLDSST PIANGVLTTN  TEEQALDRAG LPTSAEDKGA QATVAALATA LTLRELRAHS |
| 9 | *Mycobacterium avium* | >sp\|A0QI08\|RISB_MYCA1 OS=Mycobacterium avium (strain 104) OX=243243 GN=ribH PE=3 SV=1  MSPAAGVPEM PALDASGVRL GIVASTWHSR ICDALLAGAR KVAADSGVEN PTVVRVLGAI  EIPVVAQELA RNHDAVVALG VVIRGQTPHF EYVCDAVTQG ITRVSLDAST PVANGVLTTD  NEQQALDRAG LPDSAEDKGA QAAGAALSAA LTLRELRARS |
| 10 | *Mycobacterium kansasii* | >tr\|X7Y0E1\|X7Y0E1_MYCKA OS=Mycobacterium kansasii 824 OX=1299328 GN=ribH PE=3 SV=1  MSGGAGVPDI PALDASGVRL AIVASTWHSQ ICDALLAGAR KVAADSGIDN PTVVRVLGAI  EIPVVAQELA RQHDAVVALG VVIRGETPHF DYVCDAVTQG LTRVSLDEST PVANGVLTTN  TEEQALDRAG LPTSAEDKGA QATAAALTTA LTLRELRVQS |
| 11 | *Mycobacteroides chelonae* | >tr\|A0A1S1LMU2\|A0A1S1LMU2_MYCCH OS=Mycobacteroides chelonae OX=1774 GN=ribH PE=3 SV=1  MSGEGIPSLT VGDASSLSLA IVASTWHDQI CTALLEGAKR VAADAGIDRP TVVRVLGAIE  IPVVAQALAR THDAVVALGV VIQGETPHFN YVCDAVTTGL TRVSLDTSTP VANGVLTVNN  EQQALDRAGL PESSEDKGAQ AAAAALDTAL TLRRLRQPWT EA |
| 12 | *Mycolicibacterium fortuitum* | >tr\|A0A1A0TGF6\|A0A1A0TGF6_MYCFO OS=Mycolicibacterium fortuitum OX=1766 GN=ribH PE=3 SV=1  MSAHGVPDLP QVDASNVKLA IVASTWHTQI CDALLDGARK VAADAGIAEP TVVRVLGAIE  IPVVAQALAA THDAVVALGV VIRGQTPHFD YVCDAVTQGL TRVSLDASTP VANGVLTTDN  EAQALDRAGL PDSTEDKGAQ AAAAALSTAL TLRELRSKA |
| 13 | *Mycobacterium intracellulare* | >tr\|X8AJW1\|X8AJW1_MYCIT OS=Mycobacterium intracellulare OX=1767 GN=ribH PE=3 SV=1  MSPAEGVPEV PPLDASGLRL ALVASTWHSE ICDALLAGAS KVASESGVDD PTVVRVIGAI  EIPVVAQELA RNHDAVVALG VVIRGQTPHF EYVCDAVTQG LTRVSLDTST PVANGVLTTD  TEQQALDRAG LPESAEDKGA QATLAALTTA LTLRELRARS |
| 14 | *Mycobacterium malmoense* | >tr\|A0A1S2WFZ2\|A0A1S2WFZ2_MYCMA OS=Mycobacterium malmoense OX=1780 GN=ribH PE=3 SV=1  MSGGAGVPEI PALDASGLRL GIVASTWHGQ ICDALLAGAR KMAAESGVEN PTVVRVLGAI  EIPVVAQQLA RDHDAVVALG VVIRGETPHF DYVCDSVTQG LTRVSLDACT PIGNGVLTTN  TEEQALARAG LPASAEDKGA QAAGAALTAA LTLRDLRARS |
| 15 | *Mycobacterium simiae* | >tr\|A0A5B1BKW0\|A0A5B1BKW0_MYCSI OS=Mycobacterium simiae OX=1784 GN=ribH PE=3 SV=1  MSGGAGVPDI PALDASGVRL AIVASTWHAE ICDALLAGAR KVATESGIDN PTVARVLGAI  EIPVLAQELA RSHDAVVALG VVIRGETPHF DYVCDAVTQG LTRVSLDAST PVANGVLTTN  TEEQARDRAG LPTSAEDKGA QATAAALTTA LALRELRAQS |
| 16 | *Mycobacterium xenopi* | >tr\|A0A2X1TE33\|A0A2X1TE33_MYCXE OS=Mycobacterium xenopi OX=1789 GN=ribH PE=3 SV=1  MSPAAGMPEL PALDASGLKL AIVASTWHTE ICDALLTGAR KTAAEWGIDD PTVVRVLGAI  EIPVVAQELA RSHDAVVALG VVIRGETPHF DYVCDAVTQG LTRVSLDAST PVANGVLTTN  TEEQARDRAG LPTSTEDKGA QATAAALNTA LTLRELRTRS |
| 17 | *Mycobacterium gordonae* | >tr\|A0A0Q2RQN3\|A0A0Q2RQN3_MYCGO OS=Mycobacterium gordonae OX=1778 GN=ribH PE=3 SV=1  MSGGAGVPEM PVLDASGLRL AIVASTWHDK ICDALLAGAR KVATDSGIDT PTVVRVLGAI  EIPVVAQELA RNHDAVVALG VVIRGETPHF DYVCDAVTQG LTRVSLDAST PVANGVLTTN  TEAQALDRAG LPSSAEDKGA QAAAAALTTA LTLRDLRAQS |
| 18 | *Mycobacterium celatum* | >tr\|A0A1X1RSX5\|A0A1X1RSX5_MYCCE OS=Mycobacterium celatum OX=28045 GN=ribH PE=3 SV=1  MSPAAGVPDM PALDASSVKL AIVASTWHTT ICDALLGGAR KTAAEWGVDD PTVVRVLGAI  EIPVVAQALA REHDAVVALG VVIRGQTPHF DYVCDAVTQG LTRVSLDAST PVANGVLTTN  TEEQALDRAG LPDSAEDKGA QATAAALSTA LTLRDLRAKP |
| 19 | *Mycobacterium haemophilum* | >tr\|A0A0I9TYM3\|A0A0I9TYM3_9MYCO OS=Mycobacterium haemophilum OX=29311 GN=ribH PE=3 SV=1  MSGGAGVPEV PAIDASGLRL GIVASTWHGR ICDALLAGAR KVAANSGVDN PTVVRVLGAI  EIPVVAQELA RTHDAVVALG VVIRGATPHF DHVCNSVTQG LTRVALDTST PVGNGVLTTN  TEEQALDRAG LPTSAEDKGA QAAAAALTTA LTLLNLRART |
| 20 | *Mycobacterium ulcerans* | >sp\|A0PPL7\|RISB_MYCUA OS=Mycobacterium ulcerans (strain Agy99) OX=362242 GN=ribH PE=3 SV=1  MSGGAGIPDV PAFDASGVRL AIVASTWHTK ICDALLAGAR NTAADSGIDN PTVVRVLGAI  EIPVVAQELT RNHDAVVALG VVIRGETPHF DYVCDVVTQG LTRVSLDSST PVANGVLTTN  SEEQALNRAG LPTSDEDKGA QATAAALTTA LTLRELRAES |
| 21 | *Mycobacterium marinum* | >sp\|B2HP68\|RISB_MYCMM OS=Mycobacterium marinum (strain ATCC BAA-535 / M) OX=216594 GN=ribH PE=3 SV=1  MSGGAGIPDV PAFDASDVRL AIVASTWHTK ICDALLAGAR NTAADSGIDN PTVVRVLGAI  EIPVVAQELT RNHDAVVALG VVIRGETPHF DYVCDAVTQG LTRVSLDSST PVANGVLTTN  SEEQALNRAG LPTSDEDKGA QATAAALTTA LTLRELRAEA |
| 22 | *Mycobacterium nonchromogenicum* | >tr\|A0A1X1ZI26\|A0A1X1ZI26_MYCNO OS=Mycobacterium nonchromogenicum OX=1782 GN=ribH PE=3 SV=1  MSPAAGVPDM PALDASDVRL AIVASTWHTR ICDALLAGAR RVAAEAGIPE PTVVRVLGAI  EIPVVTQEVA RNHDAVVALG VVIRGATPHF DYVCDAVTQG LTRVSLDAAT PVANGVLTVN  DEEQALDRAG LPGSTEDKGA QAAAAALSTA LTLRELRAAP |
| 23 | *Mycobacterium parascrofulaceum* | >tr\|D5PHU0\|D5PHU0_9MYCO OS=Mycobacterium parascrofulaceum ATCC BAA-614 OX=525368 GN=ribH PE=3 SV=1  MEPCDRRRQD DAVRVSPAWG TAQVSGSGEP EIPALDATGL RLGIVASTWH GKICEALLQG  ARRVAAESGI DNPTVVRVLG AIEIPVVAQE LTRNHDAVIA LGVVIRGETP HFSYVCDAVT  QGLTRVALDA STPVANGVLT TNTEQQALDR AGLPESVEDK GAQAAAAALT AALTLRDLRA  RP |
| 24 | *Mycobacterium szulgai* | >tr\|A0A1X2E6B9\|A0A1X2E6B9_MYCSZ OS=Mycobacterium szulgai OX=1787 GN=ribH PE=3 SV=1  MSGGAGVPEM PVLDAADVQL AIVASTWHTE VCDALLAGAL KVAADSGIPS PTVVRVLGAI  EIPVVVQELA RSHDAVVALG VVIRGETPHF DYVCDAVTQG LTRVSLDEST PVGNGVLTTN  TEEQALDRAG LPTSAEDKGA QATAAALATA LTLRDLRAQS |
| 25 | *Mycobacterium scrofulaceum* | >tr\|A0A1A2TYK3\|A0A1A2TYK3_MYCSC OS=Mycobacterium scrofulaceum OX=1783 GN=ribH PE=3 SV=1  MSGSGEPDIP ALDASGLRVG IVASTWHSKI CEALLDGARR VAAESGIDNP TVVRVLGAIE  LPVVAQELAR NHDAVIALGV VIRGGTPHFE YVCDAVTQGL TRVALDASTP VANGVLTTNT  EQQALDRAGL PESVEDKGAQ AAGAALTAAL TLRELRARS |
| 26 | *Mycobacterium tuberculosis variant microti* | >PLV44826.1 6,7-dimethyl-8-ribityllumazine synthase [Mycobacterium tuberculosis variant microti OV254]  MSGAGIPDLGELDASGLRLGIVASTWHSTICDALLAGAERVVARSGVDNPTVVRVHGAIEIPVVAQELAR  NHDAVIALGVVIRGGTPHFEYVCDAVTQGLTRVSLDASTPVANGVLTTNTEEQALDRAGLPTSAEDKGAQAAGAALTAALALRDLRARS |
| 27 | *Mycobacterium terrae* | >BBX22440.1 6,7-dimethyl-8-ribityllumazine synthase [Mycolicibacter terrae]  MSGTGVPDMPALDASGVRLAIVASTWHTRICDALLAGARKVAADAGVADPTVVRVLGAIEIPVVAQEVAR  NHDAVVALGVVIRGGTPHFDYVCDAVTQGLTRVSLDAATPVANGVLTVNDEQQALDRAGLPGSAEDKGAQAAAAALSTALTLRDLRAAL |
| 28 | *Mycobacterium tuberculosis variant caprae* | >PRH94793.1 6,7-dimethyl-8-ribityllumazine synthase [Mycobacterium tuberculosis variant caprae]  MKGGAGVPDLPSLDASGVRLAIVASSWHGKICDALLDGARKVAAGCGLDDPTVVRVLGAIEIPVVAQELA  RNHDAVVALGVVIRGQTPHFDYVCDAVTQGLTRVSLDSSTPIANGVLTTNTEEQALDRAGLPTSAEDKGA  QATVAALATALTLRELRAHS |
| 29 | *M. leprae* | >[M. leprae]  MSGGAGIPEVPGIDASGLRLGIVASTWHSRICDALLAGARKVAADSGIDGPTVVRVLGAI EIPVVVQELARHHDAVVALGVVIRGDTPHFDYVCNSVTQGLTRIALDTSTPVGNGVLTTN TEKQALDRAGLPTSAEDKGAQAAAAALTTALTLLNLRSRI |

**Sequence alignment of M. tb H37Rv RibH protein with that of *M. leprae***

| Score | Expect | Method | Identities | Positives | Gaps |
| --- | --- | --- | --- | --- | --- |
| 239 bits(610) | 5e-84 | Compositional matrix adjust. | 125/158(79%) | 142/158(89%) | 0/158(0%) |

Query 1 MKGGAGVPDLPSLDASGVRLAIVA**S**S**WH**GKICDALLDGARKVAAGCGLDDPTVVRV**LGAI** 60

M GGAG+P++P +DASG+RL IVA**S**+**WH** +ICDALL GARKVAA G+D PTVVRV**LGAI**

Sbjct 1 MSGGAGIPEVPGIDASGLRLGIVASTWHSRICDALLAGARKVAADSGIDGPTVVRVLGAI 60

Query 61 **E**IPVVAQELARNHDAVVALG**VVI**R**G**Q**T**P**HF**DY**V**CDAVTQGLTRVSLDSS**TPIANG**VLTTN 120

**E**IPVV QELAR+HDAVVALG**VVI**R**G**-**T**P**HF**DY**V**C++VTQGLTR++LD+STP**+**-**N**GVLTTN

Sbjct 61 EIPVVVQELARHHDAVVALGVVIRGDTPHFDYVCNSVTQGLTRIALDTSTPVGNGVLTTN 120

Query 121 TEEQALD**R**AGLPTSA**E**D**K**GA**QA**TV**A**ALATALTLRELRA 158

TE+QALD**R**AGLPTSA**E**D**K**GA**QA—-A**AL-TALTL--LR+

Sbjct 121 TEKQALDRAGLPTSAEDKGAQAAAAALTTALTLLNLRS 158

**Supplementary Table 3. List of bacterial strains used in this study**

| **Bacterial Strain** | **Description** | **Reference** |
| --- | --- | --- |
| *M. tb* strain  *H37Rv* | Obtained from ATCC (ATCC27294 strain), TMC 102 [H37Rv] | https://www.atcc.org/products/27294 |
| *M. tb H37Rv*  mc^2^ 7902 | Auxotroph strain (H37Rv Δ*pan*CD ΔleuCD ΔargB) | Vilchèze C, *et al*. (Ref. 41) |
| *M. tb ribH-KD* | Auxotroph strain (H37Rv Δ*pan*CD Δ*leu*CD Δ*arg*B), *ribH* conditional gene silencing achieved by ATc induction. | This study |
| *E.coli* DH5-α | Obtained from NEB (T1 phage resistant and *endA* deficient) | https://international.neb.com/products/c2987-neb-5-alpha-competent-e-coli-high-efficiency#Product%20Information |
| *E.coli* BL21 (DE3) | *E. coli* strain, suitable for transformation and protein expression from plasmids with T7 promoter. | https://international.neb.com/products/c2527-bl21de3-competent-e-coli#Product%20Information |

**Supplementary Table 4. List of primers used in the study.**

| **Primer name** | **Gene number and description** | **Sequence (5'-3')** | **Size of primer (bp)** | **Experiments** |
| --- | --- | --- | --- | --- |
| *ribH*_RT_F | *Rv1416,* *ribH*  *6,7-dimethyl-8- ribityllumazine synthase (lumazine synthase)* | CAATCATGATGCCGTCGT | 18 | For RT-PCR analysis |
| *ribH*_RT_R |  | GGGTCAGTCCCTGGGTTAC | 19 |  |
| *SigA*_RT_F | Rv2703, *sigA*  RNA polymerase sigma factor SigA (sigma-A) | ATCGCTGAACCCACCGAAAA | 20 | For RT-PCR analysis |
| *SigA*_RT_R |  | GACCTCTTCCTCGGCGTTG | 19 |  |
| *ribH*_F_P | Rv1416, *ribH*  *6,7-dimethyl-8- ribityllumazine synthase (lumazine synthase)* | GGGGCATATGAAGGGTGGCGCCGGGGT | 27 | PCR amplification for cloning of *ribH* in pet28a |
| *ribH*_R_P |  | GGGGCTCGAGTAGTCACGAGTGAGCGCGCAGCT | 33 |  |
| Cr_UP | *Rv1416*, *ribH*  *6,7-dimethyl-8- ribityllumazine synthase (lumazine synthase)* | GATCTTTCCGTGCCAGCTG | 19 | Guide sequence targeting *ribH* |
| Cr_DN |  | CGCAGCTGGCACGGAAAGATCCATG | 25 |  |
| *Kan*^r^ F | *Kan*^r^  Kanamycin resistance gene cassette | GAGAAAACTCACCGAGGCAG | 20 | For screening clones carrying *kan*^r^ plasmid |
| *Kan*^r^ F |  | GTATTTCGTCTCGCTCAGGC | 20 |  |
| *Hyg^r^* F | *Hyg*^r^  Hygromycin resistance gene cassette | CCGGGCTCGCAGCAGCGGGC | 20 | For screening clones carrying *hyg*^r^ plasmid |
| *Hyg^r^* R |  | CCTCGAACACCTCGAAGTCG | 20 |  |

*Rv- notation is used for gene numbers in M. tb H37Rv*


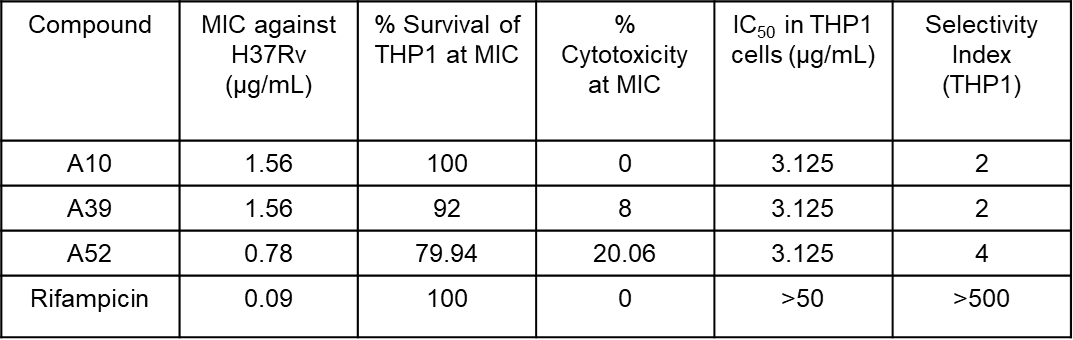


**Supplementary Table 5. *In vitro* cytotoxicity and selectivity index of compounds A10, A39 and A52 against THP1 human monocyte derived macrophages.** Cytotoxicity of the RibH targeting drugs was measured by MTT assay against THP1 human monocyte derived macrophages and data (n=3) was analysed and expressed as IC_50_ (half-maximal inhibitory concentration). Selectivity index (SI) = IC_50_ (mammalian cells)/MIC (*M. tb* H37Rv).


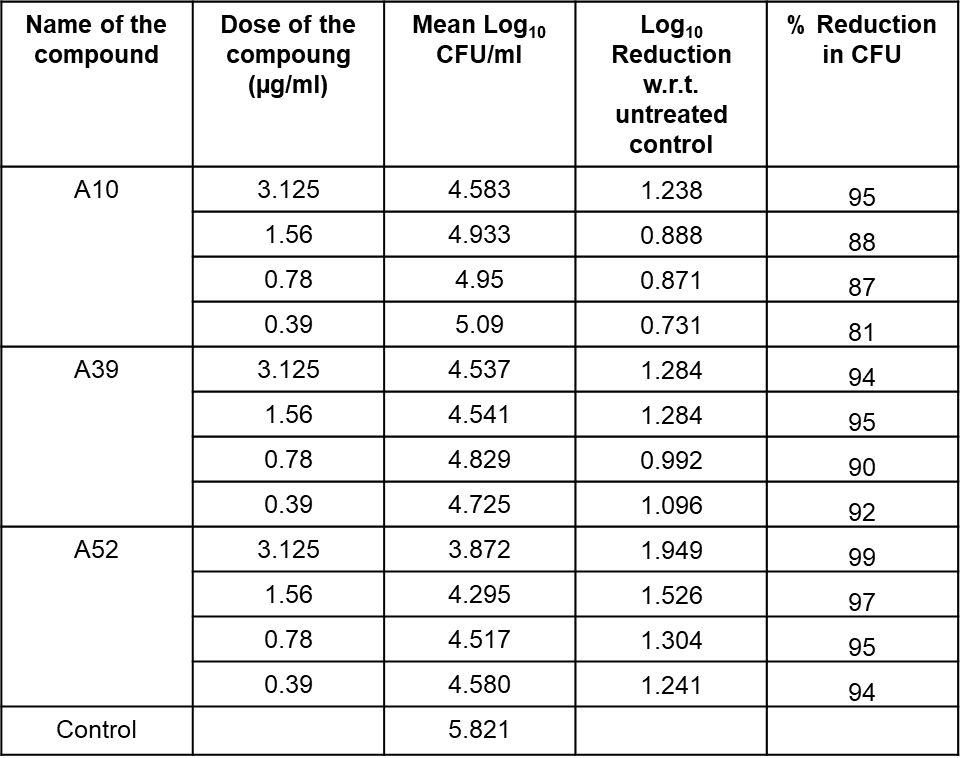


**Supplementary Table 6: Intracellular antimycobacterial activity of short-listed compounds in THP-1 derived human macrophages.** Table depicts CFU obtained on plating of infected THP-1 derived macrophages post five-days treatment with three different concentrations of A10, A39 and A52. THP-1 cells were infected with *M. tb*. H37Rv at a MOI of 1:5. Infected macrophages were treated with A10 or A39 at a concentration above MIC (3.125 µg/ml), MIC (1.56 µg/ml) and sub-MIC (0.78 µg/ml); or A52 at two concentrations above MIC (3.125 µg/ml-1.56 µg/ml) and MIC (0.78 µg/ml). CFU data (n = 3) is represented as mean±s.d log_10_ CFU/ml. Reduction in CFU was calculated with respect to (w.r.t) untreated control. Among the three compounds tested, A52 showed the most potent activity in terms of reducing the intracellular mycobacterial burden.

**Supplementary Methods**

**Synthesis of compounds**

**Supplementary Table 7. Molecular weight, SMILES ID and structure of A10, A39 and A52**

| **S. No.** | **Database Code** | **Tube Code** | **Mol. weight** | **Simplified molecular-input line-entry system**  **(SMILES) ID** | **Structure** |
| --- | --- | --- | --- | --- | --- |
| 1 | NR-310 | A10 | 315.30 | O=C(/C(S/1)=C\C2=CC=C([N+]([O-])=O)O2)NC1=N\C3=CC=CC=C3 | 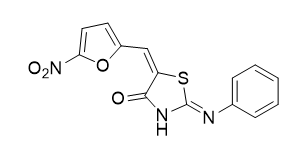 |
| 2 | NR-340 | A39 | 349.75 | ClC1=CC=C(/N=C2NC(/C(S/2)=C\C3=CC=C([N+]([O-])=O)O3)=O)C=C1 | 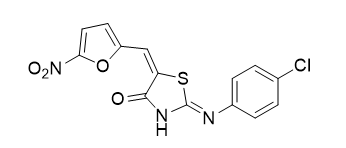 |
| 3 | NR 353 | A52 | 329.33 | CC1=CC=C(/N=C2NC(/C(S/2)=C\C3=CC=C([N+]([O-])=O)O3)=O)C=C1 | 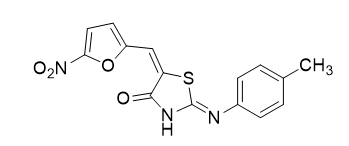 |

**Synthesis scheme of 2-(phenylimino) thiazolidin-4-one derivatives**

The target molecules were synthesized by following the three-step synthetic protocol. Synthesis started with the commercially available various substituted aryl isothiocyanates on reaction with ammonia solution in THF and the resulting solid was filtered and washed with excess of water, cold ethanol and diethyl ether and dried to afford the corresponding substituted arylthioureas (**TZ_02a-e**). Further the cyclization reaction was carried out by the reaction of (**TZ_02a-e**) with ethyl 2-bromoacetate and anhydrous NaOAc in absolute ethanol at 60 °C to afford the key intermediate 2-(substituted aryimino) thiazolidin-4-one (**TZ_03a-e**) in good yields. These reactions were also successfully carried out using ethyl-2-chloroacetate, anhydrous NaOAc in absolute ethanol at 60 °C but the former reaction conditions resulted in good yields. In final step we used Knoevenagal condensation of the compound **TZ_03a-e** with various substituted aldehydes using piperidine in absolute ethanol at 60 °C, upon complete consumption of the starting material the reaction mixture was filtered to remove the bromide salts and the filtrate was concentrated and obtained residue was re-dissolved in ethyl acetate and washed with water and brine solution and purified to produce title compounds **TZ_04-TZ_56.**

**General procedure for the synthesis of (TZ_02a-e)**

To the starting material **TZ_01a-e** (1.0 equiv) in THF, was added NH_3_ solution (10 vol) under cooling conditions and allowed the reaction mixture to stir at room temperature for 3 h, then the solids formed in the reaction mixture was filtered and washed with diethyl ether and dried to afford the pure product **(TZ_02a-e)** as white solid (yields 90-95%).

**1-Phenylthiourea (TZ_02a)**

Following the general procedure, the product was synthesized from phenyl isothiocyanate **TZ_01a** (3.00 g, 22.22 mmol), NH_3_ solution (30 mL) produced 1-phenylthiourea **(TZ_02a**) (3.2 g, 98%) as white solid. ESI-MS showed 153 [M+H]^+^ and carried to next step.

**1-(*p*-Tolyl) thiourea (TZ_02b)**

Following the general procedure the product was synthesized from p-tolyl isothiocyanate **TZ_01b** (5.00 g, 33.55 mmol), NH_3_ solution (50 mL) produced 1-(p-tolyl) thiourea **(TZ_02b**) (5.4 g, 98%) as white solid. ESI-MS showed 167 [M+H]^+^ and carried to next step.

**1-(4-Chlorophenyl)thiourea (TZ_02d)**

Following the general procedure the product was synthesized from 4-chlorophenylisothio cyanate **TZ_01d** (5.00 g, 29.58 mmol), NH_3_ solution (50 mL) produced 1-(4-chlorophenyl) thiourea **(TZ_02d**) (5.3 g, 96%) as white solid. ESI-MS showed 187 [M+H]^+^ and carried to next step.

**General procedure for the synthesis of (TZ_03a-e)**

To the stirred solution of **TZ_02a-e** (1.0 equiv) in EtOH (10 vol) was added anhydrous NaOAc (5.0 equivalents) followed by ethyl bromoacetate (2.0 equivalents) and the reaction mixture was heated at 60 °C for 7 h. The solids formed in the reaction mixture were filtered and washed with EtOH, the filtrate was concentrated and the solid obtained was partitioned between ethyl acetate and water then washed with brine solution, the Ethyl acetate layer was dried over anhydrous Na_2_SO_4_ and concentrated under vacuo then the solid obtained was triturated with CH_2_Cl_2_/hexanes the resulting solid was filtered, washed with diethyl ether and dried to get **TZ_03a-e** in pure form (yields >80%) and used for the next step.

**2-(Phenylimino) thiazolidin-4-one (TZ_03a)**

Following the general procedure, the product was synthesized from 1-phenylthiourea **TZ_02a** (3.20 g, 21.05 mmol), anhydrous NaOAc (8.63 g, 105.05 mmol) and Ethyl bromoacetate (4.65 mL, 42.10 mmol) produced 2-(phenylimino) thiazolidin-4-one **(TZ_03a**) (3.6 g, 89%) as yellow solid. ESI-MS showed 193 [M+H]^+^_._ ^1^H NMR (400 MHz, DMSO-*d*_6_) δ 11.73 (s,0.5×1H), 11.15 (s,0.5×1H), 7.70 (m, 1H), 7.40–7.37 (m, 2H), 7.18–7.13 (m, 1H), 7.00 (m, 1H), 4.01 (s, 1H), 3.97 (s, 1H).

**2-(p-Tolylimino) thiazolidin-4-one (TZ_03b)**

Following the general procedure, the product was synthesized from 1-(p-tolyl) thiourea **(TZ_02b**) (5.40 g, 32.53 mmol), anhydrous NaOAc (13.33 g, 162.65 mmol) and ethylbromoacetate (7.19 mL, 65.06 mmol) produced 2-(p-tolylimino) thiazolidin-4-one **(TZ_03b**) (5.8 g, 86%) as yellow solid. ESI-MS showed 207 [M+H]^+^_._ ^1^H NMR (400 MHz, DMSO-*d*_6_) δ 11.33 (s, 1H), 7.56 (d, *J* = 7.4 Hz, 1H), 6.91 (d, *J* = 7.6 Hz, 1H), 3.98 (s, 1H), 3.91 (s, 1H), 2.28 (s, 3H).

**2-((4-Chlorophenyl) imino) thiazolidin-4-one (TZ_03d)**

Following the general procedure, the product was synthesized from 1-(4-chlorophenyl) thiourea

**TZ_02d** (5.30 g, 28.49 mmol), anhydrous NaOAc (11.68 g, 142.47 mmol) and ethyl bromoacetate (6.30 mL, 56.98 mmol) produced 2-((4-chlorophenyl) imino) thiazolidin-4-one **(TZ_03d**) (5.4 g, 84%) as a brown solid. ESI-MS showed 227 [M+H]^+^ _._ ^1^H NMR (300 MHz, CDCl_3_) δ 9.01 (s, 1H), 7.54–7.46 (bm, 4H), 3.54 (s, 2H).

**General procedure for the synthesis of compounds TZ_04** – **TZ_56**

To the stirred solution of **TZ_03a-e** (1.0 equivalents) in EtOH, was added piperidine (1.0 equivalents) and RCHO (1.2 equivalents) and heated at 60°C for 12 h, then the solids formed in the reaction mixture were filtered and washed with EtOH, hexanes to afford the pure product in good yields.

**5-((5-Nitrofuran-2-yl) methylene)-2-(phenylimino) thiazolidin-4-one (TZ_13):** Yield: 65%; m.p. 241–242 °C; MS(ESI) *m/z* 316 [M+H]^+^. ^1^H NMR (400 MHz, CDCl_3_) δ 9.93 (s, 1H), 8.31 (d, *J* = 8.0 Hz, 1H), 7.72–7.63 (m, 2H), 7.58 (s, 1H), 7.54 (d, *J* = 8.0Hz, 1H), 7.49–7.32 (m, 3H); ^13^C NMR (100 MHz, CDCl_3_) 176.3, 164.2, 156.0, 146.2, 135.2, 133.1, 132.0, 130.7(2C), 127.2, 125.9(2C), 124.2, 119.3. Anal calcd for C_14_H_9_N_3_O_4_S: C, 53.33; H, 2.88; N, 13.33% Found C, 53.42; H, 2.92; N, 13.45%.

**5-((5-Nitrofuran-2-yl) methylene)-2-(*p*-tolylimino) thiazolidin-4-one (TZ_24):** Yield: 61%; m.p. 229–230 °C; MS(ESI) *m/z* 330 [M+H]^+^. ^1^H NMR (300 MHz, DMSO-*d*_6_) δ 9.20 (s, 1H), 8.17 (d, *J* = 8.6 Hz, 1H), 7.70–7.61 (m, 4H), 7.55 (d, *J* = 8.0 Hz, 2H), 2.47 (s, 3H); ^13^C NMR (75 MHz, DMSO-*d*_6_) 176.2, 164.1, 155.9, 146.2, 135.2, 133.0, 132.1, 130.8(2C), 127.1, 125.7(2C), 124.1, 119.1, 25.2. Anal calcd for C_15_H_11_N_3_O_4_S: C, 54.71; H, 3.37; N, 12.76% Found C, 54.77; H, 3.42; N, 12.81%.

**2-((4-Chlorophenyl) imino)-5-((5-nitrofuran-2-yl) methylene) thiazolidin-4-one (TZ_44):** Yield: 61%; m.p. 211–212 °C; MS(ESI) *m/z* 350 [M+H]^+^. ^1^H NMR (300 MHz, DMSO-*d*_6_) δ 9.20 (s, 1H), 8.11 (d, *J* = 8.8 Hz, 1H), 7.73–7.62 (m, 4H), 7.58 (d, *J* = 8.0 Hz, 2H); ^13^C NMR (75 MHz, DMSO-*d*_6_) 178.4, 166.3, 156.7, 146.9, 136.1, 134.0, 132.7, 131.6(2C), 129.1, 126.6(2C), 124.8, 119.8. Anal calcd for C_14_H_8_ClN_3_O_4_S: C, 48.08; H, 2.31; N, 12.01% Found C, 48.14; H, 2.38; N, 12.06%.

**Supplementary Table 8: R group description, yield, melting point (MP), molecular formula and weight of the shortlisted compounds**

| **Compound** | **Compound Code** | **R** | **Yield (%)** | **M.P.**  **(**°C**)** | **Molecular formula** | **Molecular weight** |
| --- | --- | --- | --- | --- | --- | --- |
| TZ_13 | A10 | 5-Nitro-2-furyl | 65 | 241-242 | C_14_H_9_N_3_O_4_S | 315.30 |
| TZ_44 | A39 | 5-Nitro-2-furyl | 61 | 211-212 | C_14_H_8_ClN_3_O_4_S | 349.75 |
| TZ_24 | A52 | 5-Nitro-2-furyl | 61 | 229-230 | C_15_H_11_N_3_O_4_S | 329.33 |

**Cloning, expression and purification of RibH**

**Cloning:** In order to obtain purified lumazine synthase enzyme of *M. tb*, the gene encoding lumazine synthase *ribH* (*Rv1416*) was PCR amplified using H37Rv genomic DNA as template and oligonucleotides (5’ GGGGCATATGAAGGGTGGCGCCGGGGT 3’) as forward primer and (5’GGGGCTCGAGTAGTCACGAGTGAGCGCGCAGCT 3’) as reverse primer. The amplified PCR product was digested using restriction enzymes *Nde* I and *Xho* I (New England Biolabs) and ligated using The Quick Ligation™ Kit (New England Biolabs) with the *E. coli* expression vector pET28(a) (Novagen) digested with the same set of restriction enzymes. The ligated plasmid DNA was transformed in to DH5α *E. coli* strain (New England Biolabs) and colonies were selected on 25 µg/ml kanamycin (Himedia). The clones were confirmed by PCR amplification, restriction digestion and sequencing. Following this, the sequence verified plasmid DNA was transformed into BL21 (DE3) (New England Biolabs) and colonies were selected on 25 µg/ml kanamycin (Himedia) and confirmed by PCR amplification.

**Induction condition optimization:** The expression vector offers an inducible expression system wherein, protein of interest with a 6x-his tag at N-terminus is expressed under the control of T7 promoter and lac operator. Addition of optimised concentration of Isopropyl β-d-1-thiogalactopyranoside (IPTG) leads to expression of protein of interest. In order to optimise the expression of lumazine synthase (16 kDa), BL21(DE3) clones were grown in LB media with kanamycin with various concentrations of IPTG (0.2 – 0.8 mM) at various temperature conditions (20-37^o^C) for 4 hours-overnight. The induction conditions offering maximum production of properly folded protein in soluble fraction (0.2 mM IPTG at 16°C overnight incubation) was utilised for final purification. The protein was purified by affinity chromatography using Ni-NTA resin using a gradient concentration of imidazole in the range of 100-300 mM as per manufacturer’s specification (Clontech Takara Ni-NTA Resin_ 635660). The fraction giving the maximum purity (250 mM) was pooled and dialyzed against a 50 mM Potassium phosphate buffer (pH 7.0) containing 10% glycerol. The purified protein quality was assessed by various methods such as, sodium dodecyl sulphate polyacrylamide gel electrophoresis (SDS PAGE), circular dichroism (CD) and dynamic light scattering (DLS). The protein was flash frozen using liquid nitrogen followed by long term storage at -80^o^C.

**Binding affinity determination using Microscale Thermophoresis (MST) assay**

To determine the binding affinity, MST assay was carried out using Monolith NT.115 microscale thermophoresis instrument (Nano Temper Technology). In this method, ligand and protein interaction is studied by employing Monolith His-tag labelling kit RED-tris-NTA 2^nd^ Generation (Nano Temper Technology) as per the manufacturer’s specification and as described previously^2^. This method relies on the high-affinity but non-covalent interaction of RED-tris-NTA fluorescent dye (containing flour RED - NT647) with six histidine-tag of the protein of interest. This conjugate provides specific binding and a high fluorescence signal with best signal-to-noise ratio even in complex samples such as cell lysates which often auto fluoresce in the blue and green part of the spectrum. When such a labelled protein is subjected to temperature gradient, it leads to thermophoresis, which is the directed motion of molecules in response to infrared light induced temperature gradient. Thermophoresis depends on molecule size, charge, hydration shell and the extent of ligand binding as described^2,3^.

***Protein labelling****:* Purified his-tagged RibH protein was diluted in 1X PBST (Phosphate buffered saline with 0.05% tween 20) at a final concentration of 200 nM. RED-tris-NTA fluorescent dye (5 µM) was diluted in 1X PBST at a final concentration of 100 nM. For labelling, equal volume of dye and protein (90 µl each) were mixed and incubated at room temperature for 30 minutes followed by centrifugation at 15000 g, 4
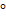
C for 10 minutes. The supernatant containing the labelled protein was then transferred to a fresh tube.

***Ligand preparation****:* For binding assay, riboflavin (positive control), A10, A39 and A52 compounds were dissolved in autoclaved milli Q water to a final concentration of 1 µM. Next, 1µM of the ligands were then two-fold serially diluted 16 times. 10 µl of 100 nM labelled protein was then mixed with 10 µl of serially diluted compounds (1000 nM- 0.03 nM).

***Protein and Ligand binding****:* The final concentration of 50 nM protein was titrated against the final ligand concentrations of 500 nM-0.015 nM in 16 different capillary tubes.

***MST analysis:*** MST experiment was run for 20 seconds using Monolith NT.115 microscale thermophoresis instrument at room temperature (23
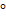
 to 25
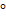
C) using an inbuilt software called Monolith NT Control Software. Capillary scan images were collected at 10^th^ second and NT Analysis Software was then used for a precise analysis of microscale thermophoresis data and quantification of dissociation constant (*K*_d_) as per the manufacturer’s instructions (Nano Temper Technology, <https://www.uni-hohenheim.de/fileadmin/einrichtungen/mst/Manual_NT115.pdf>)

**Riboflavin Displacement Assay**

As described by Chen *et al*, displacement of riboflavin bound with lumazine synthase can be assessed based on its fluorescence^4^. Free riboflavin fluoresces whereas, enzyme bound riboflavin does not. The short-listed compounds could possibly compete with riboflavin for the same binding site in *M. tb* RibH and an increase in the fluorescence intensity would indicate displacement of riboflavin. To assess the displacement of bound riboflavin, the *M. tb* RibH (28 µM) was pre-complexed with ~0.966 equivalent of riboflavin (27.04 µM) and stored at -20 °C. The stock solution was thawed on ice and diluted to 8 µM with 50 mM phosphate buffer, pH 7.0 before use. 25 µg/ml solutions of shortlisted compounds, A10, A39 and A52 were prepared in the same buffer. 20 µl of the compound solutions was added to a black flat-bottomed 96-well plate (Thermo Scientific™). 20 µl of the enzyme-riboflavin complex was added to the compound solution. Each well had a final volume of 40 µl and final concentrations of the protein, riboflavin and the compounds were 4, 3.86 µM and 12.5 µg/ml, respectively. The assay was performed in triplicates and the 96-well plate contained three wells each of buffer, 2.5% DMSO, 3.86 µM riboflavin, 4 µM enzyme-riboflavin complex and 12.5 µg/ml of compounds as controls. Fluorescence was recorded at excitation and emission wavelengths of 440 nm and 530 nm, respectively. Percentage of displaced riboflavin from bound enzyme-riboflavin complex was calculated as follows:

A= FL Riboflavin

B= FL (Riboflavin + RibH protein) Complex

C= FL Complex + Compound

% Bound= {(A-B)/A} × 100

% Displaced= {(C-B)/B} × 100

**Nucleotide extraction and flavin adenine dinucleotide quantification by high-performance liquid chromatography**

Nucleotide extraction for HPLC was carried out as described^5^. Briefly, 5 mL extraction buffer containing acetonitrile, methanol and water (2:2:1) was prepared and 12.5 µl of 1 µM cXMP was added as an internal technical control. *M. tb* was grown to mid-log phase and treated with the short-listed compounds at their respective MICs (A10 and A39: 1.56 µg/mL, and A52: 0.78 µg/mL) for 24 hours. 10 mL of the treated and the untreated cultures were harvested by centrifugation and the culture pellets were weighed followed by resuspension in the ice-cold extraction buffer. The suspensions were incubated for 15 minutes on ice followed by boiling at 100°C for 10 minutes. The mixture was cooled down and extracted after quick centrifugation. Extraction was repeated twice as described above. Pooled samples were vacuum dried and resuspended in 400 µl distilled water followed by detection and quantification by HPLC. Briefly, the chromatographic separation was performed on a Shimadzu HPLC system with photodiode array (PDA) detector on Phenomenex reverse-phase column (C18, 150 × 4.6 mm), particle size 5 µM, injection volume 10 µl, column oven temperature 25°C with run time of 15 minutes. An isocratic flow rate was programmed using mobile phase A Water (pH 5.5 adjusted with acetic acid) and B acetonitrile. Flow rate 1 ml/minute with gradient ratio 40:60 was used. Flavin adenine dinucleotide (FAD) with ≥95% purity was purchased from Sigma and standard solutions of different concentrations were freshly prepared under dimmed light and passed through 0.22 µm disposable filters. FAD was detected at a wavelength of 450 ± 2 nm. Sample peaks monitored by PDA detection were identified by their retention times with comparison to the standard peak. Area under the curve was used to quantify FAD levels in the treated and untreated samples of extracted nucleotides. Area ratio was calculated by normalizing the AUC of all groups with respect to their respective AUC of cXMP. The normalized values were then plotted for comparative analysis.

**Computational Pipeline to determine the conserved nature of drug binding pocket in RibH**

Nucleotide sequences for the lumazine synthase (RibH) gene were obtained from NCBI’s nucleotide and genome databases for various species of the Mycobacterium tuberculosis complex as well as non-tuberculous mycobacteria. The protein sequences for RibH were obtained from UniProt. Nucleotide sequence alignment for various species were carried out against the *rib H* (*Rv 1416*) sequence of *Mycobacterium tuberculosis* (strain ATCC 25618 / *H37Rv*), using the BlastN suite of NCBI to obtain % similarity and % identity. We then carried out multiple sequence alignment of the RibH protein sequence using CLUSTAL O and T-Coffee.

In order to determine % similarity and % identity in protein sequences, EMBOSS NEEDLE was used to compare the sequence from various species against *M. tb* (strain ATCC 25618 / H37Rv). We then constructed a phylogenetic tree based on RibH protein sequence using T-Coffee. For the organisms, for which protein sequences were unavailable on UniProt, Blast X was used to find the protein sequences from GenBank.

In order to find if the drugs discovered by us could potentially be used to treat other mycobacterial infections, we manually analysed the aligned protein sequences of RibH from various organisms to find the identical residues within the drug binding and co-crystal ligand binding pockets. The drug binding pocket and the residues present thereof within the pocket were obtained using the Maestro suite of Schrodinger, and the residues present in the co-crystal ligand binding pocket were identified from PDBSum. The % identity of the residues in both pockets was tabulated in Supplementary Table 4.

**Pipeline of computational studies**

**Web resources used**

UniProt - <https://www.uniprot.org/>

NCBI - <https://www.ncbi.nlm.nih.gov/>

GenBank – <https://www.ncbi.nlm.nih.gov/genbank/>

PDBSUM – <http://www.ebi.ac.uk/thornton-srv/databases/pdbsum/>

MAESTRO (Schrodinger) - <https://www.schrodinger.com/products/maestro>

BLASTN – <https://www.ncbi.nlm.nih.gov/geo/query/blast.html>

BLASTX – <https://blast.ncbi.nlm.nih.gov/Blast.cgi?PROGRAM=blastx&PAGE_TYPE=BlastSearch&LINK_LOC=blasthome>

T-Coffee – <https://www.ebi.ac.uk/Tools/msa/tcoffee/>

EMBOSS NEEDLE – <https://www.ebi.ac.uk/Tools/psa/emboss_needle/>

**Supplementary References**

1 Daina, A., Michielin, O. & Zoete, V. SwissADME: a free web tool to evaluate pharmacokinetics, drug-likeness and medicinal chemistry friendliness of small molecules. *Scientific reports* **7**, 42717 (2017).

2 Bartoschik, T. *et al.* Near-native, site-specific and purification-free protein labeling for quantitative protein interaction analysis by MicroScale Thermophoresis. *Sci Rep* **8**, 4977, doi:10.1038/s41598-018-23154-3 (2018).

3 Wienken, C. J., Baaske, P., Rothbauer, U., Braun, D. & Duhr, S. Protein-binding assays in biological liquids using microscale thermophoresis. *Nat Commun* **1**, 100, doi:10.1038/ncomms1093 (2010).

4 Chen, J. *et al.* A high-throughput screen utilizing the fluorescence of riboflavin for identification of lumazine synthase inhibitors. *Anal Biochem* **338**, 124-130, doi:10.1016/j.ab.2004.11.033 (2005).

5 Dey, B. *et al.* A bacterial cyclic dinucleotide activates the cytosolic surveillance pathway and mediates innate resistance to tuberculosis. *Nat Med* **21**, 401-406, doi:10.1038/nm.3813 (2015).
